# Genetic Divergence in *Eucalyptus camaldulensis* Progenies in the Savanna Biome in Mato Grosso, Brazil

**DOI:** 10.1371/journal.pone.0163698

**Published:** 2016-09-28

**Authors:** Reginaldo Brito da Costa, Jeane Cabral da Silva, Leandro Skowronski, Michel Constantino, Hemerson Pistori, Jannaína Velasques da Costa Pinto

**Affiliations:** 1 Graduate Program in Environmental Sciences and Sustainable Agro-livestock Technologies, Catholic University Dom Bosco, Campo Grande, Mato Grosso do Sul, Brasil; 2 Graduate Program in Forestry and Environmental Sciences, University of Mato Grosso, Cuiabá, Mato Grosso, Brasil; 3 Graduate Program in Biotechnology, Catholic University Dom Bosco, Campo Grande, Mato Grosso do Sul, Brasil; Institute of Genetics and Developmental Biology Chinese Academy of Sciences, CHINA

## Abstract

Assessing the parental genetic differences and their subsequent prediction of progeny performance is an important first step to assure the efficiency of any breeding program. In this study, we estimate the genetic divergence in *Eucalyptus camaldulensis* based on the morphological traits of 132 progenies grown in a savanna biome. Thus, a field experiment was performed using a randomized block design and five replications to compare divergences in total height, commercial height, diameter at breast height, stem form and survival rate at 48 months. Tocher’s clustering method was performed using the Mahalanobis and Euclidian distances. The Mahalanobis distance seemed more reliable for the assessed parameters and clustered all of the progenies into fourteen major groups. The most similar progenies (86 accessions) were clustered into Group I, while the most dissimilar (1 progeny) represented Group XIV. The divergence analysis indicated that promising crosses could be made between progenies allocated in different groups for high genetic divergence and for favorable morphological traits.

## Introduction

In many countries, Eucalyptus has become an important wood crop, especially economically, associated with rapid growth, biomass yield and bioenergy feedstock. In Brazil alone, Eucalyptus plantations occupy approximately 5.1 million hectares [1] and contribute to the foreign trade surplus, especially in the pulp and paper industries.

Thus, the establishment of breeding programs with forest species is important for attaining greater productivity and wood quality without jeopardizing the genetic heritage of the elected populations [2, 3].

Knowledge of genetic diversity is important for any breeding strategy and provides a scientific basis for the better management of the genetic heritage of forest species [4]. On the other hand, clustering progeny according to similarity has become essential for selecting the crossing cultivars based on quantitative traits of economic interest [5, 6].

*Eucalyptus camaldulensis* is a native species from Australia and is distributed among tropical and subtropical regions, where it is widely cultivated for its short rotation cycle. This species is mostly grown for wood, pulp, construction, biofuel and restoration of degraded land and has also been used in breeding programs to generate cultivars with greater tolerance to drought and salinity [7] and greater resistance to pests and diseases.

For years, the use of multivariate analysis has been an important tool for genetic diversity studies, helping to organize germplasm banks and determine breeding strategies for different wood species: *Pinus caribaea* [8], *Psidium guajava* [9], *Eucalyptus tereticornis* [10], and *Dalbergia sissoo* [11], among others. However, for the savanna biome in the state of Mato Grosso, there is a lack of information on *E*. *camaldulensis* despite its intensive management.

In this context, the present study aimed to estimate the genetic divergence in *E*. *camaldulensis* based on the morphological traits of progeny grown in the savanna biome in Mato Grosso (Brazil). We used Tocher's clustering method considering the Mahalanobis and Euclidean distances to support the selection of suitable parents with maximum heterosis and complementary genes.

## Materials and Methods

### Progeny and research area

The seedlings of *E*. *camaldulensis* were provided by the Faculty of Engineering, UNESP Ilha Solteira. Originally, the seeds were collected from open-pollinated trees in Katherine River, Australia, and obtained in partnership with EMBRAPA Forests.

The progeny test was carried in the experimental field of IFMT (Federal Institute of Education, Science and Technology Mato Grosso—Campus São Vicente) in the municipality of Santo Antonio Leverger in the savanna region of Mato Grosso, Brazil. The geographic characteristics are presented in Table 1.

**Table 1:** Ecogeographic characteristics of the experimental site in a savanna biome from Mato Grosso, Brazil.

Descriptive Variables	Local Coordination
Altitude (m)	750
Latitude	15°49’21” S
Longitude	55°25’06” W
Climate (Köppen)	Aw
Temperature^a^ (°C)	20
Rainfall^a^ (mm)	2.000

^a^Annual Average

**Fig 1:**
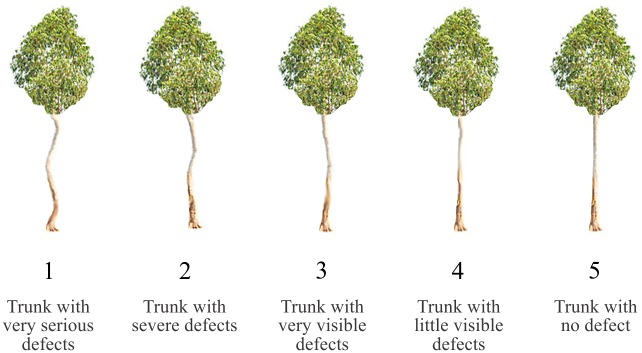
Grade of stem form used for morphological analysis of *E*. *camaldulensis* in a savanna biome from Mato Grosso, Brazil.

At 48 months, the following morphological traits were evaluated: diameter at breast height (DBH); total height (TH); commercial height (CH); stem form (SF) and survival rate (SR). The stem form grade was evaluated as shown in Fig 1 [12].

### Statistical analysis

The experiment was performed using a randomized block design with 132 treatments (progenies), with five replications and three plants per plot. The plants were placed in single rows at a spacing of 3 m x 2 m, totaling 660 plots and 1,980 individuals.

The genetic parameters related to narrow heritability on a progeny basis and the genotypic values were analyzed using the restricted maximum likelihood/best linear unbiased prediction (REML/BLUP) [13, 14, 15] with the following univariate model of specific combined ability: *y* = *Xb* + *Za* + *Wc* + *e*, where y is the data vector, *b* is the block effect (fixed), *a* is the additive genetic effect (random), *c* is the permanent plot effect (random), and *e* is the random error. X, Z and W are the incidence matrices for b, a and c, respectively.

To estimate the genetic diversity among the progenies, we used a multivariate analysis via Tocher’s method considering the Mahalanobis and Euclidean distances to determine the genetic dissimilarity and clusters based on five quantitative morphological traits. All of the procedures were performed using the Selegen computer program [13, 14, 15, 16, 17].

### Euclidean Distance

Euclidean distance is the square root of the sum of the squared difference between two profiles in a v-space, where v is the number of variables or coordinates in a space. Mathematically, it is given by $D_{E}(i,i´)={\left[\sum_{j=1}^{v}{\left(u_{ij}-u_{i´j}\right)}^{2}\right]}^{1/2}$, where i and i' are the two profiles, and u_ij_ and u_i'j_ are the values of the variable j observed for i and i', respectively.

Frequently, when the number of variables is high, the values obtained by this expression are also very high. Thus, in practice, two alternative forms to estimate this distance are commonly used: 1. the mean Euclidean distance given by $D_{EM}(i,i´)={\left[\sum_{j=1}^{v}{\left(u_{ij}-u_{i´j}\right)}^{2}/v\right]}^{1/2}$, and 2. the square of the mean Euclidean distance given by $D_{QEM}(i,i´)=(\sum_{j=1}^{v}{\left(u_{ij}-u_{i´j}\right)}^{2})/v$.

The Euclidean distance is not invariant to scale effects. Thus, the variables that were used in u_ij_ and u_i'j_ must first be standardized [13].

### Statistical or Mahalanobis Distance

The Mahalanobis distance or statistical distance was proposed in 1936 [18] and differs from the Euclidean distance by considering the correlations between variables and by being invariant to the scale effects.

Considering a) the vectors of the averages of v (variables) for i, u_i_ = (u_i1_, u_i2_, u_i3_…. i_iv_) and for i', u_i'_ = (u_i'1_, u_i'2_, u_i'3_…. i_i'v_) and b) the covariance matrix between the variables, the Mahalanobis distance is given by *D*_*M*_ (*i*, *i*′) = [(*u*_*i*_ − *u*_*i*′_)′Σ^−1^(*u*_*i*_ − *u*_*i*′_)]^1/2^.

If the covariance matrix is an identity matrix (i.e., the variables are not correlated with units), the Mahalanobis distance can simply be estimated from the Euclidean distance. However, if the covariance matrix is a diagonal matrix (uncorrelated variables), then the Mahalanobis distance is estimated using a standardized Euclidean distance formula: $D_{E}(i,i´)={\left[\sum_{j=1}^{v}{\left(u_{ij}-u_{i´j}\right)}^{2}/\sigma_{j}^{2}\right]}^{1/2}$.

The Mahalanobis distance is also closely related to Hotelling's T^2^ statistic used for multivariate comparison of means. The use of the Mahalanobis distance is preferred over the use of the Euclidean distance, as the first considers the correlations between variables.

### Grouping by Tocher's method

Clustering techniques aim to group individuals considering their heterogeneity and homogeneity. Different clustering techniques can be subdivided into two classes: hierarchical methods and mutually exclusive methods. Hierarchical methods lead to dendograms, while mutually exclusive methods lead to different groups that are not connected to each other; mutually exclusive methods are faster and more easily interpreted.

Among the mutually exclusive methods, Tocher's grouping method is the best [19]. This method has been widely used in plant breeding and assumes that the average of distances within groups must be less than the average among groups. Initially, a pair of closely correlated individuals is identified, forming the first group. The same criteria are used to identify whether new individuals can be allocated in the same group. If they cannot be included in the same group, then a new group is formed.

The mean intra-group distance increases with the addition of a new individual to the group. To accept the entry of a new individual, this increase in the mean intra-group distance (*D*(*ij*)*k*/*n*) must be compared with a maximum allowable threshold for inclusion in the group.

This ceiling (max) is usually considered the greatest distance of all of the minimum distances associated with each individual.

Mathematically, it is expressed by:

1. inclusion of the individual k in the group: if *D*(*ij*)*k*/*n* ≤ *max*.
2. non-inclusion of individual k in the group: if *D*(*ij*)*k*/*n* > *max*.

The number n refers to the number of individuals already allocated to the group and is given by *D*(*ij*)*k* = *Dik +Djk*

## Results

Considering the genetic parameters related to heritability on a progeny basis and the genotypic values, individual heritability ($h_{a}^{2}$) was moderate for DBH (0.16) and low for TH (0.08), CH (0.05), SF (0.07) and SR (0.12), as shown in Table 2.

**Table 2:** Genetic parameters estimation for morphological traits of *E*. *camaldulensis* progenies in a savanna biome from Mato Grosso, Brazil.

Estimates	DBH (cm)	TH(m)	CH (m)	SF	SR (%)
$h_{a}^{2}$	0.1660	0.0822	0.0521	0.0684	0.1234
$h_{mp}^{2}$	0.2825	0.1356	0.0938	0.1634	0.3037
*CV*_*gi*_%	16.5691	8.2243	7.7475	3.3262	10.3418
*CV*_*gp*_%	8.2178	4.1121	3.8737	1.6631	5.1709
*CV*_*e*_%	29.5185	23.2063	26.9118	8.4142	17.5044
*CV*_*r*_	0.2806	0.1772	0.1439	0.1976	0.2954
*Ac*_*prog*_	0.5315	0.3683	0.3063	0.4042	0.5511
**General Mean**	8.3967	8.5453	5.9954	4.5623	0.9196

$h_{a}^{2}$, narrow-sense heritability; $h_{mp}^{2}$, heritability of progenies mean; *CV*_*gi*_%, additive genetic coefficient of variation; *CV*_*gp*_%, genotypic coefficient of variation among progenies; *CV*_*e*_%, experimental coefficient of variation; *CV*_*r*_, relative coefficient of variation; *Ac*_*prog*_, selective accuracy of progenies.

Genetic divergence estimation via Tocher’s method considering the Mahalanobis distance allowed the formation of fourteen different groups (Table 3). Approximately 86% of the progenies clustered as a part of group I, meaning that this group includes mostly similar genotypes. The most divergent group was Group XIV, with one progeny. However, groups IV, V and II; III; VI; VIII; and VII, had 9, 8, 5, 4 and 2 progenies, respectively, while groups IX, X, XI, XII and XII had with one progeny each, showing a wide diversity.

**Table 3:** Genetic divergence estimation of *E*. *camaldulensis* through Tocher’s method using Mahalanobis distance among 132 progenies in savanna biome from Mato Grosso, Brazil.

Groups	Progenies	Total
**I**	1, 3, 4, 6, 8, 9, 11, 12, 13, 14, 16, 19, 21, 22, 23, 24, 26, 27, 30, 31, 32, 33, 34, 37, 38, 39, 40, 41, 42, 43, 45, 46, 48, 49, 50, 51, 52, 53, 54, 55, 57, 59, 61, 63, 65, 67, 71, 73, 74, 75, 77, 79, 80, 84, 87, 88, 90, 91, 92, 93, 95, 96, 97, 98, 101, 102, 103, 105, 107, 110, 112, 113, 114, 115, 116, 117, 119, 120, 122, 123, 124, 125, 127, 129, 130, 72	86
**II**	7, 18, 56, 62, 70, 85, 89, 100	8
**III**	17, 29, 60, 94, 118	5
**IV**	5, 25, 28, 36, 69, 76, 82, 109, 121	9
**V**	15, 58, 64, 78, 83, 104, 106, 126	8
**VI**	2, 10, 86, 111	4
**VII**	47, 81	2
**VIII**	20, 99, 108, 131	4
**IX**	68	1
**X**	128	1
**XI**	66	1
**XII**	132	1
**XIII**	35	1
**XIV**	44	1

The clustering of genotypes based on the genetic divergence of 132 progenies of *E*. *camaldulensis* via Tocher’s method considering Euclidian distance formed eight distinct groups (Table 4).

**Table 4:** Genetic divergence estimation of *Eucalyptus camaldulensis* through Tocher’s method using Euclidian distance among 132 progenies in savanna biome from Mato Grosso, Brazil.

Groups	Progenies	Total
**I**	1, 2, 3, 4, 6, 7, 8, 9, 11, 12, 13, 14, 16, 21, 22, 23, 24, 27, 30, 31, 32, 33, 34, 37, 38, 39, 40, 41, 43, 45, 46, 47, 48, 49, 50, 51, 52, 56, 59, 61, 62, 70, 71, 74, 75, 78, 79, 80, 83, 84, 85, 90, 91, 93, 95, 96, 99, 101, 103, 105, 106, 107, 110, 111, 113, 114, 115, 116, 117, 122, 123, 124, 127, 129, 130, 72	76
**II**	10, 17, 26, 29, 36, 42, 53, 54, 55, 60, 63, 65, 66, 67, 68, 73, 77, 87, 88, 94, 97, 98, 102, 108, 112, 121, 125, 131, 132	29
**III**	25, 28, 76, 82, 109, 128	6
**IV**	15, 18, 19, 35, 57, 64, 81, 86, 89, 92, 100, 119, 120, 126	14
**V**	5, 69, 118	3
**VI**	44, 58	2
**VII**	104	1
**VIII**	20	1

## Discussion

Genetic parameter estimates of growth traits are essential for the breeder to identify superior genotypes. Following the concepts of Resende [13], the additive heritability ($h_{a}^{2}$) was moderate for DBH (0.16) and low for TH (0.08), CH (0.05), SF (0.07) and SR (0.12). These estimates agree with those found for other species and indicate that the progenies of *E*. *camaldulensis* are strong candidates for breeding programs (Table 2).

The mean heritability of the progenies ($h_{mp}^{2}$) (Table 2) was moderate for DBH (0.28), SR (0.30) and SF (0.16) and low for TH (0.13) and CH (0.09). The moderate heritability for DBH, SR and SF suggests a greater genetic divergence and could result in interesting morphological gains if used during parental selection.

The genotypic coefficient of variation (*CV*_*gi*_%) ranged from 3.32% for SR to 16.56% for DBH, similar to the results reported by Rocha et al. [20]. The genetic divergence estimates via Tocher’s method using the Mahalanobis distance allowed the identification of fourteen distinct groups (Table 3).

In terms of commercial wood species, the group number distribution in our study was very consistent with that reported by Silva et al. [6], who obtained 13 groups with one progeny each. These authors also used Tocher's method to evaluate the morphological traits in *Pinus caribaea* among different populations. Similarly, a study by Missio et al. [8] covering 119 progenies of *P*. *caribaea* also resulted in 14 distinct groups, six of them with only one progeny.

Breeding studies in wood species usually comprise a large number of progenies and in certain situations also assume that the offspring maintain a greater divergence and allow the breeder to select progenies considering the diversity within and between groups. Therefore, elucidating the minimum genetic divergence is crucial for ensuring the breeding sequence and helps to select the most divergent parents for crossings in order to provide a greater variability in future generations [21].

Thus, crossings by controlled pollination between the most divergent groups (I and XIV, IV and XIII, V and XII, and II and XI) are highly recommended to maintain the variability for subsequent selection cycles. These recommendations are consistent with those suggested by Martins et al. [21] for quantitative traits of *E*. *camaldulensis* and by Silva et al. [6] for *Pinus caribaea* var. *caribaea*.

When the Euclidian distance was considered, the clustering of 132 progenies resulted in eight distinct groups (Table 4). The most similar genotypes were clustered into group I (76 progenies) and the most divergent into Group VIII (one progeny). Noteworthy, groups II, IV, III and V had 29, 14, 6 and 3 progenies, respectively, while groups VI, VII and VIII had 2, 1 and 1 progenies, respectively. These results demonstrate the broad diversity and that the progenies that allocated within groups were less divergent than those that allocated between groups. The same model of distribution can been found in other studies with *E*. *camaldulensis* [21], *E*. *grandis* [3], and *Eucalyptus spp*. [22, 23] and for other forest species, such as *Dalbergia sissoo* [11] and *Ilex paraguariensis* [24].

Based on the results of Table 4, we recommend crossings between groups I and VI, II and IV, III and VII, and V and VIII. Crossings between I and II, III and IV, V and VI, and VII and VIII are not suitable because the parents show a low divergence.

Clustering using multivariate methods was very efficient in this study; however, as Vellini [22] also observed, these methods did not produce the same number of groups. The Mahalanobis distance seems to be more efficient for allocating the progenies into a larger number of groups and facilitates the identification of the most divergent groups to be used in a controlled breeding program.

The results also make it possible to establish specific crossings and strategies for improvement programs aiming at specific traits for different industries if the crossings prioritize individuals from different groups with greater dissimilarity and a consequently greater heterotic effect. Finally, it is important to consider that best crossings should consider not only the genetic divergence but also the superiority of genotypes for the desired characters [25].

## Conclusions

There is significant genetic variation among populations of *Eucalyptus camaldulensis* in the savanna region of Mato Grosso, Brazil. The approach using grouping methods was effective for the allocation of progenies into clusters by similarity. Tocher's method using the Mahalanobis distance seemed more efficient as a clustering technique for this species, because it allocated a larger number of groups and increased the possibility of crossings through controlled pollination between more divergent progenies. This information if important for use in breeding programs.

Future developments should include experiments at different locations comparing multiple groups to determine the environmental influences on plant performance. In addition, the use of molecular biology would note the occurrence of polymorphisms among progenies, helping to select promising genotypes for different industrial purposes.

## Supporting Information

S1 Appendix Data underlying to the manuscript. (DOCX)
