# Supplementary material for: Genetic Divergence in Eucalyptus camaldulensis Progenies in the Savanna Biome in Mato Grosso, Brazil
Source: PLoS One. 2016 Sep 28;11(9):e0163698. doi: 10.1371/journal.pone.0163698 (PMC5040395; doi:10.1371/journal.pone.0163698)
Supplement: S1 Appendix — (DOCX) [file pone.0163698.s001.docx]

**S1 Appendix. Data underlying to the manuscript.**

| Individual | Progeny | Replications | Plot | Tree | Diameter at breast height (DBH) (cm) | Total height (TH)  (m) | Commercial height (CH)  (m) | Stem form (SF) | Survival rate (SR) |
| --- | --- | --- | --- | --- | --- | --- | --- | --- | --- |
| 1 | 1 | 1 | 1 | 1 | 9.74 | 8.00 | 6.00 | 4 | 1 |
| 2 | 1 | 1 | 1 | 2 | 4.62 | 7.00 | 6.00 | 4 | 1 |
| 3 | 1 | 1 | 1 | 3 | 5.89 | 7.20 | 6.20 | 4 | 1 |
| 4 | 2 | 1 | 2 | 1 | 5.03 | 6.50 | 5.50 | 4 | 1 |
| 5 | 2 | 1 | 2 | 2 | 5.19 | 6.00 | 4.00 | 5 | 1 |
| 6 | 2 | 1 | 2 | 3 | 5.06 | 7.00 | 6.00 | 5 | 1 |
| 7 | 3 | 1 | 3 | 1 | 10.82 | 11.00 | 7.00 | 5 | 1 |
| 8 | 3 | 1 | 3 | 2 | 11.14 | 10.50 | 9.50 | 5 | 1 |
| 9 | 3 | 1 | 3 | 3 | 5.73 | 9.50 | 8.00 | 4 | 1 |
| 10 | 4 | 1 | 4 | 1 | 6.53 | 9.00 | 8.00 | 4 | 1 |
| 11 | 4 | 1 | 4 | 2 | 0.00 | 0.00 | 0.00 | 0 | 0 |
| 12 | 4 | 1 | 4 | 3 | 7.64 | 10.00 | 7.00 | 4 | 1 |
| 13 | 5 | 1 | 5 | 1 | 5.73 | 6.00 | 5.00 | 4 | 1 |
| 14 | 5 | 1 | 5 | 2 | 1.59 | 2.30 | 1.60 | 4 | 1 |
| 15 | 5 | 1 | 5 | 3 | 0.00 | 0.00 | 0.00 | 0 | 0 |
| 16 | 6 | 1 | 6 | 1 | 6.18 | 5.00 | 3.80 | 5 | 1 |
| 17 | 6 | 1 | 6 | 2 | 4.49 | 4.00 | 3.00 | 5 | 1 |
| 18 | 6 | 1 | 6 | 3 | 3.69 | 5.00 | 4.00 | 5 | 1 |
| 19 | 7 | 1 | 7 | 1 | 6.27 | 6.30 | 2.60 | 5 | 1 |
| 20 | 7 | 1 | 7 | 2 | 4.65 | 6.00 | 5.20 | 5 | 1 |
| 21 | 7 | 1 | 7 | 3 | 7.48 | 6.50 | 5.00 | 5 | 1 |
| 22 | 8 | 1 | 8 | 1 | 2.23 | 2.00 | 1.00 | 4 | 1 |
| 23 | 8 | 1 | 8 | 2 | 0.00 | 0.00 | 0.00 | 0 | 0 |
| 24 | 8 | 1 | 8 | 3 | 3.57 | 2.00 | 1.50 | 5 | 1 |
| 25 | 9 | 1 | 9 | 1 | 2.39 | 4.00 | 3.00 | 5 | 1 |
| 26 | 9 | 1 | 9 | 2 | 6.11 | 6.50 | 3.00 | 4 | 1 |
| 27 | 9 | 1 | 9 | 3 | 4.17 | 3.20 | 2.50 | 4 | 1 |
| 28 | 10 | 1 | 10 | 1 | 6.53 | 4.50 | 3.80 | 5 | 1 |
| 29 | 10 | 1 | 10 | 2 | 6.37 | 4.60 | 3.50 | 4 | 1 |
| 30 | 10 | 1 | 10 | 3 | 6.65 | 5.00 | 4.20 | 5 | 1 |
| 31 | 11 | 1 | 11 | 1 | 6.84 | 7.00 | 4.00 | 4 | 1 |
| 32 | 11 | 1 | 11 | 2 | 7.64 | 8.00 | 7.00 | 4 | 1 |
| 33 | 11 | 1 | 11 | 3 | 4.93 | 7.00 | 6.00 | 4 | 1 |
| 34 | 12 | 1 | 12 | 1 | 6.18 | 5.00 | 3.80 | 4 | 1 |
| 35 | 12 | 1 | 12 | 2 | 6.05 | 6.00 | 5.00 | 4 | 1 |
| 36 | 12 | 1 | 12 | 3 | 6.05 | 6.50 | 5.50 | 4 | 1 |
| 37 | 13 | 1 | 13 | 1 | 5.35 | 4.50 | 2.20 | 5 | 1 |
| 38 | 13 | 1 | 13 | 2 | 4.14 | 4.00 | 3.50 | 4 | 1 |
| 39 | 13 | 1 | 13 | 3 | 3.72 | 3.60 | 2.00 | 4 | 1 |
| 40 | 14 | 1 | 14 | 1 | 4.62 | 6.00 | 5.00 | 4 | 1 |
| 41 | 14 | 1 | 14 | 2 | 4.42 | 6.50 | 5.50 | 5 | 1 |
| 42 | 14 | 1 | 14 | 3 | 8.28 | 7.00 | 6.00 | 5 | 1 |
| 43 | 15 | 1 | 15 | 1 | 6.56 | 10.00 | 9.00 | 5 | 1 |
| 44 | 15 | 1 | 15 | 2 | 15.28 | 13.00 | 11.00 | 5 | 1 |
| 45 | 15 | 1 | 15 | 3 | 11.30 | 11.00 | 9.00 | 5 | 1 |
| 46 | 16 | 1 | 16 | 1 | 5.25 | 6.50 | 3.80 | 5 | 1 |
| 47 | 16 | 1 | 16 | 2 | 6.43 | 4.50 | 3.80 | 5 | 1 |
| 48 | 16 | 1 | 16 | 3 | 7.54 | 6.50 | 3.80 | 5 | 1 |
| 49 | 17 | 1 | 17 | 1 | 4.77 | 6.00 | 4.00 | 4 | 1 |
| 50 | 17 | 1 | 17 | 2 | 9.17 | 8.00 | 2.00 | 4 | 1 |
| 51 | 17 | 1 | 17 | 3 | 6.91 | 7.50 | 5.50 | 4 | 1 |
| 52 | 18 | 1 | 18 | 1 | 6.27 | 9.50 | 8.50 | 5 | 1 |
| 53 | 18 | 1 | 18 | 2 | 0.00 | 0.00 | 0.00 | 0 | 0 |
| 54 | 18 | 1 | 18 | 3 | 3.50 | 8.50 | 5.50 | 5 | 1 |
| 55 | 19 | 1 | 19 | 1 | 9.29 | 6.50 | 5.80 | 5 | 1 |
| 56 | 19 | 1 | 19 | 2 | 11.78 | 10.00 | 7.00 | 5 | 1 |
| 57 | 19 | 1 | 19 | 3 | 12.41 | 10.50 | 8.50 | 5 | 1 |
| 58 | 20 | 1 | 20 | 1 | 4.93 | 4.00 | 3.50 | 5 | 1 |
| 59 | 20 | 1 | 20 | 2 | 3.50 | 3.50 | 2.40 | 5 | 1 |
| 60 | 20 | 1 | 20 | 3 | 3.25 | 3.00 | 2.50 | 5 | 1 |
| 61 | 21 | 1 | 21 | 1 | 13.69 | 12.00 | 7.00 | 4 | 1 |
| 62 | 21 | 1 | 21 | 2 | 5.89 | 6.00 | 5.00 | 4 | 1 |
| 63 | 21 | 1 | 21 | 3 | 5.89 | 6.50 | 6.00 | 4 | 1 |
| 64 | 22 | 1 | 22 | 1 | 3.18 | 3.20 | 2.80 | 5 | 1 |
| 65 | 22 | 1 | 22 | 2 | 3.82 | 4.00 | 3.20 | 4 | 1 |
| 66 | 22 | 1 | 22 | 3 | 1.27 | 2.50 | 2.30 | 4 | 1 |
| 67 | 23 | 1 | 23 | 1 | 9.26 | 9.10 | 6.00 | 5 | 1 |
| 68 | 23 | 1 | 23 | 2 | 8.02 | 7.00 | 5.80 | 5 | 1 |
| 69 | 23 | 1 | 23 | 3 | 8.02 | 5.50 | 4.30 | 5 | 1 |
| 70 | 24 | 1 | 24 | 1 | 3.82 | 5.00 | 4.00 | 4 | 1 |
| 71 | 24 | 1 | 24 | 2 | 12.25 | 8.00 | 7.00 | 5 | 1 |
| 72 | 24 | 1 | 24 | 3 | 5.54 | 7.00 | 6.00 | 4 | 1 |
| 73 | 25 | 1 | 25 | 1 | 4.97 | 6.00 | 5.00 | 4 | 1 |
| 74 | 25 | 1 | 25 | 2 | 4.52 | 7.00 | 6.00 | 4 | 1 |
| 75 | 25 | 1 | 25 | 3 | 2.23 | 4.00 | 2.50 | 5 | 1 |
| 76 | 26 | 1 | 26 | 1 | 6.59 | 8.00 | 7.30 | 5 | 1 |
| 77 | 26 | 1 | 26 | 2 | 6.68 | 8.20 | 7.80 | 5 | 1 |
| 78 | 26 | 1 | 26 | 3 | 5.73 | 7.50 | 6.50 | 5 | 1 |
| 79 | 27 | 1 | 27 | 1 | 9.64 | 8.00 | 5.00 | 4 | 1 |
| 80 | 27 | 1 | 27 | 2 | 3.79 | 6.00 | 3.00 | 4 | 1 |
| 81 | 27 | 1 | 27 | 3 | 4.81 | 5.70 | 3.50 | 5 | 1 |
| 82 | 28 | 1 | 28 | 1 | 8.91 | 9.00 | 7.00 | 5 | 1 |
| 83 | 28 | 1 | 28 | 2 | 11.84 | 13.00 | 11.50 | 5 | 1 |
| 84 | 28 | 1 | 28 | 3 | 3.85 | 9.50 | 8.00 | 4 | 1 |
| 85 | 29 | 1 | 29 | 1 | 1.27 | 4.00 | 1.40 | 4 | 1 |
| 86 | 29 | 1 | 29 | 2 | 2.77 | 8.70 | 2.60 | 5 | 1 |
| 87 | 29 | 1 | 29 | 3 | 2.23 | 7.00 | 1.80 | 4 | 1 |
| 88 | 30 | 1 | 30 | 1 | 7.32 | 7.50 | 5.50 | 4 | 1 |
| 89 | 30 | 1 | 30 | 2 | 4.30 | 6.00 | 5.00 | 4 | 1 |
| 90 | 30 | 1 | 30 | 3 | 7.16 | 7.00 | 6.00 | 4 | 1 |
| 91 | 31 | 1 | 31 | 1 | 2.83 | 8.00 | 5.00 | 5 | 1 |
| 92 | 31 | 1 | 31 | 2 | 6.33 | 7.00 | 6.00 | 4 | 1 |
| 93 | 31 | 1 | 31 | 3 | 4.42 | 7.50 | 6.50 | 4 | 1 |
| 94 | 32 | 1 | 32 | 1 | 0.00 | 0.00 | 0.00 | 0 | 0 |
| 95 | 32 | 1 | 32 | 2 | 3.92 | 4.70 | 4.00 | 5 | 1 |
| 96 | 32 | 1 | 32 | 3 | 4.68 | 5.50 | 3.80 | 5 | 1 |
| 97 | 33 | 1 | 33 | 1 | 5.47 | 6.00 | 4.00 | 5 | 1 |
| 98 | 33 | 1 | 33 | 2 | 7.19 | 7.00 | 5.50 | 5 | 1 |
| 99 | 33 | 1 | 33 | 3 | 5.79 | 6.00 | 3.50 | 5 | 1 |
| 100 | 34 | 1 | 34 | 1 | 0.00 | 0.00 | 0.00 | 0 | 0 |
| 101 | 34 | 1 | 34 | 2 | 3.18 | 3.00 | 2.50 | 4 | 1 |
| 102 | 34 | 1 | 34 | 3 | 10.60 | 8.00 | 5.00 | 5 | 1 |
| 103 | 35 | 1 | 35 | 1 | 4.14 | 8.10 | 7.00 | 4 | 1 |
| 104 | 35 | 1 | 35 | 2 | 7.96 | 8.00 | 7.00 | 5 | 1 |
| 105 | 35 | 1 | 35 | 3 | 6.78 | 9.00 | 7.00 | 5 | 1 |
| 106 | 36 | 1 | 36 | 1 | 3.02 | 3.50 | 2.60 | 4 | 1 |
| 107 | 36 | 1 | 36 | 2 | 4.68 | 5.30 | 4.80 | 5 | 1 |
| 108 | 36 | 1 | 36 | 3 | 2.10 | 3.00 | 2.50 | 4 | 1 |
| 109 | 37 | 1 | 37 | 1 | 4.17 | 4.80 | 4.50 | 4 | 1 |
| 110 | 37 | 1 | 37 | 2 | 3.25 | 4.20 | 3.80 | 3 | 1 |
| 111 | 37 | 1 | 37 | 3 | 5.28 | 5.50 | 3.80 | 4 | 1 |
| 112 | 38 | 1 | 38 | 1 | 10.50 | 9.00 | 7.00 | 4 | 1 |
| 113 | 38 | 1 | 38 | 2 | 11.78 | 10.00 | 8.00 | 4 | 1 |
| 114 | 38 | 1 | 38 | 3 | 11.14 | 11.00 | 9.00 | 4 | 1 |
| 115 | 39 | 1 | 39 | 1 | 6.88 | 6.50 | 5.00 | 5 | 1 |
| 116 | 39 | 1 | 39 | 2 | 3.85 | 3.80 | 2.60 | 5 | 1 |
| 117 | 39 | 1 | 39 | 3 | 4.93 | 5.80 | 5.50 | 4 | 1 |
| 118 | 40 | 1 | 40 | 1 | 4.81 | 5.10 | 4.50 | 5 | 1 |
| 119 | 40 | 1 | 40 | 2 | 6.02 | 4.80 | 3.20 | 5 | 1 |
| 120 | 40 | 1 | 40 | 3 | 5.38 | 6.00 | 4.00 | 5 | 1 |
| 121 | 41 | 1 | 41 | 1 | 5.60 | 5.00 | 3.00 | 4 | 1 |
| 122 | 41 | 1 | 41 | 2 | 2.16 | 3.00 | 1.80 | 4 | 1 |
| 123 | 41 | 1 | 41 | 3 | 3.50 | 4.50 | 3.00 | 5 | 1 |
| 124 | 42 | 1 | 42 | 1 | 5.89 | 6.00 | 2.70 | 4 | 1 |
| 125 | 42 | 1 | 42 | 2 | 4.77 | 5.80 | 4.80 | 4 | 1 |
| 126 | 42 | 1 | 42 | 3 | 4.65 | 6.00 | 5.00 | 5 | 1 |
| 127 | 43 | 1 | 43 | 1 | 7.29 | 8.50 | 8.00 | 4 | 1 |
| 128 | 43 | 1 | 43 | 2 | 10.22 | 11.00 | 9.00 | 5 | 1 |
| 129 | 43 | 1 | 43 | 3 | 11.33 | 10.00 | 9.00 | 5 | 1 |
| 130 | 44 | 1 | 44 | 1 | 1.91 | 3.50 | 2.50 | 4 | 1 |
| 131 | 44 | 1 | 44 | 2 | 6.37 | 8.00 | 7.50 | 4 | 1 |
| 132 | 44 | 1 | 44 | 3 | 10.35 | 9.00 | 7.00 | 4 | 1 |
| 133 | 45 | 1 | 45 | 1 | 7.19 | 6.50 | 2.60 | 4 | 1 |
| 134 | 45 | 1 | 45 | 2 | 3.53 | 3.20 | 2.50 | 4 | 1 |
| 135 | 45 | 1 | 45 | 3 | 4.20 | 3.00 | 2.50 | 4 | 1 |
| 136 | 46 | 1 | 46 | 1 | 0.00 | 0.00 | 0.00 | 0 | 0 |
| 137 | 46 | 1 | 46 | 2 | 4.77 | 8.00 | 2.50 | 4 | 1 |
| 138 | 46 | 1 | 46 | 3 | 4.77 | 7.00 | 6.00 | 4 | 1 |
| 139 | 47 | 1 | 47 | 1 | 10.52 | 10.50 | 8.50 | 4 | 1 |
| 140 | 47 | 1 | 47 | 2 | 9.55 | 11.00 | 9.00 | 5 | 1 |
| 141 | 47 | 1 | 47 | 3 | 14.32 | 12.00 | 10.00 | 4 | 1 |
| 142 | 48 | 1 | 48 | 1 | 2.16 | 3.20 | 2.70 | 4 | 1 |
| 143 | 48 | 1 | 48 | 2 | 1.94 | 4.00 | 3.50 | 5 | 1 |
| 144 | 48 | 1 | 48 | 3 | 4.87 | 7.00 | 6.00 | 4 | 1 |
| 145 | 49 | 1 | 49 | 1 | 0.00 | 0.00 | 0.00 | 0 | 0 |
| 146 | 49 | 1 | 49 | 2 | 7.51 | 9.50 | 7.50 | 4 | 1 |
| 147 | 49 | 1 | 49 | 3 | 4.68 | 6.00 | 5.00 | 4 | 1 |
| 148 | 50 | 1 | 50 | 1 | 13.21 | 9.50 | 7.50 | 4 | 1 |
| 149 | 50 | 1 | 50 | 2 | 10.35 | 9.50 | 7.00 | 4 | 1 |
| 150 | 50 | 1 | 50 | 3 | 2.55 | 3.00 | 3.50 | 4 | 1 |
| 151 | 51 | 1 | 51 | 1 | 8.79 | 8.00 | 7.00 | 5 | 1 |
| 152 | 51 | 1 | 51 | 2 | 3.60 | 5.50 | 4.00 | 4 | 1 |
| 153 | 51 | 1 | 51 | 3 | 6.78 | 7.00 | 6.00 | 5 | 1 |
| 154 | 52 | 1 | 52 | 1 | 9.87 | 11.00 | 8.00 | 5 | 1 |
| 155 | 52 | 1 | 52 | 2 | 10.47 | 9.50 | 6.00 | 5 | 1 |
| 156 | 52 | 1 | 52 | 3 | 7.32 | 7.50 | 6.50 | 5 | 1 |
| 157 | 53 | 1 | 53 | 1 | 9.87 | 6.00 | 4.80 | 5 | 1 |
| 158 | 53 | 1 | 53 | 2 | 6.62 | 5.50 | 4.50 | 5 | 1 |
| 159 | 53 | 1 | 53 | 3 | 5.89 | 5.50 | 4.80 | 5 | 1 |
| 160 | 54 | 1 | 54 | 1 | 6.78 | 5.50 | 4.80 | 5 | 1 |
| 161 | 54 | 1 | 54 | 2 | 4.46 | 4.80 | 4.00 | 5 | 1 |
| 162 | 54 | 1 | 54 | 3 | 5.41 | 6.00 | 5.20 | 5 | 1 |
| 163 | 55 | 1 | 55 | 1 | 5.22 | 5.50 | 4.00 | 5 | 1 |
| 164 | 55 | 1 | 55 | 2 | 3.18 | 3.50 | 2.60 | 3 | 1 |
| 165 | 55 | 1 | 55 | 3 | 3.06 | 3.10 | 1.50 | 4 | 1 |
| 166 | 56 | 1 | 56 | 1 | 4.49 | 7.00 | 4.00 | 5 | 1 |
| 167 | 56 | 1 | 56 | 2 | 4.55 | 6.00 | 4.00 | 4 | 1 |
| 168 | 56 | 1 | 56 | 3 | 5.60 | 5.50 | 4.00 | 4 | 1 |
| 169 | 57 | 1 | 57 | 1 | 8.59 | 8.00 | 6.30 | 5 | 1 |
| 170 | 57 | 1 | 57 | 2 | 9.84 | 9.50 | 6.00 | 5 | 1 |
| 171 | 57 | 1 | 57 | 3 | 8.09 | 8.00 | 7.00 | 5 | 1 |
| 172 | 58 | 1 | 58 | 1 | 5.41 | 5.80 | 4.50 | 4 | 1 |
| 173 | 58 | 1 | 58 | 2 | 6.56 | 7.00 | 5.20 | 4 | 1 |
| 174 | 58 | 1 | 58 | 3 | 7.29 | 7.50 | 6.10 | 4 | 1 |
| 175 | 59 | 1 | 59 | 1 | 4.93 | 5.50 | 3.50 | 4 | 1 |
| 176 | 59 | 1 | 59 | 2 | 9.55 | 7.20 | 6.00 | 4 | 1 |
| 177 | 59 | 1 | 59 | 3 | 9.55 | 7.80 | 6.50 | 4 | 1 |
| 178 | 60 | 1 | 60 | 1 | 8.50 | 7.50 | 5.50 | 5 | 1 |
| 179 | 60 | 1 | 60 | 2 | 6.11 | 17.20 | 5.80 | 5 | 1 |
| 180 | 60 | 1 | 60 | 3 | 5.92 | 5.50 | 3.20 | 4 | 1 |
| 181 | 61 | 1 | 61 | 1 | 2.83 | 8.00 | 6.00 | 4 | 1 |
| 182 | 61 | 1 | 61 | 2 | 7.00 | 7.50 | 6.60 | 5 | 1 |
| 183 | 61 | 1 | 61 | 3 | 7.16 | 5.00 | 4.00 | 4 | 1 |
| 184 | 62 | 1 | 62 | 1 | 7.73 | 9.30 | 8.30 | 5 | 1 |
| 185 | 62 | 1 | 62 | 2 | 6.41 | 11.00 | 2.60 | 4 | 1 |
| 186 | 62 | 1 | 62 | 3 | 7.67 | 9.80 | 8.80 | 5 | 1 |
| 187 | 63 | 1 | 63 | 1 | 4.17 | 3.50 | 2.00 | 4 | 1 |
| 188 | 63 | 1 | 63 | 2 | 2.93 | 3.00 | 2.00 | 5 | 1 |
| 189 | 63 | 1 | 63 | 3 | 3.79 | 4.00 | 2.50 | 5 | 1 |
| 190 | 64 | 1 | 64 | 1 | 5.46 | 11.50 | 4.00 | 4 | 1 |
| 191 | 64 | 1 | 64 | 2 | 7.80 | 12.00 | 1.50 | 4 | 1 |
| 192 | 64 | 1 | 64 | 3 | 9.68 | 10.50 | 6.00 | 5 | 1 |
| 193 | 65 | 1 | 65 | 1 | 4.46 | 3.20 | 2.50 | 4 | 1 |
| 194 | 65 | 1 | 65 | 2 | 4.33 | 3.00 | 2.60 | 4 | 1 |
| 195 | 65 | 1 | 65 | 3 | 4.93 | 4.50 | 4.00 | 5 | 1 |
| 196 | 66 | 1 | 66 | 1 | 4.36 | 6.50 | 5.50 | 5 | 1 |
| 197 | 66 | 1 | 66 | 2 | 7.26 | 9.50 | 9.00 | 5 | 1 |
| 198 | 66 | 1 | 66 | 3 | 7.58 | 10.00 | 9.00 | 5 | 1 |
| 199 | 67 | 1 | 67 | 1 | 2.07 | 3.00 | 2.50 | 5 | 1 |
| 200 | 67 | 1 | 67 | 2 | 0.00 | 0.00 | 0.00 | 0 | 0 |
| 201 | 67 | 1 | 67 | 3 | 4.27 | 6.00 | 5.00 | 5 | 1 |
| 202 | 68 | 1 | 68 | 1 | 5.86 | 5.20 | 2.50 | 5 | 1 |
| 203 | 68 | 1 | 68 | 2 | 4.74 | 3.50 | 3.00 | 5 | 1 |
| 204 | 68 | 1 | 68 | 3 | 6.27 | 5.50 | 2.60 | 5 | 1 |
| 205 | 69 | 1 | 69 | 1 | 0.00 | 0.00 | 0.00 | 0 | 0 |
| 206 | 69 | 1 | 69 | 2 | 3.92 | 6.00 | 5.50 | 5 | 1 |
| 207 | 69 | 1 | 69 | 3 | 1.75 | 2.60 | 1.30 | 4 | 1 |
| 208 | 70 | 1 | 70 | 1 | 6.05 | 9.00 | 8.00 | 5 | 1 |
| 209 | 70 | 1 | 70 | 2 | 2.55 | 5.50 | 5.00 | 5 | 1 |
| 210 | 70 | 1 | 70 | 3 | 2.86 | 6.50 | 6.00 | 5 | 1 |
| 211 | 71 | 1 | 71 | 1 | 5.51 | 7.00 | 5.00 | 5 | 1 |
| 212 | 71 | 1 | 71 | 2 | 7.89 | 10.00 | 7.50 | 5 | 1 |
| 213 | 71 | 1 | 71 | 3 | 3.06 | 4.00 | 3.60 | 4 | 1 |
| 214 | 72 | 1 | 72 | 1 | 0.00 | 0.00 | 0.00 | 0 | 0 |
| 215 | 72 | 1 | 72 | 2 | 0.00 | 0.00 | 0.00 | 0 | 0 |
| 216 | 72 | 1 | 72 | 3 | 0.00 | 0.00 | 0.00 | 0 | 0 |
| 217 | 73 | 1 | 73 | 1 | 4.01 | 6.00 | 5.00 | 5 | 1 |
| 218 | 73 | 1 | 73 | 2 | 3.82 | 5.00 | 4.00 | 4 | 1 |
| 219 | 73 | 1 | 73 | 3 | 7.26 | 8.00 | 7.00 | 4 | 1 |
| 220 | 74 | 1 | 74 | 1 | 3.98 | 6.00 | 5.50 | 4 | 1 |
| 221 | 74 | 1 | 74 | 2 | 5.38 | 7.50 | 6.50 | 4 | 1 |
| 222 | 74 | 1 | 74 | 3 | 4.30 | 8.00 | 7.00 | 5 | 1 |
| 223 | 75 | 1 | 75 | 1 | 11.78 | 9.00 | 7.00 | 4 | 1 |
| 224 | 75 | 1 | 75 | 2 | 8.59 | 8.00 | 6.00 | 4 | 1 |
| 225 | 75 | 1 | 75 | 3 | 9.20 | 7.50 | 6.40 | 4 | 1 |
| 226 | 76 | 1 | 76 | 1 | 4.77 | 6.00 | 4.00 | 4 | 1 |
| 227 | 76 | 1 | 76 | 2 | 5.73 | 7.00 | 6.00 | 4 | 1 |
| 228 | 76 | 1 | 76 | 3 | 5.57 | 5.50 | 4.80 | 4 | 1 |
| 229 | 77 | 1 | 77 | 1 | 7.35 | 9.00 | 7.00 | 5 | 1 |
| 230 | 77 | 1 | 77 | 2 | 0.00 | 0.00 | 0.00 | 0 | 0 |
| 231 | 77 | 1 | 77 | 3 | 3.28 | 6.00 | 5.00 | 4 | 1 |
| 232 | 78 | 1 | 78 | 1 | 8.24 | 9.00 | 7.00 | 4 | 1 |
| 233 | 78 | 1 | 78 | 2 | 6.68 | 6.00 | 5.50 | 4 | 1 |
| 234 | 78 | 1 | 78 | 3 | 9.55 | 8.20 | 7.50 | 4 | 1 |
| 235 | 79 | 1 | 79 | 1 | 3.50 | 4.00 | 1.50 | 4 | 1 |
| 236 | 79 | 1 | 79 | 2 | 8.75 | 8.00 | 6.00 | 4 | 1 |
| 237 | 79 | 1 | 79 | 3 | 5.41 | 6.50 | 5.50 | 4 | 1 |
| 238 | 80 | 1 | 80 | 1 | 5.57 | 7.00 | 6.50 | 5 | 1 |
| 239 | 80 | 1 | 80 | 2 | 9.55 | 8.00 | 7.50 | 5 | 1 |
| 240 | 80 | 1 | 80 | 3 | 8.50 | 7.00 | 6.00 | 5 | 1 |
| 241 | 81 | 1 | 81 | 1 | 0.00 | 0.00 | 0.00 | 0 | 0 |
| 242 | 81 | 1 | 81 | 2 | 5.16 | 7.50 | 6.50 | 4 | 1 |
| 243 | 81 | 1 | 81 | 3 | 16.90 | 11.00 | 9.00 | 5 | 1 |
| 244 | 82 | 1 | 82 | 1 | 5.09 | 8.00 | 7.00 | 4 | 1 |
| 245 | 82 | 1 | 82 | 2 | 12.41 | 11.50 | 9.50 | 4 | 1 |
| 246 | 82 | 1 | 82 | 3 | 2.71 | 4.00 | 3.00 | 4 | 1 |
| 247 | 83 | 1 | 83 | 1 | 13.53 | 11.50 | 6.00 | 4 | 1 |
| 248 | 83 | 1 | 83 | 2 | 12.76 | 11.00 | 7.00 | 4 | 1 |
| 249 | 83 | 1 | 83 | 3 | 0.00 | 0.00 | 0.00 | 0 | 0 |
| 250 | 84 | 1 | 84 | 1 | 9.96 | 9.80 | 7.80 | 4 | 1 |
| 251 | 84 | 1 | 84 | 2 | 9.45 | 9.00 | 6.00 | 5 | 1 |
| 252 | 84 | 1 | 84 | 3 | 4.77 | 6.00 | 5.00 | 4 | 1 |
| 253 | 85 | 1 | 85 | 1 | 1.69 | 2.50 | 1.50 | 5 | 1 |
| 254 | 85 | 1 | 85 | 2 | 16.65 | 14.00 | 10.00 | 5 | 1 |
| 255 | 85 | 1 | 85 | 3 | 12.51 | 11.00 | 9.00 | 4 | 1 |
| 256 | 86 | 1 | 86 | 1 | 4.20 | 6.00 | 5.00 | 5 | 1 |
| 257 | 86 | 1 | 86 | 2 | 11.68 | 12.00 | 8.00 | 5 | 1 |
| 258 | 86 | 1 | 86 | 3 | 9.45 | 11.00 | 8.00 | 4 | 1 |
| 259 | 87 | 1 | 87 | 1 | 2.86 | 3.50 | 2.60 | 4 | 1 |
| 260 | 87 | 1 | 87 | 2 | 2.86 | 3.00 | 2.50 | 4 | 1 |
| 261 | 87 | 1 | 87 | 3 | 5.41 | 6.00 | 5.00 | 5 | 1 |
| 262 | 88 | 1 | 88 | 1 | 7.32 | 9.00 | 8.00 | 5 | 1 |
| 263 | 88 | 1 | 88 | 2 | 5.63 | 9.00 | 7.00 | 3 | 1 |
| 264 | 88 | 1 | 88 | 3 | 0.00 | 0.00 | 0.00 | 0 | 0 |
| 265 | 89 | 1 | 89 | 1 | 14.10 | 10.50 | 8.50 | 4 | 1 |
| 266 | 89 | 1 | 89 | 2 | 10.19 | 8.50 | 7.80 | 4 | 1 |
| 267 | 89 | 1 | 89 | 3 | 9.17 | 9.00 | 7.80 | 4 | 1 |
| 268 | 90 | 1 | 90 | 1 | 7.48 | 8.00 | 6.00 | 4 | 1 |
| 269 | 90 | 1 | 90 | 2 | 0.00 | 0.00 | 0.00 | 0 | 0 |
| 270 | 90 | 1 | 90 | 3 | 9.33 | 10.00 | 5.00 | 5 | 1 |
| 271 | 91 | 1 | 91 | 1 | 9.99 | 10.50 | 8.00 | 5 | 1 |
| 272 | 91 | 1 | 91 | 2 | 6.14 | 9.00 | 8.00 | 4 | 1 |
| 273 | 91 | 1 | 91 | 3 | 4.14 | 6.00 | 5.00 | 5 | 1 |
| 274 | 92 | 1 | 92 | 1 | 5.89 | 8.60 | 8.00 | 4 | 1 |
| 275 | 92 | 1 | 92 | 2 | 12.41 | 11.00 | 9.00 | 5 | 1 |
| 276 | 92 | 1 | 92 | 3 | 9.49 | 9.20 | 8.00 | 4 | 1 |
| 277 | 93 | 1 | 93 | 1 | 1.27 | 2.00 | 1.50 | 5 | 1 |
| 278 | 93 | 1 | 93 | 2 | 1.99 | 6.00 | 5.00 | 5 | 1 |
| 279 | 93 | 1 | 93 | 3 | 1.27 | 6.50 | 5.50 | 4 | 1 |
| 280 | 94 | 1 | 94 | 1 | 5.98 | 9.50 | 2.50 | 5 | 1 |
| 281 | 94 | 1 | 94 | 2 | 7.48 | 8.00 | 7.00 | 5 | 1 |
| 282 | 94 | 1 | 94 | 3 | 2.55 | 3.20 | 1.00 | 4 | 1 |
| 283 | 95 | 1 | 95 | 1 | 1.91 | 2.50 | 2.00 | 5 | 1 |
| 284 | 95 | 1 | 95 | 2 | 3.60 | 4.00 | 3.50 | 5 | 1 |
| 285 | 95 | 1 | 95 | 3 | 8.31 | 9.50 | 7.50 | 4 | 1 |
| 286 | 96 | 1 | 96 | 1 | 13.02 | 11.00 | 10.20 | 4 | 1 |
| 287 | 96 | 1 | 96 | 2 | 11.46 | 10.50 | 9.00 | 5 | 1 |
| 288 | 96 | 1 | 96 | 3 | 9.39 | 9.60 | 8.00 | 5 | 1 |
| 289 | 97 | 1 | 97 | 1 | 0.00 | 0.00 | 0.00 | 0 | 0 |
| 290 | 97 | 1 | 97 | 2 | 2.13 | 2.60 | 1.30 | 4 | 1 |
| 291 | 97 | 1 | 97 | 3 | 0.00 | 0.00 | 0.00 | 0 | 0 |
| 292 | 98 | 1 | 98 | 1 | 5.25 | 3.50 | 2.40 | 4 | 1 |
| 293 | 98 | 1 | 98 | 2 | 2.93 | 5.20 | 4.00 | 5 | 1 |
| 294 | 98 | 1 | 98 | 3 | 3.57 | 4.50 | 3.80 | 5 | 1 |
| 295 | 99 | 1 | 99 | 1 | 13.59 | 11.00 | 9.00 | 5 | 1 |
| 296 | 99 | 1 | 99 | 2 | 8.59 | 10.00 | 9.00 | 5 | 1 |
| 297 | 99 | 1 | 99 | 3 | 18.46 | 13.00 | 11.00 | 5 | 1 |
| 298 | 100 | 1 | 100 | 1 | 13.37 | 11.50 | 7.00 | 5 | 1 |
| 299 | 100 | 1 | 100 | 2 | 9.33 | 11.00 | 9.00 | 4 | 1 |
| 300 | 100 | 1 | 100 | 3 | 6.84 | 9.00 | 8.00 | 5 | 1 |
| 301 | 101 | 1 | 101 | 1 | 7.93 | 9.20 | 8.20 | 4 | 1 |
| 302 | 101 | 1 | 101 | 2 | 9.20 | 9.80 | 8.80 | 5 | 1 |
| 303 | 101 | 1 | 101 | 3 | 7.48 | 9.00 | 8.00 | 5 | 1 |
| 304 | 102 | 1 | 102 | 1 | 0.00 | 0.00 | 0.00 | 0 | 0 |
| 305 | 102 | 1 | 102 | 2 | 3.02 | 4.00 | 3.00 | 4 | 1 |
| 306 | 102 | 1 | 102 | 3 | 2.55 | 3.00 | 2.60 | 4 | 1 |
| 307 | 103 | 1 | 103 | 1 | 5.28 | 7.50 | 6.50 | 4 | 1 |
| 308 | 103 | 1 | 103 | 2 | 3.92 | 5.50 | 4.50 | 2 | 1 |
| 309 | 103 | 1 | 103 | 3 | 13.62 | 13.00 | 10.00 | 5 | 1 |
| 310 | 104 | 1 | 104 | 1 | 2.07 | 3.00 | 2.00 | 4 | 1 |
| 311 | 104 | 1 | 104 | 2 | 8.75 | 10.00 | 9.00 | 5 | 1 |
| 312 | 104 | 1 | 104 | 3 | 12.83 | 11.00 | 8.50 | 5 | 1 |
| 313 | 105 | 1 | 105 | 1 | 10.95 | 10.50 | 7.00 | 5 | 1 |
| 314 | 105 | 1 | 105 | 2 | 6.68 | 8.00 | 6.00 | 5 | 1 |
| 315 | 105 | 1 | 105 | 3 | 10.85 | 11.00 | 9.00 | 5 | 1 |
| 316 | 106 | 1 | 106 | 1 | 7.13 | 8.50 | 6.50 | 4 | 1 |
| 317 | 106 | 1 | 106 | 2 | 11.46 | 9.50 | 7.50 | 3 | 1 |
| 318 | 106 | 1 | 106 | 3 | 2.55 | 4.00 | 2.60 | 5 | 1 |
| 319 | 107 | 1 | 107 | 1 | 9.71 | 9.00 | 8.00 | 5 | 1 |
| 320 | 107 | 1 | 107 | 2 | 8.31 | 10.00 | 8.00 | 4 | 1 |
| 321 | 107 | 1 | 107 | 3 | 10.60 | 10.20 | 8.50 | 5 | 1 |
| 322 | 108 | 1 | 108 | 1 | 7.29 | 7.50 | 6.20 | 5 | 1 |
| 323 | 108 | 1 | 108 | 2 | 10.25 | 10.00 | 7.50 | 5 | 1 |
| 324 | 108 | 1 | 108 | 3 | 11.55 | 9.00 | 7.80 | 5 | 1 |
| 325 | 109 | 1 | 109 | 1 | 7.89 | 8.00 | 6.00 | 4 | 1 |
| 326 | 109 | 1 | 109 | 2 | 6.53 | 7.00 | 6.00 | 5 | 1 |
| 327 | 109 | 1 | 109 | 3 | 4.30 | 5.50 | 4.50 | 5 | 1 |
| 328 | 110 | 1 | 110 | 1 | 5.41 | 6.00 | 4.00 | 4 | 1 |
| 329 | 110 | 1 | 110 | 2 | 8.28 | 8.00 | 6.00 | 4 | 1 |
| 330 | 110 | 1 | 110 | 3 | 9.55 | 8.20 | 6.00 | 4 | 1 |
| 331 | 111 | 1 | 111 | 1 | 9.36 | 10.00 | 8.50 | 4 | 1 |
| 332 | 111 | 1 | 111 | 2 | 8.40 | 9.50 | 7.50 | 5 | 1 |
| 333 | 111 | 1 | 111 | 3 | 11.27 | 9.80 | 8.50 | 5 | 1 |
| 334 | 112 | 1 | 112 | 1 | 6.43 | 6.20 | 4.80 | 5 | 1 |
| 335 | 112 | 1 | 112 | 2 | 3.18 | 4.00 | 3.00 | 5 | 1 |
| 336 | 112 | 1 | 112 | 3 | 4.42 | 4.00 | 3.00 | 5 | 1 |
| 337 | 113 | 1 | 113 | 1 | 2.55 | 3.50 | 3.00 | 5 | 1 |
| 338 | 113 | 1 | 113 | 2 | 0.00 | 0.00 | 0.00 | 0 | 0 |
| 339 | 113 | 1 | 113 | 3 | 7.64 | 7.00 | 5.00 | 4 | 1 |
| 340 | 114 | 1 | 114 | 1 | 6.68 | 6.50 | 6.00 | 5 | 1 |
| 341 | 114 | 1 | 114 | 2 | 3.98 | 5.00 | 4.50 | 4 | 1 |
| 342 | 114 | 1 | 114 | 3 | 11.87 | 10.50 | 7.20 | 5 | 1 |
| 343 | 115 | 1 | 115 | 1 | 7.32 | 7.00 | 6.00 | 4 | 1 |
| 344 | 115 | 1 | 115 | 2 | 2.23 | 3.00 | 1.80 | 4 | 1 |
| 345 | 115 | 1 | 115 | 3 | 5.57 | 6.00 | 5.50 | 4 | 1 |
| 346 | 116 | 1 | 116 | 1 | 4.81 | 4.00 | 2.80 | 4 | 1 |
| 347 | 116 | 1 | 116 | 2 | 9.23 | 7.50 | 6.50 | 5 | 1 |
| 348 | 116 | 1 | 116 | 3 | 4.97 | 5.00 | 4.00 | 5 | 1 |
| 349 | 117 | 1 | 117 | 1 | 5.09 | 5.40 | 3.80 | 4 | 1 |
| 350 | 117 | 1 | 117 | 2 | 7.64 | 7.00 | 5.80 | 5 | 1 |
| 351 | 117 | 1 | 117 | 3 | 9.52 | 7.20 | 5.20 | 5 | 1 |
| 352 | 118 | 1 | 118 | 1 | 2.42 | 4.00 | 3.00 | 4 | 1 |
| 353 | 118 | 1 | 118 | 2 | 1.72 | 3.50 | 1.50 | 5 | 1 |
| 354 | 118 | 1 | 118 | 3 | 2.86 | 3.00 | 1.60 | 4 | 1 |
| 355 | 119 | 1 | 119 | 1 | 3.34 | 6.50 | 6.00 | 4 | 1 |
| 356 | 119 | 1 | 119 | 2 | 4.93 | 8.00 | 7.00 | 4 | 1 |
| 357 | 119 | 1 | 119 | 3 | 12.83 | 12.00 | 11.00 | 5 | 1 |
| 358 | 120 | 1 | 120 | 1 | 7.96 | 10.00 | 8.00 | 5 | 1 |
| 359 | 120 | 1 | 120 | 2 | 11.46 | 11.00 | 9.00 | 4 | 1 |
| 360 | 120 | 1 | 120 | 3 | 8.88 | 9.50 | 8.00 | 5 | 1 |
| 361 | 121 | 1 | 121 | 1 | 5.19 | 6.50 | 4.50 | 4 | 1 |
| 362 | 121 | 1 | 121 | 2 | 3.92 | 3.50 | 1.80 | 4 | 1 |
| 363 | 121 | 1 | 121 | 3 | 3.18 | 3.10 | 2.70 | 4 | 1 |
| 364 | 122 | 1 | 122 | 1 | 4.14 | 4.80 | 4.00 | 4 | 1 |
| 365 | 122 | 1 | 122 | 2 | 1.59 | 2.60 | 1.50 | 4 | 1 |
| 366 | 122 | 1 | 122 | 3 | 2.07 | 4.20 | 3.80 | 5 | 1 |
| 367 | 123 | 1 | 123 | 1 | 3.66 | 7.00 | 6.00 | 5 | 1 |
| 368 | 123 | 1 | 123 | 2 | 0.00 | 0.00 | 0.00 | 0 | 0 |
| 369 | 123 | 1 | 123 | 3 | 3.18 | 6.50 | 4.50 | 4 | 1 |
| 370 | 124 | 1 | 124 | 1 | 4.46 | 6.00 | 5.00 | 5 | 1 |
| 371 | 124 | 1 | 124 | 2 | 4.62 | 6.50 | 5.50 | 5 | 1 |
| 372 | 124 | 1 | 124 | 3 | 3.50 | 5.80 | 4.80 | 5 | 1 |
| 373 | 125 | 1 | 125 | 1 | 5.22 | 6.10 | 4.80 | 4 | 1 |
| 374 | 125 | 1 | 125 | 2 | 6.84 | 6.50 | 5.50 | 4 | 1 |
| 375 | 125 | 1 | 125 | 3 | 4.77 | 5.00 | 4.00 | 4 | 1 |
| 376 | 126 | 1 | 126 | 1 | 12.70 | 11.00 | 9.00 | 4 | 1 |
| 377 | 126 | 1 | 126 | 2 | 8.79 | 9.00 | 8.00 | 4 | 1 |
| 378 | 126 | 1 | 126 | 3 | 11.71 | 8.00 | 6.00 | 4 | 1 |
| 379 | 127 | 1 | 127 | 1 | 3.18 | 6.00 | 5.00 | 5 | 1 |
| 380 | 127 | 1 | 127 | 2 | 2.16 | 5.00 | 4.00 | 4 | 1 |
| 381 | 127 | 1 | 127 | 3 | 7.13 | 8.50 | 7.50 | 5 | 1 |
| 382 | 128 | 1 | 128 | 1 | 3.98 | 3.80 | 3.00 | 4 | 1 |
| 383 | 128 | 1 | 128 | 2 | 6.33 | 5.00 | 4.00 | 4 | 1 |
| 384 | 128 | 1 | 128 | 3 | 2.58 | 3.10 | 2.50 | 4 | 1 |
| 385 | 129 | 1 | 129 | 1 | 3.87 | 9.00 | 6.00 | 5 | 1 |
| 386 | 129 | 1 | 129 | 2 | 10.92 | 10.50 | 8.00 | 5 | 1 |
| 387 | 129 | 1 | 129 | 3 | 12.61 | 13.00 | 8.00 | 5 | 1 |
| 388 | 130 | 1 | 130 | 1 | 8.72 | 9.30 | 8.00 | 4 | 1 |
| 389 | 130 | 1 | 130 | 2 | 5.54 | 6.50 | 5.50 | 5 | 1 |
| 390 | 130 | 1 | 130 | 3 | 12.73 | 11.00 | 9.00 | 5 | 1 |
| 391 | 131 | 1 | 131 | 1 | 5.09 | 6.00 | 5.00 | 4 | 1 |
| 392 | 131 | 1 | 131 | 2 | 0.00 | 0.00 | 0.00 | 0 | 0 |
| 393 | 131 | 1 | 131 | 3 | 8.75 | 7.00 | 5.00 | 4 | 1 |
| 394 | 132 | 1 | 132 | 1 | 0.00 | 0.00 | 0.00 | 0 | 0 |
| 395 | 132 | 1 | 132 | 2 | 2.64 | 4.00 | 3.50 | 4 | 1 |
| 396 | 132 | 1 | 132 | 3 | 5.22 | 6.00 | 5.00 | 4 | 1 |
| 397 | 1 | 2 | 133 | 1 | 9.61 | 11.50 | 8.00 | 4 | 1 |
| 398 | 1 | 2 | 133 | 2 | 5.51 | 9.50 | 8.00 | 4 | 1 |
| 399 | 1 | 2 | 133 | 3 | 10.19 | 11.50 | 9.00 | 5 | 1 |
| 400 | 2 | 2 | 134 | 1 | 3.66 | 4.50 | 3.00 | 5 | 1 |
| 401 | 2 | 2 | 134 | 2 | 6.43 | 8.50 | 5.50 | 5 | 1 |
| 402 | 2 | 2 | 134 | 3 | 6.75 | 8.50 | 6.00 | 5 | 1 |
| 403 | 3 | 2 | 135 | 1 | 13.69 | 14.00 | 6.50 | 5 | 1 |
| 404 | 3 | 2 | 135 | 2 | 7.99 | 9.00 | 6.50 | 5 | 1 |
| 405 | 3 | 2 | 135 | 3 | 11.87 | 13.00 | 6.00 | 5 | 1 |
| 406 | 4 | 2 | 136 | 1 | 5.60 | 7.50 | 5.50 | 5 | 1 |
| 407 | 4 | 2 | 136 | 2 | 10.54 | 9.50 | 6.00 | 5 | 1 |
| 408 | 4 | 2 | 136 | 3 | 7.89 | 8.50 | 6.50 | 5 | 1 |
| 409 | 5 | 2 | 137 | 1 | 7.83 | 8.20 | 2.80 | 4 | 1 |
| 410 | 5 | 2 | 137 | 2 | 2.77 | 3.30 | 2.80 | 4 | 1 |
| 411 | 5 | 2 | 137 | 3 | 8.98 | 10.70 | 7.50 | 5 | 1 |
| 412 | 6 | 2 | 138 | 1 | 9.49 | 9.50 | 5.50 | 5 | 1 |
| 413 | 6 | 2 | 138 | 2 | 5.73 | 9.00 | 5.00 | 4 | 1 |
| 414 | 6 | 2 | 138 | 3 | 11.24 | 12.00 | 8.50 | 5 | 1 |
| 415 | 7 | 2 | 139 | 1 | 14.23 | 15.00 | 8.00 | 5 | 1 |
| 416 | 7 | 2 | 139 | 2 | 11.33 | 14.50 | 8.50 | 5 | 1 |
| 417 | 7 | 2 | 139 | 3 | 11.71 | 14.00 | 7.50 | 5 | 1 |
| 418 | 8 | 2 | 140 | 1 | 7.73 | 8.30 | 5.50 | 4 | 1 |
| 419 | 8 | 2 | 140 | 2 | 2.93 | 6.00 | 4.00 | 3 | 1 |
| 420 | 8 | 2 | 140 | 3 | 0.00 | 0.00 | 0.00 | 0 | 0 |
| 421 | 9 | 2 | 141 | 1 | 8.82 | 8.70 | 5.00 | 5 | 1 |
| 422 | 9 | 2 | 141 | 2 | 8.47 | 9.00 | 5.50 | 5 | 1 |
| 423 | 9 | 2 | 141 | 3 | 5.32 | 9.30 | 3.80 | 5 | 1 |
| 424 | 10 | 2 | 142 | 1 | 0.00 | 0.00 | 0.00 | 0 | 0 |
| 425 | 10 | 2 | 142 | 2 | 0.00 | 0.00 | 0.00 | 0 | 0 |
| 426 | 10 | 2 | 142 | 3 | 5.60 | 8.00 | 6.50 | 5 | 1 |
| 427 | 11 | 2 | 143 | 1 | 10.44 | 10.50 | 7.50 | 4 | 1 |
| 428 | 11 | 2 | 143 | 2 | 12.61 | 11.50 | 7.00 | 4 | 1 |
| 429 | 11 | 2 | 143 | 3 | 0.00 | 0.00 | 0.00 | 0 | 0 |
| 430 | 12 | 2 | 144 | 1 | 12.57 | 14.30 | 8.50 | 5 | 1 |
| 431 | 12 | 2 | 144 | 2 | 19.19 | 15.30 | 10.50 | 5 | 1 |
| 432 | 12 | 2 | 144 | 3 | 12.13 | 12.50 | 8.50 | 5 | 1 |
| 433 | 13 | 2 | 145 | 1 | 9.39 | 10.00 | 7.50 | 5 | 1 |
| 434 | 13 | 2 | 145 | 2 | 2.83 | 4.60 | 3.60 | 5 | 1 |
| 435 | 13 | 2 | 145 | 3 | 9.84 | 9.70 | 5.00 | 5 | 1 |
| 436 | 14 | 2 | 146 | 1 | 9.04 | 12.00 | 6.50 | 5 | 1 |
| 437 | 14 | 2 | 146 | 2 | 6.75 | 11.00 | 7.00 | 3 | 1 |
| 438 | 14 | 2 | 146 | 3 | 5.89 | 9.50 | 7.50 | 4 | 1 |
| 439 | 15 | 2 | 147 | 1 | 9.61 | 10.70 | 8.50 | 4 | 1 |
| 440 | 15 | 2 | 147 | 2 | 10.63 | 11.00 | 8.00 | 5 | 1 |
| 441 | 15 | 2 | 147 | 3 | 15.18 | 12.50 | 8.50 | 5 | 1 |
| 442 | 16 | 2 | 148 | 1 | 15.15 | 12.50 | 8.00 | 5 | 1 |
| 443 | 16 | 2 | 148 | 2 | 2.31 | 6.00 | 2.50 | 5 | 1 |
| 444 | 16 | 2 | 148 | 3 | 9.49 | 9.00 | 6.00 | 4 | 1 |
| 445 | 17 | 2 | 149 | 1 | 5.98 | 8.20 | 5.00 | 5 | 1 |
| 446 | 17 | 2 | 149 | 2 | 7.73 | 8.50 | 5.00 | 5 | 1 |
| 447 | 17 | 2 | 149 | 3 | 7.58 | 8.70 | 5.00 | 5 | 1 |
| 448 | 18 | 2 | 150 | 1 | 12.73 | 15.50 | 10.00 | 5 | 1 |
| 449 | 18 | 2 | 150 | 2 | 11.52 | 15.50 | 9.50 | 5 | 1 |
| 450 | 18 | 2 | 150 | 3 | 11.17 | 14.50 | 5.50 | 5 | 1 |
| 451 | 19 | 2 | 151 | 1 | 12.57 | 14.50 | 9.00 | 5 | 1 |
| 452 | 19 | 2 | 151 | 2 | 11.01 | 14.50 | 9.50 | 5 | 1 |
| 453 | 19 | 2 | 151 | 3 | 13.11 | 14.00 | 9.50 | 5 | 1 |
| 454 | 20 | 2 | 152 | 1 | 4.27 | 4.40 | 3.80 | 5 | 1 |
| 455 | 20 | 2 | 152 | 2 | 4.42 | 4.20 | 3.00 | 5 | 1 |
| 456 | 20 | 2 | 152 | 3 | 3.69 | 4.00 | 3.10 | 5 | 1 |
| 457 | 21 | 2 | 153 | 1 | 10.50 | 11.00 | 7.00 | 5 | 1 |
| 458 | 21 | 2 | 153 | 2 | 0.00 | 0.00 | 0.00 | 0 | 0 |
| 459 | 21 | 2 | 153 | 3 | 8.34 | 10.10 | 7.00 | 5 | 1 |
| 460 | 22 | 2 | 154 | 1 | 14.96 | 12.00 | 7.00 | 5 | 1 |
| 461 | 22 | 2 | 154 | 2 | 17.00 | 12.50 | 8.00 | 5 | 1 |
| 462 | 22 | 2 | 154 | 3 | 6.75 | 8.50 | 6.00 | 5 | 1 |
| 463 | 23 | 2 | 155 | 1 | 0.00 | 0.00 | 0.00 | 0 | 0 |
| 464 | 23 | 2 | 155 | 2 | 8.53 | 10.00 | 6.50 | 4 | 1 |
| 465 | 23 | 2 | 155 | 3 | 4.74 | 6.50 | 4.50 | 4 | 1 |
| 466 | 24 | 2 | 156 | 1 | 0.00 | 0.00 | 0.00 | 0 | 0 |
| 467 | 24 | 2 | 156 | 2 | 12.16 | 10.00 | 5.00 | 5 | 1 |
| 468 | 24 | 2 | 156 | 3 | 13.18 | 10.20 | 4.50 | 5.00 | 1 |
| 469 | 25 | 2 | 157 | 1 | 4.39 | 5.00 | 4.00 | 5 | 1 |
| 470 | 25 | 2 | 157 | 2 | 3.88 | 4.50 | 3.50 | 4 | 1 |
| 471 | 25 | 2 | 157 | 3 | 6.43 | 5.50 | 4.50 | 3 | 1 |
| 472 | 26 | 2 | 158 | 1 | 9.36 | 6.50 | 4.50 | 5 | 1 |
| 473 | 26 | 2 | 158 | 2 | 5.19 | 4.30 | 3.30 | 4 | 1 |
| 474 | 26 | 2 | 158 | 3 | 9.04 | 5.50 | 4.00 | 5 | 1 |
| 475 | 27 | 2 | 159 | 1 | 13.18 | 12.20 | 8.50 | 5 | 1 |
| 476 | 27 | 2 | 159 | 2 | 14.96 | 13.30 | 8.50 | 5 | 1 |
| 477 | 27 | 2 | 159 | 3 | 15.76 | 12.50 | 8.50 | 5 | 1 |
| 478 | 28 | 2 | 160 | 1 | 3.88 | 5.00 | 3.00 | 3 | 1 |
| 479 | 28 | 2 | 160 | 2 | 0.00 | 0.00 | 0.00 | 0 | 0 |
| 480 | 28 | 2 | 160 | 3 | 4.93 | 6.00 | 4.00 | 3 | 1 |
| 481 | 29 | 2 | 161 | 1 | 5.35 | 6.00 | 3.80 | 5 | 1 |
| 482 | 29 | 2 | 161 | 2 | 9.14 | 6.80 | 3.60 | 2 | 1 |
| 483 | 29 | 2 | 161 | 3 | 5.57 | 7.50 | 5.00 | 5 | 1 |
| 484 | 30 | 2 | 162 | 1 | 8.12 | 11.00 | 6.50 | 5 | 1 |
| 485 | 30 | 2 | 162 | 2 | 0.00 | 0.00 | 0.00 | 0 | 0 |
| 486 | 30 | 2 | 162 | 3 | 13.50 | 12.00 | 9.50 | 5 | 1 |
| 487 | 31 | 2 | 163 | 1 | 4.71 | 4.00 | 3.30 | 5 | 1 |
| 488 | 31 | 2 | 163 | 2 | 0.00 | 0.00 | 0.00 | 0 | 0 |
| 489 | 31 | 2 | 163 | 3 | 7.73 | 6.50 | 4.30 | 4 | 1 |
| 490 | 32 | 2 | 164 | 1 | 7.96 | 11.00 | 8.00 | 5 | 1 |
| 491 | 32 | 2 | 164 | 2 | 17.60 | 12.00 | 6.50 | 5 | 1 |
| 492 | 32 | 2 | 164 | 3 | 9.20 | 8.00 | 5.00 | 4 | 1 |
| 493 | 33 | 2 | 165 | 1 | 8.26 | 12.00 | 7.50 | 4 | 1 |
| 494 | 33 | 2 | 165 | 2 | 5.83 | 8.50 | 6.00 | 4 | 1 |
| 495 | 33 | 2 | 165 | 3 | 9.90 | 10.00 | 3.50 | 4 | 1 |
| 496 | 34 | 2 | 166 | 1 | 6.11 | 7.30 | 5.40 | 5 | 1 |
| 497 | 34 | 2 | 166 | 2 | 3.44 | 4.60 | 2.80 | 5 | 1 |
| 498 | 34 | 2 | 166 | 3 | 6.30 | 7.20 | 4.20 | 5 | 1 |
| 499 | 35 | 2 | 167 | 1 | 10.82 | 14.50 | 9.50 | 4 | 1 |
| 500 | 35 | 2 | 167 | 2 | 13.11 | 15.00 | 9.00 | 5 | 1 |
| 501 | 35 | 2 | 167 | 3 | 10.09 | 14.00 | 8.00 | 5 | 1 |
| 502 | 36 | 2 | 168 | 1 | 2.64 | 1.50 | 1.00 | 3 | 1 |
| 503 | 36 | 2 | 168 | 2 | 4.04 | 4.00 | 2.00 | 5 | 1 |
| 504 | 36 | 2 | 168 | 3 | 6.08 | 5.00 | 4.00 | 5 | 1 |
| 505 | 37 | 2 | 169 | 1 | 8.56 | 9.10 | 5.00 | 5 | 1 |
| 506 | 37 | 2 | 169 | 2 | 9.55 | 9.30 | 5.00 | 5 | 1 |
| 507 | 37 | 2 | 169 | 3 | 10.66 | 9.50 | 6.00 | 5 | 1 |
| 508 | 38 | 2 | 170 | 1 | 1.43 | 2.50 | 2.00 | 5 | 1 |
| 509 | 38 | 2 | 170 | 2 | 6.05 | 7.50 | 6.00 | 4 | 1 |
| 510 | 38 | 2 | 170 | 3 | 1.43 | 3.00 | 2.00 | 4 | 1 |
| 511 | 39 | 2 | 171 | 1 | 11.68 | 12.50 | 7.50 | 5 | 1 |
| 512 | 39 | 2 | 171 | 2 | 13.94 | 12.80 | 8.00 | 5 | 1 |
| 513 | 39 | 2 | 171 | 3 | 10.57 | 12.50 | 8.00 | 5 | 1 |
| 514 | 40 | 2 | 172 | 1 | 16.74 | 12.50 | 9.00 | 3 | 1 |
| 515 | 40 | 2 | 172 | 2 | 13.08 | 12.00 | 8.50 | 5 | 1 |
| 516 | 40 | 2 | 172 | 3 | 8.34 | 9.00 | 6.50 | 5 | 1 |
| 517 | 41 | 2 | 173 | 1 | 3.44 | 7.50 | 6.00 | 5 | 1 |
| 518 | 41 | 2 | 173 | 2 | 7.80 | 10.50 | 7.50 | 5 | 1 |
| 519 | 41 | 2 | 173 | 3 | 13.50 | 11.00 | 8.00 | 5 | 1 |
| 520 | 42 | 2 | 174 | 1 | 10.22 | 9.20 | 5.70 | 5 | 1 |
| 521 | 42 | 2 | 174 | 2 | 6.53 | 7.50 | 5.00 | 5 | 1 |
| 522 | 42 | 2 | 174 | 3 | 11.17 | 9.00 | 6.00 | 5 | 1 |
| 523 | 43 | 2 | 175 | 1 | 2.48 | 3.60 | 3.10 | 5 | 1 |
| 524 | 43 | 2 | 175 | 2 | 3.34 | 3.70 | 3.10 | 5 | 1 |
| 525 | 43 | 2 | 175 | 3 | 7.42 | 6.50 | 5.00 | 5 | 1 |
| 526 | 44 | 2 | 176 | 1 | 0.00 | 0.00 | 0.00 | 0 | 0 |
| 527 | 44 | 2 | 176 | 2 | 5.67 | 9.50 | 6.50 | 3 | 1 |
| 528 | 44 | 2 | 176 | 3 | 8.50 | 11.50 | 7.50 | 5 | 1 |
| 529 | 45 | 2 | 177 | 1 | 6.84 | 7.00 | 3.10 | 3 | 1 |
| 530 | 45 | 2 | 177 | 2 | 12.86 | 11.00 | 6.00 | 3 | 1 |
| 531 | 45 | 2 | 177 | 3 | 10.66 | 11.30 | 7.50 | 5 | 1 |
| 532 | 46 | 2 | 178 | 1 | 8.56 | 7.30 | 5.70 | 5 | 1 |
| 533 | 46 | 2 | 178 | 2 | 9.55 | 9.50 | 6.50 | 5 | 1 |
| 534 | 46 | 2 | 178 | 3 | 9.64 | 9.00 | 6.00 | 5 | 1 |
| 535 | 47 | 2 | 179 | 1 | 4.39 | 4.00 | 3.20 | 5 | 1 |
| 536 | 47 | 2 | 179 | 2 | 4.55 | 4.00 | 3.50 | 5 | 1 |
| 537 | 47 | 2 | 179 | 3 | 3.88 | 4.00 | 3.60 | 5 | 1 |
| 538 | 48 | 2 | 180 | 1 | 6.88 | 11.50 | 6.50 | 5 | 1 |
| 539 | 48 | 2 | 180 | 2 | 10.12 | 10.80 | 6.00 | 5 | 1 |
| 540 | 48 | 2 | 180 | 3 | 12.67 | 10.50 | 6.50 | 5 | 1 |
| 541 | 49 | 2 | 181 | 1 | 11.94 | 11.20 | 6.00 | 5 | 1 |
| 542 | 49 | 2 | 181 | 2 | 5.16 | 6.50 | 4.50 | 4 | 1 |
| 543 | 49 | 2 | 181 | 3 | 5.54 | 7.80 | 5.00 | 5 | 1 |
| 544 | 50 | 2 | 182 | 1 | 5.92 | 6.00 | 4.50 | 4 | 1 |
| 545 | 50 | 2 | 182 | 2 | 7.83 | 6.50 | 4.50 | 5 | 1 |
| 546 | 50 | 2 | 182 | 3 | 7.58 | 6.50 | 4.50 | 5 | 1 |
| 547 | 51 | 2 | 183 | 1 | 11.05 | 11.50 | 8.50 | 5 | 1 |
| 548 | 51 | 2 | 183 | 2 | 12.54 | 11.50 | 8.00 | 4 | 1 |
| 549 | 51 | 2 | 183 | 3 | 12.35 | 11.50 | 7.00 | 3 | 1 |
| 550 | 52 | 2 | 184 | 1 | 7.51 | 9.00 | 7.00 | 5 | 1 |
| 551 | 52 | 2 | 184 | 2 | 3.79 | 5.20 | 3.70 | 5 | 1 |
| 552 | 52 | 2 | 184 | 3 | 4.36 | 5.50 | 3.80 | 5 | 1 |
| 553 | 53 | 2 | 185 | 1 | 12.73 | 10.00 | 7.30 | 5 | 1 |
| 554 | 53 | 2 | 185 | 2 | 10.57 | 9.80 | 7.00 | 5 | 1 |
| 555 | 53 | 2 | 185 | 3 | 8.47 | 9.00 | 6.80 | 5 | 1 |
| 556 | 54 | 2 | 186 | 1 | 9.07 | 9.00 | 5.00 | 5 | 1 |
| 557 | 54 | 2 | 186 | 2 | 3.57 | 5.50 | 4.00 | 5 | 1 |
| 558 | 54 | 2 | 186 | 3 | 9.39 | 8.50 | 5.00 | 5 | 1 |
| 559 | 55 | 2 | 187 | 1 | 5.06 | 5.00 | 3.50 | 5 | 1 |
| 560 | 55 | 2 | 187 | 2 | 5.73 | 5.50 | 3.50 | 5 | 1 |
| 561 | 55 | 2 | 187 | 3 | 5.47 | 5.00 | 4.00 | 5 | 1 |
| 562 | 56 | 2 | 188 | 1 | 8.72 | 9.50 | 6.50 | 5 | 1 |
| 563 | 56 | 2 | 188 | 2 | 5.68 | 11.00 | 4.50 | 4 | 1 |
| 564 | 56 | 2 | 188 | 3 | 9.52 | 12.00 | 7.50 | 4 | 1 |
| 565 | 57 | 2 | 189 | 1 | 14.58 | 12.50 | 9.50 | 5 | 1 |
| 566 | 57 | 2 | 189 | 2 | 15.76 | 12.50 | 8.50 | 5 | 1 |
| 567 | 57 | 2 | 189 | 3 | 8.37 | 9.50 | 7.00 | 4 | 1 |
| 568 | 58 | 2 | 190 | 1 | 7.58 | 10.80 | 7.80 | 5 | 1 |
| 569 | 58 | 2 | 190 | 2 | 5.73 | 8.50 | 6.00 | 4 | 1 |
| 570 | 58 | 2 | 190 | 3 | 8.72 | 11.00 | 7.50 | 5 | 1 |
| 571 | 59 | 2 | 191 | 1 | 10.47 | 11.80 | 8.00 | 5 | 1 |
| 572 | 59 | 2 | 191 | 2 | 7.93 | 10.00 | 6.50 | 5 | 1 |
| 573 | 59 | 2 | 191 | 3 | 10.25 | 10.70 | 8.50 | 5 | 1 |
| 574 | 60 | 2 | 192 | 1 | 0.00 | 0.00 | 0.00 | 0 | 0 |
| 575 | 60 | 2 | 192 | 2 | 5.95 | 5.00 | 4.00 | 5 | 1 |
| 576 | 60 | 2 | 192 | 3 | 2.29 | 4.00 | 3.50 | 5 | 1 |
| 577 | 61 | 2 | 193 | 1 | 9.87 | 10.50 | 7.50 | 5 | 1 |
| 578 | 61 | 2 | 193 | 2 | 9.61 | 10.00 | 8.00 | 5 | 1 |
| 579 | 61 | 2 | 193 | 3 | 11.78 | 10.50 | 7.00 | 4 | 1 |
| 580 | 62 | 2 | 194 | 1 | 6.24 | 10.50 | 5.50 | 5 | 1 |
| 581 | 62 | 2 | 194 | 2 | 5.38 | 10.00 | 4.50 | 5 | 1 |
| 582 | 62 | 2 | 194 | 3 | 6.68 | 10.50 | 6.00 | 5 | 1 |
| 583 | 63 | 2 | 195 | 1 | 8.98 | 9.00 | 6.00 | 5 | 1 |
| 584 | 63 | 2 | 195 | 2 | 8.15 | 9.30 | 6.50 | 4 | 1 |
| 585 | 63 | 2 | 195 | 3 | 9.49 | 9.20 | 6.50 | 4 | 1 |
| 586 | 64 | 2 | 196 | 1 | 8.05 | 9.30 | 6.00 | 4 | 1 |
| 587 | 64 | 2 | 196 | 2 | 15.22 | 10.90 | 7.50 | 4 | 1 |
| 588 | 64 | 2 | 196 | 3 | 10.54 | 10.30 | 6.30 | 4 | 1 |
| 589 | 65 | 2 | 197 | 1 | 7.03 | 9.50 | 7.00 | 5 | 1 |
| 590 | 65 | 2 | 197 | 2 | 0.00 | 0.00 | 0.00 | 0 | 0 |
| 591 | 65 | 2 | 197 | 3 | 0.00 | 0.00 | 0.00 | 0 | 0 |
| 592 | 66 | 2 | 198 | 1 | 4.81 | 7.20 | 5.40 | 5 | 1 |
| 593 | 66 | 2 | 198 | 2 | 5.60 | 7.80 | 6.00 | 4 | 1 |
| 594 | 66 | 2 | 198 | 3 | 4.27 | 7.00 | 5.00 | 4 | 1 |
| 595 | 67 | 2 | 199 | 1 | 0.00 | 0.00 | 0.00 | 0 | 0 |
| 596 | 67 | 2 | 199 | 2 | 2.96 | 4.50 | 3.00 | 3 | 1 |
| 597 | 67 | 2 | 199 | 3 | 9.39 | 9.50 | 5.00 | 5 | 1 |
| 598 | 68 | 2 | 200 | 1 | 4.39 | 6.00 | 3.30 | 5 | 1 |
| 599 | 68 | 2 | 200 | 2 | 6.05 | 7.70 | 5.00 | 5 | 1 |
| 600 | 68 | 2 | 200 | 3 | 6.81 | 8.00 | 6.00 | 5 | 1 |
| 601 | 69 | 2 | 201 | 1 | 5.60 | 5.50 | 4.00 | 5 | 1 |
| 602 | 69 | 2 | 201 | 2 | 6.27 | 5.50 | 4.00 | 5 | 1 |
| 603 | 69 | 2 | 201 | 3 | 6.81 | 5.50 | 4.00 | 4 | 1 |
| 604 | 70 | 2 | 202 | 1 | 9.42 | 12.80 | 8.50 | 5 | 1 |
| 605 | 70 | 2 | 202 | 2 | 9.29 | 12.50 | 9.00 | 5 | 1 |
| 606 | 70 | 2 | 202 | 3 | 8.37 | 8.50 | 6.00 | 5 | 1 |
| 607 | 71 | 2 | 203 | 1 | 6.40 | 9.00 | 7.00 | 5 | 1 |
| 608 | 71 | 2 | 203 | 2 | 7.96 | 9.50 | 7.50 | 5 | 1 |
| 609 | 71 | 2 | 203 | 3 | 10.98 | 10.00 | 7.50 | 5 | 1 |
| 610 | 72 | 2 | 204 | 1 | 8.72 | 11.50 | 5.50 | 5 | 1 |
| 611 | 72 | 2 | 204 | 2 | 8.12 | 12.00 | 6.50 | 5 | 1 |
| 612 | 72 | 2 | 204 | 3 | 6.62 | 11.00 | 6.00 | 5 | 1 |
| 613 | 73 | 2 | 205 | 1 | 3.53 | 3.80 | 2.70 | 3 | 1 |
| 614 | 73 | 2 | 205 | 2 | 4.77 | 4.30 | 3.60 | 4 | 1 |
| 615 | 73 | 2 | 205 | 3 | 7.10 | 5.80 | 3.20 | 5 | 1 |
| 616 | 74 | 2 | 206 | 1 | 14.51 | 9.70 | 5.50 | 5 | 1 |
| 617 | 74 | 2 | 206 | 2 | 9.58 | 9.20 | 6.20 | 5 | 1 |
| 618 | 74 | 2 | 206 | 3 | 12.54 | 10.50 | 7.00 | 4 | 1 |
| 619 | 75 | 2 | 207 | 1 | 6.30 | 9.50 | 4.00 | 4 | 1 |
| 620 | 75 | 2 | 207 | 2 | 10.47 | 11.00 | 4.50 | 5 | 1 |
| 621 | 75 | 2 | 207 | 3 | 13.62 | 11.00 | 6.00 | 5 | 1 |
| 622 | 76 | 2 | 208 | 1 | 3.31 | 4.00 | 3.00 | 3 | 1 |
| 623 | 76 | 2 | 208 | 2 | 3.25 | 3.50 | 3.00 | 3 | 1 |
| 624 | 76 | 2 | 208 | 3 | 3.44 | 3.30 | 2.80 | 3 | 1 |
| 625 | 77 | 2 | 209 | 1 | 5.16 | 5.00 | 3.50 | 4 | 1 |
| 626 | 77 | 2 | 209 | 2 | 4.81 | 5.20 | 4.40 | 4 | 1 |
| 627 | 77 | 2 | 209 | 3 | 10.54 | 7.50 | 5.00 | 5 | 1 |
| 628 | 78 | 2 | 210 | 1 | 14.23 | 11.00 | 8.00 | 5 | 1 |
| 629 | 78 | 2 | 210 | 2 | 8.15 | 9.00 | 6.00 | 4 | 1 |
| 630 | 78 | 2 | 210 | 3 | 11.01 | 10.80 | 8.00 | 5 | 1 |
| 631 | 79 | 2 | 211 | 1 | 4.77 | 6.50 | 3.50 | 5 | 1 |
| 632 | 79 | 2 | 211 | 2 | 8.79 | 9.50 | 6.50 | 5 | 1 |
| 633 | 79 | 2 | 211 | 3 | 9.55 | 9.50 | 5.00 | 5 | 1 |
| 634 | 80 | 2 | 212 | 1 | 14.01 | 11.50 | 8.00 | 5 | 1 |
| 635 | 80 | 2 | 212 | 2 | 14.01 | 11.50 | 8.50 | 5 | 1 |
| 636 | 80 | 2 | 212 | 3 | 13.11 | 11.80 | 8.70 | 5 | 1 |
| 637 | 81 | 2 | 213 | 1 | 14.10 | 12.00 | 9.00 | 4 | 1 |
| 638 | 81 | 2 | 213 | 2 | 15.98 | 11.50 | 7.50 | 4 | 1 |
| 639 | 81 | 2 | 213 | 3 | 11.43 | 11.00 | 8.50 | 4 | 1 |
| 640 | 82 | 2 | 214 | 1 | 8.40 | 9.50 | 7.50 | 5 | 1 |
| 641 | 82 | 2 | 214 | 2 | 15.12 | 12.00 | 9.00 | 5 | 1 |
| 642 | 82 | 2 | 214 | 3 | 12.92 | 11.20 | 7.50 | 4 | 1 |
| 643 | 83 | 2 | 215 | 1 | 13.66 | 10.00 | 7.00 | 5 | 1 |
| 644 | 83 | 2 | 215 | 2 | 10.85 | 10.50 | 7.50 | 4 | 1 |
| 645 | 83 | 2 | 215 | 3 | 14.96 | 11.00 | 7.50 | 5 | 1 |
| 646 | 84 | 2 | 216 | 1 | 2.90 | 5.20 | 3.60 | 5 | 1 |
| 647 | 84 | 2 | 216 | 2 | 6.11 | 7.50 | 5.50 | 4 | 1 |
| 648 | 84 | 2 | 216 | 3 | 8.75 | 8.50 | 5.00 | 5 | 1 |
| 649 | 85 | 2 | 217 | 1 | 10.12 | 10.90 | 5.50 | 5 | 1 |
| 650 | 85 | 2 | 217 | 2 | 10.03 | 10.30 | 5.00 | 5 | 1 |
| 651 | 85 | 2 | 217 | 3 | 6.49 | 9.00 | 4.80 | 5 | 1 |
| 652 | 86 | 2 | 218 | 1 | 9.52 | 11.00 | 7.50 | 5 | 1 |
| 653 | 86 | 2 | 218 | 2 | 3.34 | 7.20 | 6.00 | 5 | 1 |
| 654 | 86 | 2 | 218 | 3 | 11.94 | 11.50 | 7.80 | 5 | 1 |
| 655 | 87 | 2 | 219 | 1 | 6.49 | 7.70 | 5.00 | 5 | 1 |
| 656 | 87 | 2 | 219 | 2 | 8.94 | 8.50 | 4.50 | 5 | 1 |
| 657 | 87 | 2 | 219 | 3 | 5.25 | 8.00 | 5.00 | 3 | 1 |
| 658 | 88 | 2 | 220 | 1 | 5.47 | 6.00 | 5.00 | 5 | 1 |
| 659 | 88 | 2 | 220 | 2 | 5.70 | 6.00 | 4.50 | 5 | 1 |
| 660 | 88 | 2 | 220 | 3 | 6.14 | 6.00 | 5.00 | 5 | 1 |
| 661 | 89 | 2 | 221 | 1 | 6.94 | 11.50 | 6.00 | 5 | 1 |
| 662 | 89 | 2 | 221 | 2 | 8.12 | 11.50 | 7.50 | 5 | 1 |
| 663 | 89 | 2 | 221 | 3 | 8.34 | 11.50 | 6.50 | 5 | 1 |
| 664 | 90 | 2 | 222 | 1 | 15.85 | 12.50 | 9.00 | 4 | 1 |
| 665 | 90 | 2 | 222 | 2 | 14.45 | 12.00 | 9.00 | 5 | 1 |
| 666 | 90 | 2 | 222 | 3 | 14.20 | 11.50 | 9.00 | 5 | 1 |
| 667 | 91 | 2 | 223 | 1 | 3.31 | 6.50 | 3.10 | 5 | 1 |
| 668 | 91 | 2 | 223 | 2 | 8.59 | 9.50 | 5.30 | 5 | 1 |
| 669 | 91 | 2 | 223 | 3 | 5.35 | 7.00 | 4.50 | 5 | 1 |
| 670 | 92 | 2 | 224 | 1 | 14.64 | 11.50 | 8.00 | 4 | 1 |
| 671 | 92 | 2 | 224 | 2 | 15.98 | 12.00 | 8.00 | 4 | 1 |
| 672 | 92 | 2 | 224 | 3 | 17.92 | 12.50 | 8.50 | 5 | 1 |
| 673 | 93 | 2 | 225 | 1 | 6.46 | 9.00 | 6.00 | 5 | 1 |
| 674 | 93 | 2 | 225 | 2 | 12.06 | 10.50 | 8.00 | 5 | 1 |
| 675 | 93 | 2 | 225 | 3 | 14.42 | 11.00 | 7.50 | 5 | 1 |
| 676 | 94 | 2 | 226 | 1 | 6.96 | 10.50 | 2.00 | 4 | 1 |
| 677 | 94 | 2 | 226 | 2 | 0.00 | 0.00 | 0.00 | 0 | 0 |
| 678 | 94 | 2 | 226 | 3 | 8.85 | 10.30 | 5.50 | 5 | 1 |
| 679 | 95 | 2 | 227 | 1 | 5.98 | 7.00 | 5.00 | 5 | 1 |
| 680 | 95 | 2 | 227 | 2 | 4.42 | 6.50 | 4.00 | 5 | 1 |
| 681 | 95 | 2 | 227 | 3 | 9.29 | 9.50 | 6.00 | 4 | 1 |
| 682 | 96 | 2 | 228 | 1 | 7.26 | 6.50 | 5.00 | 5 | 1 |
| 683 | 96 | 2 | 228 | 2 | 3.57 | 5.00 | 4.50 | 5 | 1 |
| 684 | 96 | 2 | 228 | 3 | 5.86 | 5.50 | 4.50 | 5 | 1 |
| 685 | 97 | 2 | 229 | 1 | 2.16 | 4.00 | 3.00 | 5 | 1 |
| 686 | 97 | 2 | 229 | 2 | 5.12 | 5.50 | 4.00 | 5 | 1 |
| 687 | 97 | 2 | 229 | 3 | 8.34 | 7.50 | 5.00 | 5 | 1 |
| 688 | 98 | 2 | 230 | 1 | 6.37 | 5.00 | 3.20 | 5 | 1 |
| 689 | 98 | 2 | 230 | 2 | 3.02 | 3.30 | 2.30 | 3 | 1 |
| 690 | 98 | 2 | 230 | 3 | 3.31 | 4.00 | 3.50 | 4 | 1 |
| 691 | 99 | 2 | 231 | 1 | 4.42 | 4.60 | 3.60 | 5 | 1 |
| 692 | 99 | 2 | 231 | 2 | 8.31 | 7.00 | 5.00 | 5 | 1 |
| 693 | 99 | 2 | 231 | 3 | 5.51 | 5.50 | 4.00 | 4 | 1 |
| 694 | 100 | 2 | 232 | 1 | 14.93 | 13.00 | 8.50 | 4 | 1 |
| 695 | 100 | 2 | 232 | 2 | 12.83 | 12.00 | 8.50 | 4 | 1 |
| 696 | 100 | 2 | 232 | 3 | 11.24 | 10.00 | 6.50 | 5 | 1 |
| 697 | 101 | 2 | 233 | 1 | 6.08 | 6.90 | 5.70 | 5 | 1 |
| 698 | 101 | 2 | 233 | 2 | 6.43 | 7.20 | 5.70 | 5 | 1 |
| 699 | 101 | 2 | 233 | 3 | 0.00 | 0.00 | 0.00 | 0 | 0 |
| 700 | 102 | 2 | 234 | 1 | 4.97 | 4.70 | 3.30 | 5 | 1 |
| 701 | 102 | 2 | 234 | 2 | 0.00 | 0.00 | 0.00 | 0 | 0 |
| 702 | 102 | 2 | 234 | 3 | 6.46 | 5.00 | 4.00 | 5 | 1 |
| 703 | 103 | 2 | 235 | 1 | 11.94 | 10.30 | 7.00 | 5 | 1 |
| 704 | 103 | 2 | 235 | 2 | 10.98 | 10.50 | 7.30 | 4 | 1 |
| 705 | 103 | 2 | 235 | 3 | 9.87 | 10.20 | 6.50 | 4 | 1 |
| 706 | 104 | 2 | 236 | 1 | 11.01 | 10.80 | 7.00 | 4 | 1 |
| 707 | 104 | 2 | 236 | 2 | 14.80 | 12.30 | 8.00 | 5 | 1 |
| 708 | 104 | 2 | 236 | 3 | 11.14 | 10.30 | 7.00 | 5 | 1 |
| 709 | 105 | 2 | 237 | 1 | 5.35 | 5.30 | 3.00 | 4 | 1 |
| 710 | 105 | 2 | 237 | 2 | 9.61 | 9.50 | 5.00 | 5 | 1 |
| 711 | 105 | 2 | 237 | 3 | 8.12 | 9.20 | 4.50 | 5 | 1 |
| 712 | 106 | 2 | 238 | 1 | 6.40 | 5.50 | 4.20 | 4 | 1 |
| 713 | 106 | 2 | 238 | 2 | 7.93 | 7.00 | 3.60 | 5 | 1 |
| 714 | 106 | 2 | 238 | 3 | 5.54 | 5.80 | 4.80 | 4 | 1 |
| 715 | 107 | 2 | 239 | 1 | 10.54 | 10.50 | 8.00 | 5 | 1 |
| 716 | 107 | 2 | 239 | 2 | 10.76 | 10.50 | 7.50 | 5 | 1 |
| 717 | 107 | 2 | 239 | 3 | 9.29 | 10.00 | 8.00 | 4 | 1 |
| 718 | 108 | 2 | 240 | 1 | 4.52 | 4.00 | 3.00 | 3 | 1 |
| 719 | 108 | 2 | 240 | 2 | 2.07 | 3.60 | 3.20 | 5 | 1 |
| 720 | 108 | 2 | 240 | 3 | 5.83 | 4.50 | 3.60 | 5 | 1 |
| 721 | 109 | 2 | 241 | 1 | 3.88 | 3.80 | 3.30 | 4 | 1 |
| 722 | 109 | 2 | 241 | 2 | 4.42 | 4.10 | 3.60 | 4 | 1 |
| 723 | 109 | 2 | 241 | 3 | 4.74 | 4.50 | 3.50 | 4 | 1 |
| 724 | 110 | 2 | 242 | 1 | 0.00 | 0.00 | 0.00 | 0 | 0 |
| 725 | 110 | 2 | 242 | 2 | 14.93 | 12.50 | 9.00 | 5 | 1 |
| 726 | 110 | 2 | 242 | 3 | 14.26 | 12.50 | 8.50 | 4 | 1 |
| 727 | 111 | 2 | 243 | 1 | 9.45 | 9.30 | 7.50 | 5 | 1 |
| 728 | 111 | 2 | 243 | 2 | 0.00 | 0.00 | 0.00 | 0 | 0 |
| 729 | 111 | 2 | 243 | 3 | 5.03 | 6.70 | 5.50 | 5 | 1 |
| 730 | 112 | 2 | 244 | 1 | 6.30 | 5.50 | 4.00 | 5 | 1 |
| 731 | 112 | 2 | 244 | 2 | 4.07 | 3.80 | 2.80 | 5 | 1 |
| 732 | 112 | 2 | 244 | 3 | 6.53 | 4.20 | 3.60 | 5 | 1 |
| 733 | 113 | 2 | 245 | 1 | 5.79 | 8.00 | 5.00 | 5 | 1 |
| 734 | 113 | 2 | 245 | 2 | 1.97 | 2.80 | 2.50 | 4 | 1 |
| 735 | 113 | 2 | 245 | 3 | 0.00 | 0.00 | 0.00 | 0 | 0 |
| 736 | 114 | 2 | 246 | 1 | 8.53 | 9.00 | 7.00 | 5 | 1 |
| 737 | 114 | 2 | 246 | 2 | 10.92 | 9.30 | 7.00 | 5 | 1 |
| 738 | 114 | 2 | 246 | 3 | 5.60 | 7.50 | 5.50 | 5 | 1 |
| 739 | 115 | 2 | 247 | 1 | 4.17 | 5.10 | 4.20 | 5 | 1 |
| 740 | 115 | 2 | 247 | 2 | 4.62 | 5.50 | 4.50 | 5 | 1 |
| 741 | 115 | 2 | 247 | 3 | 3.41 | 3.50 | 2.70 | 4 | 1 |
| 742 | 116 | 2 | 248 | 1 | 8.79 | 13.00 | 9.00 | 5 | 1 |
| 743 | 116 | 2 | 248 | 2 | 11.20 | 12.80 | 6.50 | 5 | 1 |
| 744 | 116 | 2 | 248 | 3 | 6.11 | 7.80 | 5.50 | 4 | 1 |
| 745 | 117 | 2 | 249 | 1 | 5.57 | 6.00 | 5.00 | 5 | 1 |
| 746 | 117 | 2 | 249 | 2 | 0.00 | 0.00 | 0.00 | 0 | 0 |
| 747 | 117 | 2 | 249 | 3 | 7.45 | 8.00 | 6.00 | 5 | 1 |
| 748 | 118 | 2 | 250 | 1 | 5.67 | 7.50 | 4.00 | 5 | 1 |
| 749 | 118 | 2 | 250 | 2 | 4.65 | 6.20 | 4.00 | 5 | 1 |
| 750 | 118 | 2 | 250 | 3 | 2.04 | 4.50 | 2.50 | 3 | 1 |
| 751 | 119 | 2 | 251 | 1 | 9.23 | 10.30 | 4.80 | 5 | 1 |
| 752 | 119 | 2 | 251 | 2 | 7.13 | 9.20 | 5.00 | 5 | 1 |
| 753 | 119 | 2 | 251 | 3 | 8.12 | 9.70 | 5.00 | 5 | 1 |
| 754 | 120 | 2 | 252 | 1 | 12.67 | 9.60 | 6.50 | 4 | 1 |
| 755 | 120 | 2 | 252 | 2 | 9.49 | 9.00 | 6.00 | 4 | 1 |
| 756 | 120 | 2 | 252 | 3 | 10.70 | 9.30 | 5.50 | 5 | 1 |
| 757 | 121 | 2 | 253 | 1 | 3.85 | 4.00 | 3.50 | 4 | 1 |
| 758 | 121 | 2 | 253 | 2 | 5.41 | 6.00 | 5.00 | 5 | 1 |
| 759 | 121 | 2 | 253 | 3 | 2.04 | 4.00 | 3.00 | 4 | 1 |
| 760 | 122 | 2 | 254 | 1 | 11.71 | 12.00 | 8.50 | 5 | 1 |
| 761 | 122 | 2 | 254 | 2 | 7.58 | 9.00 | 7.00 | 4 | 1 |
| 762 | 122 | 2 | 254 | 3 | 14.32 | 12.50 | 7.00 | 5 | 1 |
| 763 | 123 | 2 | 255 | 1 | 5.67 | 7.50 | 4.00 | 5 | 1 |
| 764 | 123 | 2 | 255 | 2 | 11.71 | 11.50 | 8.50 | 5 | 1 |
| 765 | 123 | 2 | 255 | 3 | 8.24 | 10.00 | 7.00 | 5 | 1 |
| 766 | 124 | 2 | 256 | 1 | 11.24 | 10.50 | 7.00 | 5 | 1 |
| 767 | 124 | 2 | 256 | 2 | 8.79 | 9.00 | 6.50 | 4 | 1 |
| 768 | 124 | 2 | 256 | 3 | 14.10 | 11.50 | 8.00 | 5 | 1 |
| 769 | 125 | 2 | 257 | 1 | 11.55 | 10.80 | 4.80 | 5 | 1 |
| 770 | 125 | 2 | 257 | 2 | 8.66 | 10.20 | 5.00 | 5 | 1 |
| 771 | 125 | 2 | 257 | 3 | 9.07 | 10.00 | 5.00 | 5 | 1 |
| 772 | 126 | 2 | 258 | 1 | 16.42 | 12.00 | 9.00 | 4 | 1 |
| 773 | 126 | 2 | 258 | 2 | 13.18 | 11.50 | 8.50 | 5 | 1 |
| 774 | 126 | 2 | 258 | 3 | 14.16 | 11.50 | 8.00 | 5 | 1 |
| 775 | 127 | 2 | 259 | 1 | 9.52 | 9.00 | 6.00 | 4 | 1 |
| 776 | 127 | 2 | 259 | 2 | 8.34 | 9.30 | 7.00 | 5 | 1 |
| 777 | 127 | 2 | 259 | 3 | 10.66 | 9.50 | 7.00 | 5 | 1 |
| 778 | 128 | 2 | 260 | 1 | 5.22 | 4.00 | 3.00 | 3 | 1 |
| 779 | 128 | 2 | 260 | 2 | 3.57 | 4.00 | 3.50 | 4 | 1 |
| 780 | 128 | 2 | 260 | 3 | 4.65 | 4.50 | 3.70 | 3 | 1 |
| 781 | 129 | 2 | 261 | 1 | 14.48 | 12.00 | 8.00 | 5 | 1 |
| 782 | 129 | 2 | 261 | 2 | 7.32 | 11.00 | 8.50 | 5 | 1 |
| 783 | 129 | 2 | 261 | 3 | 11.55 | 11.00 | 2.50 | 4 | 1 |
| 784 | 130 | 2 | 262 | 1 | 9.45 | 12.00 | 7.00 | 5 | 1 |
| 785 | 130 | 2 | 262 | 2 | 9.23 | 11.50 | 6.50 | 5 | 1 |
| 786 | 130 | 2 | 262 | 3 | 6.72 | 11.00 | 7.50 | 5 | 1 |
| 787 | 131 | 2 | 263 | 1 | 5.51 | 5.10 | 3.50 | 5 | 1 |
| 788 | 131 | 2 | 263 | 2 | 6.46 | 5.00 | 3.50 | 5 | 1 |
| 789 | 131 | 2 | 263 | 3 | 7.70 | 6.50 | 5.00 | 4 | 1 |
| 790 | 132 | 2 | 264 | 1 | 8.91 | 12.00 | 5.00 | 5 | 1 |
| 791 | 132 | 2 | 264 | 2 | 0.00 | 0.00 | 0.00 | 0 | 0 |
| 792 | 132 | 2 | 264 | 3 | 5.41 | 11.00 | 7.00 | 5 | 1 |
| 793 | 1 | 3 | 265 | 1 | 5.09 | 5.00 | 4.00 | 5 | 1 |
| 794 | 1 | 3 | 265 | 2 | 3.82 | 4.50 | 4.00 | 5 | 1 |
| 795 | 1 | 3 | 265 | 3 | 3.50 | 3.80 | 3.00 | 5 | 1 |
| 796 | 2 | 3 | 266 | 1 | 10.19 | 10.50 | 8.50 | 5 | 1 |
| 797 | 2 | 3 | 266 | 2 | 9.71 | 12.00 | 10.00 | 4 | 1 |
| 798 | 2 | 3 | 266 | 3 | 3.66 | 5.00 | 4.50 | 5 | 1 |
| 799 | 3 | 3 | 267 | 1 | 13.78 | 12.00 | 5.00 | 5 | 1 |
| 800 | 3 | 3 | 267 | 2 | 14.01 | 7.00 | 7.00 | 5 | 1 |
| 801 | 3 | 3 | 267 | 3 | 1.59 | 3.00 | 1.50 | 5 | 1 |
| 802 | 4 | 3 | 268 | 1 | 11.01 | 9.00 | 7.00 | 5 | 1 |
| 803 | 4 | 3 | 268 | 2 | 5.60 | 6.50 | 4.50 | 4 | 1 |
| 804 | 4 | 3 | 268 | 3 | 7.00 | 7.00 | 5.00 | 4 | 1 |
| 805 | 5 | 3 | 269 | 1 | 4.93 | 5.00 | 4.00 | 4 | 1 |
| 806 | 5 | 3 | 269 | 2 | 2.55 | 4.00 | 3.00 | 4 | 1 |
| 807 | 5 | 3 | 269 | 3 | 6.14 | 6.00 | 4.00 | 4 | 1 |
| 808 | 6 | 3 | 270 | 1 | 2.39 | 3.50 | 2.00 | 5 | 1 |
| 809 | 6 | 3 | 270 | 2 | 6.53 | 8.00 | 6.50 | 5 | 1 |
| 810 | 6 | 3 | 270 | 3 | 14.10 | 13.50 | 11.00 | 5 | 1 |
| 811 | 7 | 3 | 271 | 1 | 14.01 | 14.00 | 8.00 | 5 | 1 |
| 812 | 7 | 3 | 271 | 2 | 8.59 | 9.00 | 8.00 | 5 | 1 |
| 813 | 7 | 3 | 271 | 3 | 0.00 | 0.00 | 0.00 | 0 | 0 |
| 814 | 8 | 3 | 272 | 1 | 11.94 | 9.00 | 8.00 | 4 | 1 |
| 815 | 8 | 3 | 272 | 2 | 15.92 | 11.00 | 9.00 | 5 | 1 |
| 816 | 8 | 3 | 272 | 3 | 0.00 | 0.00 | 0.00 | 0 | 0 |
| 817 | 9 | 3 | 273 | 1 | 16.71 | 12.00 | 9.00 | 5 | 1 |
| 818 | 9 | 3 | 273 | 2 | 9.42 | 10.00 | 9.00 | 5 | 1 |
| 819 | 9 | 3 | 273 | 3 | 6.68 | 9.00 | 8.00 | 5 | 1 |
| 820 | 10 | 3 | 274 | 1 | 5.86 | 6.20 | 5.20 | 5 | 1 |
| 821 | 10 | 3 | 274 | 2 | 5.51 | 7.50 | 6.50 | 5 | 1 |
| 822 | 10 | 3 | 274 | 3 | 6.21 | 7.00 | 6.00 | 5 | 1 |
| 823 | 11 | 3 | 275 | 1 | 10.19 | 10.00 | 9.00 | 4 | 1 |
| 824 | 11 | 3 | 275 | 2 | 5.41 | 4.00 | 3.00 | 5 | 1 |
| 825 | 11 | 3 | 275 | 3 | 14.01 | 12.00 | 10.00 | 5 | 1 |
| 826 | 12 | 3 | 276 | 1 | 0.00 | 0.00 | 0.00 | 0 | 0 |
| 827 | 12 | 3 | 276 | 2 | 9.04 | 9.00 | 7.00 | 4 | 1 |
| 828 | 12 | 3 | 276 | 3 | 0.00 | 0.00 | 0.00 | 0 | 0 |
| 829 | 13 | 3 | 277 | 1 | 14.99 | 12.00 | 10.00 | 5 | 1 |
| 830 | 13 | 3 | 277 | 2 | 8.82 | 11.00 | 10.00 | 5 | 1 |
| 831 | 13 | 3 | 277 | 3 | 11.24 | 11.50 | 9.50 | 5 | 1 |
| 832 | 14 | 3 | 278 | 1 | 0.00 | 0.00 | 0.00 | 0 | 0 |
| 833 | 14 | 3 | 278 | 2 | 8.59 | 10.00 | 8.00 | 5 | 1 |
| 834 | 14 | 3 | 278 | 3 | 4.93 | 8.00 | 7.00 | 5 | 1 |
| 835 | 15 | 3 | 279 | 1 | 0.00 | 0.00 | 0.00 | 0 | 0 |
| 836 | 15 | 3 | 279 | 2 | 14.07 | 12.00 | 8.00 | 5 | 1 |
| 837 | 15 | 3 | 279 | 3 | 5.47 | 7.00 | 5.00 | 5 | 1 |
| 838 | 16 | 3 | 280 | 1 | 7.00 | 9.00 | 8.00 | 4 | 1 |
| 839 | 16 | 3 | 280 | 2 | 7.54 | 8.00 | 7.00 | 5 | 1 |
| 840 | 16 | 3 | 280 | 3 | 13.37 | 11.00 | 9.00 | 5 | 1 |
| 841 | 17 | 3 | 281 | 1 | 7.42 | 8.00 | 7.00 | 4 | 1 |
| 842 | 17 | 3 | 281 | 2 | 0.00 | 0.00 | 0.00 | 0 | 0 |
| 843 | 17 | 3 | 281 | 3 | 7.64 | 7.50 | 5.50 | 5 | 1 |
| 844 | 18 | 3 | 282 | 1 | 6.53 | 8.00 | 6.00 | 4 | 1 |
| 845 | 18 | 3 | 282 | 2 | 9.39 | 8.20 | 6.20 | 5 | 1 |
| 846 | 18 | 3 | 282 | 3 | 13.46 | 10.20 | 7.20 | 5 | 1 |
| 847 | 19 | 3 | 283 | 1 | 10.19 | 8.00 | 6.00 | 5 | 1 |
| 848 | 19 | 3 | 283 | 2 | 7.38 | 7.00 | 6.00 | 5 | 1 |
| 849 | 19 | 3 | 283 | 3 | 9.23 | 7.50 | 6.50 | 4 | 1 |
| 850 | 20 | 3 | 284 | 1 | 14.01 | 12.00 | 8.00 | 5 | 1 |
| 851 | 20 | 3 | 284 | 2 | 8.59 | 9.00 | 7.00 | 5 | 1 |
| 852 | 20 | 3 | 284 | 3 | 3.18 | 6.00 | 5.00 | 5 | 1 |
| 853 | 21 | 3 | 285 | 1 | 0.00 | 0.00 | 0.00 | 0 | 0 |
| 854 | 21 | 3 | 285 | 2 | 7.70 | 10.00 | 9.00 | 5 | 1 |
| 855 | 21 | 3 | 285 | 3 | 11.30 | 9.00 | 7.00 | 5 | 1 |
| 856 | 22 | 3 | 286 | 1 | 8.24 | 10.00 | 7.00 | 5 | 1 |
| 857 | 22 | 3 | 286 | 2 | 8.79 | 8.00 | 7.00 | 5 | 1 |
| 858 | 22 | 3 | 286 | 3 | 9.20 | 9.00 | 7.00 | 4 | 1 |
| 859 | 23 | 3 | 287 | 1 | 9.71 | 7.00 | 5.00 | 5 | 1 |
| 860 | 23 | 3 | 287 | 2 | 10.82 | 8.00 | 7.00 | 4 | 1 |
| 861 | 23 | 3 | 287 | 3 | 10.82 | 7.50 | 5.50 | 5 | 1 |
| 862 | 24 | 3 | 288 | 1 | 11.14 | 8.00 | 6.00 | 5 | 1 |
| 863 | 24 | 3 | 288 | 2 | 12.41 | 9.50 | 9.00 | 4 | 1 |
| 864 | 24 | 3 | 288 | 3 | 3.66 | 6.00 | 5.00 | 4 | 1 |
| 865 | 25 | 3 | 289 | 1 | 8.91 | 8.00 | 6.50 | 4 | 1 |
| 866 | 25 | 3 | 289 | 2 | 2.86 | 6.00 | 5.00 | 5 | 1 |
| 867 | 25 | 3 | 289 | 3 | 2.83 | 6.50 | 5.50 | 5 | 1 |
| 868 | 26 | 3 | 290 | 1 | 8.28 | 10.50 | 4.00 | 5 | 1 |
| 869 | 26 | 3 | 290 | 2 | 13.46 | 12.00 | 10.00 | 4 | 1 |
| 870 | 26 | 3 | 290 | 3 | 12.57 | 10.00 | 8.00 | 4 | 1 |
| 871 | 27 | 3 | 291 | 1 | 12.25 | 12.00 | 9.00 | 5 | 1 |
| 872 | 27 | 3 | 291 | 2 | 13.02 | 13.00 | 10.00 | 5 | 1 |
| 873 | 27 | 3 | 291 | 3 | 6.53 | 8.00 | 6.00 | 4 | 1 |
| 874 | 28 | 3 | 292 | 1 | 9.64 | 10.50 | 9.00 | 4 | 1 |
| 875 | 28 | 3 | 292 | 2 | 10.09 | 12.00 | 10.00 | 5 | 1 |
| 876 | 28 | 3 | 292 | 3 | 8.59 | 9.50 | 7.50 | 4 | 1 |
| 877 | 29 | 3 | 293 | 1 | 11.46 | 11.00 | 10.00 | 5 | 1 |
| 878 | 29 | 3 | 293 | 2 | 10.35 | 10.00 | 8.00 | 5 | 1 |
| 879 | 29 | 3 | 293 | 3 | 12.57 | 10.50 | 8.50 | 5 | 1 |
| 880 | 30 | 3 | 294 | 1 | 8.12 | 8.00 | 6.50 | 5 | 1 |
| 881 | 30 | 3 | 294 | 2 | 4.14 | 6.00 | 5.50 | 4 | 1 |
| 882 | 30 | 3 | 294 | 3 | 8.91 | 8.00 | 6.00 | 5 | 1 |
| 883 | 31 | 3 | 295 | 1 | 11.71 | 11.00 | 9.00 | 5 | 1 |
| 884 | 31 | 3 | 295 | 2 | 7.64 | 9.00 | 6.00 | 5 | 1 |
| 885 | 31 | 3 | 295 | 3 | 11.46 | 12.00 | 10.00 | 5 | 1 |
| 886 | 32 | 3 | 296 | 1 | 11.78 | 10.00 | 8.00 | 4 | 1 |
| 887 | 32 | 3 | 296 | 2 | 11.14 | 9.00 | 7.00 | 5 | 1 |
| 888 | 32 | 3 | 296 | 3 | 8.31 | 11.00 | 9.00 | 5 | 1 |
| 889 | 33 | 3 | 297 | 1 | 9.52 | 9.00 | 7.20 | 5 | 1 |
| 890 | 33 | 3 | 297 | 2 | 12.10 | 10.00 | 8.00 | 5 | 1 |
| 891 | 33 | 3 | 297 | 3 | 14.29 | 9.50 | 7.50 | 5 | 1 |
| 892 | 34 | 3 | 298 | 1 | 12.10 | 12.00 | 10.00 | 5 | 1 |
| 893 | 34 | 3 | 298 | 2 | 8.59 | 8.00 | 7.00 | 2 | 1 |
| 894 | 34 | 3 | 298 | 3 | 13.24 | 10.00 | 7.00 | 5 | 1 |
| 895 | 35 | 3 | 299 | 1 | 13.66 | 10.00 | 8.50 | 4 | 1 |
| 896 | 35 | 3 | 299 | 2 | 3.28 | 6.00 | 5.00 | 5 | 1 |
| 897 | 35 | 3 | 299 | 3 | 4.93 | 7.00 | 6.00 | 4 | 1 |
| 898 | 36 | 3 | 300 | 1 | 0.00 | 0.00 | 0.00 | 0 | 0 |
| 899 | 36 | 3 | 300 | 2 | 6.84 | 9.00 | 7.00 | 5 | 1 |
| 900 | 36 | 3 | 300 | 3 | 12.73 | 12.00 | 11.00 | 5 | 1 |
| 901 | 37 | 3 | 301 | 1 | 11.46 | 12.00 | 10.00 | 5 | 1 |
| 902 | 37 | 3 | 301 | 2 | 10.35 | 11.50 | 10.50 | 5 | 1 |
| 903 | 37 | 3 | 301 | 3 | 12.83 | 12.00 | 10.00 | 5 | 1 |
| 904 | 38 | 3 | 302 | 1 | 10.98 | 11.00 | 9.00 | 5 | 1 |
| 905 | 38 | 3 | 302 | 2 | 10.92 | 12.00 | 10.00 | 4 | 1 |
| 906 | 38 | 3 | 302 | 3 | 5.25 | 8.00 | 7.00 | 4 | 1 |
| 907 | 39 | 3 | 303 | 1 | 14.16 | 12.00 | 8.00 | 5 | 1 |
| 908 | 39 | 3 | 303 | 2 | 0.00 | 0.00 | 0.00 | 0 | 0 |
| 909 | 39 | 3 | 303 | 3 | 9.23 | 10.00 | 8.00 | 5 | 1 |
| 910 | 40 | 3 | 304 | 1 | 12.41 | 12.00 | 9.00 | 5 | 1 |
| 911 | 40 | 3 | 304 | 2 | 13.53 | 12.50 | 10.50 | 4 | 1 |
| 912 | 40 | 3 | 304 | 3 | 13.69 | 11.00 | 9.00 | 5 | 1 |
| 913 | 41 | 3 | 305 | 1 | 5.98 | 7.00 | 6.00 | 5 | 1 |
| 914 | 41 | 3 | 305 | 2 | 5.73 | 7.50 | 6.50 | 5 | 1 |
| 915 | 41 | 3 | 305 | 3 | 5.57 | 7.80 | 6.80 | 5 | 1 |
| 916 | 42 | 3 | 306 | 1 | 3.12 | 6.00 | 5.00 | 4 | 1 |
| 917 | 42 | 3 | 306 | 2 | 6.84 | 8.00 | 6.00 | 5 | 1 |
| 918 | 42 | 3 | 306 | 3 | 13.21 | 10.00 | 8.00 | 5 | 1 |
| 919 | 43 | 3 | 307 | 1 | 6.02 | 7.00 | 6.00 | 5 | 1 |
| 920 | 43 | 3 | 307 | 2 | 14.67 | 12.00 | 9.00 | 5 | 1 |
| 921 | 43 | 3 | 307 | 3 | 17.35 | 11.00 | 9.00 | 4 | 1 |
| 922 | 44 | 3 | 308 | 1 | 12.41 | 12.00 | 9.00 | 4 | 1 |
| 923 | 44 | 3 | 308 | 2 | 11.62 | 10.00 | 8.00 | 4 | 1 |
| 924 | 44 | 3 | 308 | 3 | 11.49 | 11.00 | 9.00 | 5 | 1 |
| 925 | 45 | 3 | 309 | 1 | 6.84 | 8.50 | 7.50 | 5 | 1 |
| 926 | 45 | 3 | 309 | 2 | 10.19 | 11.00 | 9.00 | 5 | 1 |
| 927 | 45 | 3 | 309 | 3 | 0.00 | 0.00 | 0.00 | 0 | 0 |
| 928 | 46 | 3 | 310 | 1 | 6.37 | 7.50 | 7.00 | 4 | 1 |
| 929 | 46 | 3 | 310 | 2 | 10.06 | 8.00 | 6.00 | 4 | 1 |
| 930 | 46 | 3 | 310 | 3 | 3.50 | 6.00 | 5.00 | 4 | 1 |
| 931 | 47 | 3 | 311 | 1 | 12.41 | 12.00 | 10.00 | 4 | 1 |
| 932 | 47 | 3 | 311 | 2 | 12.67 | 13.00 | 11.00 | 4 | 1 |
| 933 | 47 | 3 | 311 | 3 | 10.82 | 10.00 | 8.00 | 5 | 1 |
| 934 | 48 | 3 | 312 | 1 | 16.39 | 15.00 | 13.00 | 5 | 1 |
| 935 | 48 | 3 | 312 | 2 | 15.76 | 12.00 | 10.00 | 5 | 1 |
| 936 | 48 | 3 | 312 | 3 | 16.07 | 11.00 | 10.00 | 4 | 1 |
| 937 | 49 | 3 | 313 | 1 | 6.05 | 6.00 | 4.00 | 5 | 1 |
| 938 | 49 | 3 | 313 | 2 | 5.09 | 6.10 | 3.00 | 5 | 1 |
| 939 | 49 | 3 | 313 | 3 | 4.46 | 6.30 | 6.00 | 5 | 1 |
| 940 | 50 | 3 | 314 | 1 | 11.62 | 12.00 | 9.00 | 5 | 1 |
| 941 | 50 | 3 | 314 | 2 | 12.86 | 11.00 | 8.00 | 5 | 1 |
| 942 | 50 | 3 | 314 | 3 | 12.51 | 9.00 | 7.00 | 5 | 1 |
| 943 | 51 | 3 | 315 | 1 | 0.00 | 0.00 | 0.00 | 0 | 0 |
| 944 | 51 | 3 | 315 | 2 | 9.10 | 9.00 | 7.00 | 5 | 1 |
| 945 | 51 | 3 | 315 | 3 | 8.12 | 7.50 | 6.50 | 4 | 1 |
| 946 | 52 | 3 | 316 | 1 | 9.39 | 9.20 | 8.20 | 4 | 1 |
| 947 | 52 | 3 | 316 | 2 | 11.43 | 10.00 | 8.00 | 5 | 1 |
| 948 | 52 | 3 | 316 | 3 | 7.64 | 8.50 | 7.50 | 5 | 1 |
| 949 | 53 | 3 | 317 | 1 | 6.05 | 8.00 | 7.00 | 4 | 1 |
| 950 | 53 | 3 | 317 | 2 | 8.37 | 10.00 | 9.00 | 4 | 1 |
| 951 | 53 | 3 | 317 | 3 | 3.02 | 4.00 | 2.60 | 4 | 1 |
| 952 | 54 | 3 | 318 | 1 | 10.19 | 8.00 | 4.00 | 5 | 1 |
| 953 | 54 | 3 | 318 | 2 | 0.00 | 0.00 | 0.00 | 0 | 0 |
| 954 | 54 | 3 | 318 | 3 | 8.28 | 7.00 | 6.00 | 5 | 1 |
| 955 | 55 | 3 | 319 | 1 | 11.62 | 12.00 | 10.00 | 4 | 1 |
| 956 | 55 | 3 | 319 | 2 | 11.55 | 11.00 | 6.00 | 5 | 1 |
| 957 | 55 | 3 | 319 | 3 | 10.03 | 11.00 | 10.00 | 5 | 1 |
| 958 | 56 | 3 | 320 | 1 | 0.00 | 0.00 | 0.00 | 0 | 0 |
| 959 | 56 | 3 | 320 | 2 | 6.75 | 10.00 | 9.00 | 5 | 1 |
| 960 | 56 | 3 | 320 | 3 | 13.18 | 12.00 | 10.00 | 5 | 1 |
| 961 | 57 | 3 | 321 | 1 | 10.35 | 10.00 | 8.00 | 5 | 1 |
| 962 | 57 | 3 | 321 | 2 | 2.07 | 3.00 | 2.00 | 5 | 1 |
| 963 | 57 | 3 | 321 | 3 | 8.59 | 11.00 | 8.00 | 5 | 1 |
| 964 | 58 | 3 | 322 | 1 | 9.52 | 10.00 | 8.00 | 5 | 1 |
| 965 | 58 | 3 | 322 | 2 | 9.55 | 9.00 | 8.00 | 4 | 1 |
| 966 | 58 | 3 | 322 | 3 | 14.93 | 11.00 | 9.00 | 5 | 1 |
| 967 | 59 | 3 | 323 | 1 | 2.23 | 4.00 | 3.00 | 5 | 1 |
| 968 | 59 | 3 | 323 | 2 | 11.94 | 10.00 | 8.00 | 4 | 1 |
| 969 | 59 | 3 | 323 | 3 | 6.65 | 8.00 | 6.00 | 5 | 1 |
| 970 | 60 | 3 | 324 | 1 | 5.89 | 7.00 | 2.50 | 5 | 1 |
| 971 | 60 | 3 | 324 | 2 | 5.86 | 7.50 | 5.00 | 4 | 1 |
| 972 | 60 | 3 | 324 | 3 | 5.57 | 6.50 | 2.50 | 5 | 1 |
| 973 | 61 | 3 | 325 | 1 | 12.99 | 11.00 | 9.00 | 5 | 1 |
| 974 | 61 | 3 | 325 | 2 | 9.07 | 9.00 | 7.00 | 5 | 1 |
| 975 | 61 | 3 | 325 | 3 | 11.33 | 12.00 | 9.00 | 5 | 1 |
| 976 | 62 | 3 | 326 | 1 | 5.67 | 6.50 | 5.50 | 4 | 1 |
| 977 | 62 | 3 | 326 | 2 | 2.86 | 5.00 | 4.50 | 5 | 1 |
| 978 | 62 | 3 | 326 | 3 | 5.09 | 7.00 | 6.00 | 4 | 1 |
| 979 | 63 | 3 | 327 | 1 | 1.43 | 3.00 | 1.00 | 5 | 1 |
| 980 | 63 | 3 | 327 | 2 | 7.73 | 9.00 | 8.00 | 5 | 1 |
| 981 | 63 | 3 | 327 | 3 | 6.94 | 8.00 | 7.00 | 5 | 1 |
| 982 | 64 | 3 | 328 | 1 | 9.39 | 10.00 | 8.00 | 5 | 1 |
| 983 | 64 | 3 | 328 | 2 | 12.80 | 10.00 | 9.00 | 5 | 1 |
| 984 | 64 | 3 | 328 | 3 | 0.00 | 0.00 | 0.00 | 0 | 0 |
| 985 | 65 | 3 | 329 | 1 | 0.00 | 0.00 | 0.00 | 0 | 0 |
| 986 | 65 | 3 | 329 | 2 | 3.82 | 6.00 | 2.00 | 4 | 1 |
| 987 | 65 | 3 | 329 | 3 | 4.14 | 7.00 | 2.00 | 5 | 1 |
| 988 | 66 | 3 | 330 | 1 | 6.21 | 6.50 | 5.00 | 4 | 1 |
| 989 | 66 | 3 | 330 | 2 | 4.14 | 6.00 | 5.00 | 4 | 1 |
| 990 | 66 | 3 | 330 | 3 | 5.57 | 6.50 | 6.00 | 4 | 1 |
| 991 | 67 | 3 | 331 | 1 | 10.66 | 8.00 | 6.00 | 5 | 1 |
| 992 | 67 | 3 | 331 | 2 | 3.76 | 6.00 | 5.00 | 4 | 1 |
| 993 | 67 | 3 | 331 | 3 | 4.07 | 6.50 | 6.00 | 4 | 1 |
| 994 | 68 | 3 | 332 | 1 | 1.67 | 6.00 | 4.00 | 4 | 1 |
| 995 | 68 | 3 | 332 | 2 | 9.71 | 11.00 | 10.00 | 5 | 1 |
| 996 | 68 | 3 | 332 | 3 | 8.28 | 10.50 | 8.50 | 5 | 1 |
| 997 | 69 | 3 | 333 | 1 | 5.25 | 7.00 | 3.00 | 4 | 1 |
| 998 | 69 | 3 | 333 | 2 | 4.33 | 7.50 | 5.50 | 5 | 1 |
| 999 | 69 | 3 | 333 | 3 | 1.91 | 4.00 | 3.00 | 5 | 1 |
| 1000 | 70 | 3 | 334 | 1 | 5.57 | 7.50 | 6.50 | 4 | 1 |
| 1001 | 70 | 3 | 334 | 2 | 12.22 | 12.00 | 9.00 | 4 | 1 |
| 1002 | 70 | 3 | 334 | 3 | 9.39 | 9.00 | 7.00 | 5 | 1 |
| 1003 | 71 | 3 | 335 | 1 | 4.93 | 4.00 | 3.00 | 5 | 1 |
| 1004 | 71 | 3 | 335 | 2 | 10.50 | 9.00 | 7.00 | 5 | 1 |
| 1005 | 71 | 3 | 335 | 3 | 14.29 | 11.00 | 9.00 | 5 | 1 |
| 1006 | 72 | 3 | 336 | 1 | 6.68 | 8.00 | 7.00 | 5 | 1 |
| 1007 | 72 | 3 | 336 | 2 | 6.84 | 7.50 | 7.00 | 5 | 1 |
| 1008 | 72 | 3 | 336 | 3 | 5.57 | 6.00 | 5.00 | 5 | 1 |
| 1009 | 73 | 3 | 337 | 1 | 7.64 | 8.50 | 7.00 | 5 | 1 |
| 1010 | 73 | 3 | 337 | 2 | 7.00 | 8.00 | 6.00 | 5 | 1 |
| 1011 | 73 | 3 | 337 | 3 | 3.98 | 6.00 | 5.00 | 5 | 1 |
| 1012 | 74 | 3 | 338 | 1 | 13.53 | 11.00 | 9.00 | 5 | 1 |
| 1013 | 74 | 3 | 338 | 2 | 14.96 | 12.00 | 11.00 | 5 | 1 |
| 1014 | 74 | 3 | 338 | 3 | 8.75 | 9.00 | 8.00 | 5 | 1 |
| 1015 | 75 | 3 | 339 | 1 | 19.48 | 13.00 | 10.00 | 5 | 1 |
| 1016 | 75 | 3 | 339 | 2 | 14.80 | 11.00 | 9.00 | 5 | 1 |
| 1017 | 75 | 3 | 339 | 3 | 10.44 | 9.00 | 8.00 | 5 | 1 |
| 1018 | 76 | 3 | 340 | 1 | 6.78 | 9.00 | 8.00 | 5 | 1 |
| 1019 | 76 | 3 | 340 | 2 | 6.46 | 8.00 | 7.00 | 5 | 1 |
| 1020 | 76 | 3 | 340 | 3 | 11.20 | 10.00 | 7.00 | 5 | 1 |
| 1021 | 77 | 3 | 341 | 1 | 8.37 | 8.50 | 2.00 | 4 | 1 |
| 1022 | 77 | 3 | 341 | 2 | 1.75 | 3.00 | 2.00 | 4 | 1 |
| 1023 | 77 | 3 | 341 | 3 | 0.95 | 2.00 | 1.00 | 5 | 1 |
| 1024 | 78 | 3 | 342 | 1 | 15.44 | 9.00 | 6.00 | 4 | 1 |
| 1025 | 78 | 3 | 342 | 2 | 12.73 | 10.00 | 8.00 | 4 | 1 |
| 1026 | 78 | 3 | 342 | 3 | 14.01 | 6.00 | 4.00 | 4 | 1 |
| 1027 | 79 | 3 | 343 | 1 | 8.75 | 10.00 | 8.00 | 5 | 1 |
| 1028 | 79 | 3 | 343 | 2 | 9.71 | 9.50 | 7.50 | 5 | 1 |
| 1029 | 79 | 3 | 343 | 3 | 9.55 | 8.50 | 6.50 | 5 | 1 |
| 1030 | 80 | 3 | 344 | 1 | 4.46 | 8.00 | 7.00 | 4 | 1 |
| 1031 | 80 | 3 | 344 | 2 | 8.12 | 7.00 | 6.00 | 5 | 1 |
| 1032 | 80 | 3 | 344 | 3 | 14.61 | 10.00 | 8.00 | 5 | 1 |
| 1033 | 81 | 3 | 345 | 1 | 9.64 | 9.00 | 7.00 | 5 | 1 |
| 1034 | 81 | 3 | 345 | 2 | 9.39 | 8.50 | 7.50 | 5 | 1 |
| 1035 | 81 | 3 | 345 | 3 | 5.41 | 6.00 | 4.00 | 5 | 1 |
| 1036 | 82 | 3 | 346 | 1 | 8.34 | 9.00 | 7.00 | 4 | 1 |
| 1037 | 82 | 3 | 346 | 2 | 10.19 | 9.50 | 8.50 | 4 | 1 |
| 1038 | 82 | 3 | 346 | 3 | 10.66 | 10.00 | 9.00 | 5 | 1 |
| 1039 | 83 | 3 | 347 | 1 | 8.34 | 8.00 | 6.00 | 4 | 1 |
| 1040 | 83 | 3 | 347 | 2 | 5.92 | 6.50 | 5.50 | 4 | 1 |
| 1041 | 83 | 3 | 347 | 3 | 11.01 | 8.50 | 6.50 | 5 | 1 |
| 1042 | 84 | 3 | 348 | 1 | 8.91 | 10.50 | 8.50 | 5 | 1 |
| 1043 | 84 | 3 | 348 | 2 | 10.19 | 10.00 | 8.00 | 5 | 1 |
| 1044 | 84 | 3 | 348 | 3 | 6.21 | 9.00 | 8.00 | 5 | 1 |
| 1045 | 85 | 3 | 349 | 1 | 7.96 | 10.00 | 6.00 | 4 | 1 |
| 1046 | 85 | 3 | 349 | 2 | 6.27 | 8.50 | 6.50 | 4 | 1 |
| 1047 | 85 | 3 | 349 | 3 | 5.73 | 8.00 | 7.00 | 5 | 1 |
| 1048 | 86 | 3 | 350 | 1 | 15.44 | 14.00 | 9.00 | 5 | 1 |
| 1049 | 86 | 3 | 350 | 2 | 8.05 | 9.50 | 8.50 | 5 | 1 |
| 1050 | 86 | 3 | 350 | 3 | 13.15 | 12.00 | 10.00 | 5 | 1 |
| 1051 | 87 | 3 | 351 | 1 | 7.96 | 9.50 | 7.50 | 5 | 1 |
| 1052 | 87 | 3 | 351 | 2 | 9.61 | 10.00 | 7.00 | 5 | 1 |
| 1053 | 87 | 3 | 351 | 3 | 10.57 | 9.00 | 7.00 | 5 | 1 |
| 1054 | 88 | 3 | 352 | 1 | 4.42 | 5.50 | 3.50 | 4 | 1 |
| 1055 | 88 | 3 | 352 | 2 | 5.25 | 6.00 | 4.00 | 4 | 1 |
| 1056 | 88 | 3 | 352 | 3 | 3.66 | 5.50 | 4.50 | 4 | 1 |
| 1057 | 89 | 3 | 353 | 1 | 8.80 | 10.00 | 9.00 | 4 | 1 |
| 1058 | 89 | 3 | 353 | 2 | 12.32 | 13.00 | 9.00 | 5 | 1 |
| 1059 | 89 | 3 | 353 | 3 | 13.31 | 12.00 | 10.00 | 4 | 1 |
| 1060 | 90 | 3 | 354 | 1 | 12.73 | 11.00 | 10.00 | 5 | 1 |
| 1061 | 90 | 3 | 354 | 2 | 6.84 | 9.00 | 8.00 | 4 | 1 |
| 1062 | 90 | 3 | 354 | 3 | 14.96 | 12.00 | 10.00 | 5 | 1 |
| 1063 | 91 | 3 | 355 | 1 | 12.73 | 10.00 | 8.00 | 4 | 1 |
| 1064 | 91 | 3 | 355 | 2 | 11.94 | 9.00 | 7.00 | 4 | 1 |
| 1065 | 91 | 3 | 355 | 3 | 16.30 | 8.50 | 6.50 | 5 | 1 |
| 1066 | 92 | 3 | 356 | 1 | 14.64 | 12.00 | 9.00 | 5 | 1 |
| 1067 | 92 | 3 | 356 | 2 | 9.07 | 11.00 | 9.00 | 4 | 1 |
| 1068 | 92 | 3 | 356 | 3 | 12.70 | 12.00 | 9.00 | 5 | 1 |
| 1069 | 93 | 3 | 357 | 1 | 9.01 | 10.00 | 8.00 | 5 | 1 |
| 1070 | 93 | 3 | 357 | 2 | 7.00 | 8.00 | 7.00 | 5 | 1 |
| 1071 | 93 | 3 | 357 | 3 | 10.09 | 10.00 | 9.00 | 5 | 1 |
| 1072 | 94 | 3 | 358 | 1 | 5.54 | 6.50 | 5.50 | 5 | 1 |
| 1073 | 94 | 3 | 358 | 2 | 6.53 | 7.00 | 4.00 | 5 | 1 |
| 1074 | 94 | 3 | 358 | 3 | 4.14 | 5.50 | 5.00 | 5 | 1 |
| 1075 | 95 | 3 | 359 | 1 | 9.71 | 10.00 | 9.00 | 5 | 1 |
| 1076 | 95 | 3 | 359 | 2 | 11.33 | 11.00 | 9.00 | 5 | 1 |
| 1077 | 95 | 3 | 359 | 3 | 9.04 | 8.00 | 6.00 | 5 | 1 |
| 1078 | 96 | 3 | 360 | 1 | 9.26 | 9.00 | 6.00 | 4 | 1 |
| 1079 | 96 | 3 | 360 | 2 | 4.46 | 7.00 | 6.00 | 4 | 1 |
| 1080 | 96 | 3 | 360 | 3 | 7.29 | 9.00 | 8.00 | 5 | 1 |
| 1081 | 97 | 3 | 361 | 1 | 10.95 | 11.00 | 10.00 | 4 | 1 |
| 1082 | 97 | 3 | 361 | 2 | 7.58 | 9.00 | 7.00 | 4 | 1 |
| 1083 | 97 | 3 | 361 | 3 | 11.59 | 12.00 | 11.00 | 4 | 1 |
| 1084 | 98 | 3 | 362 | 1 | 10.50 | 11.00 | 9.00 | 5 | 1 |
| 1085 | 98 | 3 | 362 | 2 | 12.45 | 11.00 | 6.00 | 5 | 1 |
| 1086 | 98 | 3 | 362 | 3 | 17.19 | 12.00 | 8.00 | 5 | 1 |
| 1087 | 99 | 3 | 363 | 1 | 9.64 | 7.80 | 4.80 | 5 | 1 |
| 1088 | 99 | 3 | 363 | 2 | 7.42 | 7.00 | 5.00 | 5 | 1 |
| 1089 | 99 | 3 | 363 | 3 | 0.00 | 0.00 | 0.00 | 0 | 0 |
| 1090 | 100 | 3 | 364 | 1 | 7.89 | 9.50 | 6.50 | 5 | 1 |
| 1091 | 100 | 3 | 364 | 2 | 4.93 | 8.00 | 7.00 | 5 | 1 |
| 1092 | 100 | 3 | 364 | 3 | 12.10 | 12.00 | 9.00 | 5 | 1 |
| 1093 | 101 | 3 | 365 | 1 | 6.94 | 8.00 | 6.00 | 4 | 1 |
| 1094 | 101 | 3 | 365 | 2 | 7.96 | 8.50 | 2.60 | 4 | 1 |
| 1095 | 101 | 3 | 365 | 3 | 5.25 | 6.00 | 5.00 | 4 | 1 |
| 1096 | 102 | 3 | 366 | 1 | 10.19 | 10.00 | 8.00 | 4 | 1 |
| 1097 | 102 | 3 | 366 | 2 | 8.12 | 9.00 | 7.00 | 4 | 1 |
| 1098 | 102 | 3 | 366 | 3 | 15.34 | 12.00 | 10.00 | 4 | 1 |
| 1099 | 103 | 3 | 367 | 1 | 7.38 | 7.50 | 6.50 | 5 | 1 |
| 1100 | 103 | 3 | 367 | 2 | 8.69 | 6.50 | 6.00 | 5 | 1 |
| 1101 | 103 | 3 | 367 | 3 | 11.30 | 8.50 | 6.50 | 5 | 1 |
| 1102 | 104 | 3 | 368 | 1 | 4.71 | 7.00 | 4.00 | 5 | 1 |
| 1103 | 104 | 3 | 368 | 2 | 8.59 | 7.50 | 4.50 | 5 | 1 |
| 1104 | 104 | 3 | 368 | 3 | 0.00 | 0.00 | 0.00 | 0 | 0 |
| 1105 | 105 | 3 | 369 | 1 | 7.00 | 9.00 | 8.00 | 5 | 1 |
| 1106 | 105 | 3 | 369 | 2 | 9.10 | 10.00 | 9.00 | 5 | 1 |
| 1107 | 105 | 3 | 369 | 3 | 10.31 | 11.00 | 10.00 | 5 | 1 |
| 1108 | 106 | 3 | 370 | 1 | 10.35 | 12.00 | 8.50 | 4 | 1 |
| 1109 | 106 | 3 | 370 | 2 | 14.48 | 14.00 | 12.00 | 4 | 1 |
| 1110 | 106 | 3 | 370 | 3 | 9.87 | 7.50 | 5.50 | 4 | 1 |
| 1111 | 107 | 3 | 371 | 1 | 12.54 | 10.00 | 7.00 | 4 | 1 |
| 1112 | 107 | 3 | 371 | 2 | 10.47 | 11.00 | 8.00 | 5 | 1 |
| 1113 | 107 | 3 | 371 | 3 | 12.96 | 9.00 | 7.00 | 4 | 1 |
| 1114 | 108 | 3 | 372 | 1 | 0.00 | 0.00 | 0.00 | 0 | 0 |
| 1115 | 108 | 3 | 372 | 2 | 13.21 | 3.00 | 2.00 | 5 | 1 |
| 1116 | 108 | 3 | 372 | 3 | 10.03 | 9.00 | 7.00 | 4 | 1 |
| 1117 | 109 | 3 | 373 | 1 | 14.39 | 12.00 | 10.00 | 4 | 1 |
| 1118 | 109 | 3 | 373 | 2 | 4.30 | 6.00 | 5.00 | 4 | 1 |
| 1119 | 109 | 3 | 373 | 3 | 16.23 | 12.00 | 9.00 | 5 | 1 |
| 1120 | 110 | 3 | 374 | 1 | 8.69 | 10.00 | 9.00 | 5 | 1 |
| 1121 | 110 | 3 | 374 | 2 | 12.89 | 12.00 | 10.00 | 5 | 1 |
| 1122 | 110 | 3 | 374 | 3 | 9.39 | 11.00 | 9.00 | 5 | 1 |
| 1123 | 111 | 3 | 375 | 1 | 5.25 | 11.00 | 9.00 | 5 | 1 |
| 1124 | 111 | 3 | 375 | 2 | 4.46 | 8.00 | 7.00 | 4 | 1 |
| 1125 | 111 | 3 | 375 | 3 | 5.57 | 7.00 | 6.00 | 5 | 1 |
| 1126 | 112 | 3 | 376 | 1 | 1.75 | 3.00 | 1.50 | 5 | 1 |
| 1127 | 112 | 3 | 376 | 2 | 13.40 | 13.00 | 11.00 | 4 | 1 |
| 1128 | 112 | 3 | 376 | 3 | 12.25 | 11.00 | 8.00 | 5 | 1 |
| 1129 | 113 | 3 | 377 | 1 | 0.00 | 0.00 | 0.00 | 0 | 0 |
| 1130 | 113 | 3 | 377 | 2 | 0.00 | 0.00 | 0.00 | 0 | 0 |
| 1131 | 113 | 3 | 377 | 3 | 13.37 | 12.00 | 10.00 | 5 | 1 |
| 1132 | 114 | 3 | 378 | 1 | 11.14 | 11.00 | 9.00 | 5 | 1 |
| 1133 | 114 | 3 | 378 | 2 | 9.33 | 10.00 | 8.00 | 4 | 1 |
| 1134 | 114 | 3 | 378 | 3 | 9.29 | 8.00 | 7.00 | 4 | 1 |
| 1135 | 115 | 3 | 379 | 1 | 10.09 | 11.00 | 9.00 | 5 | 1 |
| 1136 | 115 | 3 | 379 | 2 | 9.63 | 11.50 | 9.50 | 5 | 1 |
| 1137 | 115 | 3 | 379 | 3 | 12.73 | 12.00 | 9.00 | 5 | 1 |
| 1138 | 116 | 3 | 380 | 1 | 10.35 | 10.00 | 9.00 | 5 | 1 |
| 1139 | 116 | 3 | 380 | 2 | 9.87 | 9.00 | 7.00 | 4 | 1 |
| 1140 | 116 | 3 | 380 | 3 | 5.09 | 7.00 | 5.00 | 6 | 1 |
| 1141 | 117 | 3 | 381 | 1 | 12.73 | 12.00 | 10.00 | 4 | 1 |
| 1142 | 117 | 3 | 381 | 2 | 9.10 | 11.00 | 9.00 | 4 | 1 |
| 1143 | 117 | 3 | 381 | 3 | 11.55 | 10.80 | 7.80 | 5 | 1 |
| 1144 | 118 | 3 | 382 | 1 | 5.51 | 8.00 | 6.00 | 4 | 1 |
| 1145 | 118 | 3 | 382 | 2 | 3.02 | 7.50 | 6.00 | 5 | 1 |
| 1146 | 118 | 3 | 382 | 3 | 5.25 | 6.00 | 5.00 | 5 | 1 |
| 1147 | 119 | 3 | 383 | 1 | 13.69 | 11.00 | 8.00 | 5 | 1 |
| 1148 | 119 | 3 | 383 | 2 | 4.14 | 7.00 | 6.00 | 5 | 1 |
| 1149 | 119 | 3 | 383 | 3 | 2.16 | 6.00 | 5.50 | 5 | 1 |
| 1150 | 120 | 3 | 384 | 1 | 9.64 | 9.00 | 7.00 | 4 | 1 |
| 1151 | 120 | 3 | 384 | 2 | 14.42 | 12.00 | 9.00 | 5 | 1 |
| 1152 | 120 | 3 | 384 | 3 | 10.54 | 11.00 | 9.00 | 4 | 1 |
| 1153 | 121 | 3 | 385 | 1 | 6.05 | 9.50 | 7.50 | 5 | 1 |
| 1154 | 121 | 3 | 385 | 2 | 5.57 | 10.50 | 9.50 | 5 | 1 |
| 1155 | 121 | 3 | 385 | 3 | 13.97 | 13.00 | 11.00 | 5 | 1 |
| 1156 | 122 | 3 | 386 | 1 | 8.75 | 7.50 | 6.50 | 4 | 1 |
| 1157 | 122 | 3 | 386 | 2 | 12.45 | 9.00 | 7.00 | 4 | 1 |
| 1158 | 122 | 3 | 386 | 3 | 4.46 | 7.00 | 6.00 | 5 | 1 |
| 1159 | 123 | 3 | 387 | 1 | 9.07 | 9.00 | 7.00 | 4 | 1 |
| 1160 | 123 | 3 | 387 | 2 | 10.85 | 11.00 | 9.00 | 5 | 1 |
| 1161 | 123 | 3 | 387 | 3 | 12.13 | 10.00 | 7.00 | 5 | 1 |
| 1162 | 124 | 3 | 388 | 1 | 10.19 | 8.00 | 6.00 | 5 | 1 |
| 1163 | 124 | 3 | 388 | 2 | 10.35 | 9.00 | 6.00 | 4 | 1 |
| 1164 | 124 | 3 | 388 | 3 | 11.27 | 10.00 | 8.00 | 4 | 1 |
| 1165 | 125 | 3 | 389 | 1 | 0.00 | 0.00 | 0.00 | 0 | 0 |
| 1166 | 125 | 3 | 389 | 2 | 8.44 | 9.00 | 8.00 | 5 | 1 |
| 1167 | 125 | 3 | 389 | 3 | 6.30 | 7.00 | 6.00 | 5 | 1 |
| 1168 | 126 | 3 | 390 | 1 | 7.42 | 8.00 | 7.00 | 5 | 1 |
| 1169 | 126 | 3 | 390 | 2 | 7.23 | 7.00 | 6.00 | 5 | 1 |
| 1170 | 126 | 3 | 390 | 3 | 3.98 | 6.00 | 1.50 | 4 | 1 |
| 1171 | 127 | 3 | 391 | 1 | 8.05 | 6.00 | 2.00 | 5 | 1 |
| 1172 | 127 | 3 | 391 | 2 | 8.12 | 8.50 | 6.50 | 5 | 1 |
| 1173 | 127 | 3 | 391 | 3 | 8.44 | 9.00 | 7.00 | 4 | 1 |
| 1174 | 128 | 3 | 392 | 1 | 15.09 | 12.00 | 10.00 | 5 | 1 |
| 1175 | 128 | 3 | 392 | 2 | 14.10 | 11.00 | 9.00 | 5 | 1 |
| 1176 | 128 | 3 | 392 | 3 | 15.44 | 10.00 | 7.00 | 5 | 1 |
| 1177 | 129 | 3 | 393 | 1 | 10.03 | 8.00 | 6.00 | 4 | 1 |
| 1178 | 129 | 3 | 393 | 2 | 4.77 | 6.50 | 5.50 | 5 | 1 |
| 1179 | 129 | 3 | 393 | 3 | 11.30 | 8.50 | 7.00 | 5 | 1 |
| 1180 | 130 | 3 | 394 | 1 | 10.66 | 10.00 | 9.00 | 4 | 1 |
| 1181 | 130 | 3 | 394 | 2 | 7.00 | 8.00 | 7.00 | 4 | 1 |
| 1182 | 130 | 3 | 394 | 3 | 12.10 | 10.00 | 8.00 | 5 | 1 |
| 1183 | 131 | 3 | 395 | 1 | 10.60 | 8.50 | 7.50 | 5 | 1 |
| 1184 | 131 | 3 | 395 | 2 | 8.91 | 8.20 | 4.00 | 5 | 1 |
| 1185 | 131 | 3 | 395 | 3 | 10.50 | 7.50 | 5.50 | 5 | 1 |
| 1186 | 132 | 3 | 396 | 1 | 0.00 | 0.00 | 0.00 | 0 | 0 |
| 1187 | 132 | 3 | 396 | 2 | 8.66 | 9.00 | 8.00 | 5 | 1 |
| 1188 | 132 | 3 | 396 | 3 | 0.00 | 0.00 | 0.00 | 0 | 0 |
| 1189 | 1 | 4 | 397 | 1 | 9.55 | 10.30 | 5.00 | 3 | 1 |
| 1190 | 1 | 4 | 397 | 2 | 7.96 | 10.50 | 5.30 | 5 | 1 |
| 1191 | 1 | 4 | 397 | 3 | 12.76 | 11.70 | 6.00 | 5 | 1 |
| 1192 | 2 | 4 | 398 | 1 | 11.71 | 11.00 | 7.00 | 5 | 1 |
| 1193 | 2 | 4 | 398 | 2 | 9.42 | 10.50 | 6.50 | 5 | 1 |
| 1194 | 2 | 4 | 398 | 3 | 5.12 | 9.00 | 7.00 | 5 | 1 |
| 1195 | 3 | 4 | 399 | 1 | 8.98 | 11.00 | 6.50 | 5 | 1 |
| 1196 | 3 | 4 | 399 | 2 | 11.55 | 11.30 | 7.00 | 5 | 1 |
| 1197 | 3 | 4 | 399 | 3 | 0.00 | 0.00 | 0.00 | 0 | 0 |
| 1198 | 4 | 4 | 400 | 1 | 11.87 | 10.50 | 5.00 | 5 | 1 |
| 1199 | 4 | 4 | 400 | 2 | 9.36 | 10.80 | 4.50 | 4 | 1 |
| 1200 | 4 | 4 | 400 | 3 | 9.68 | 11.00 | 7.00 | 5 | 1 |
| 1201 | 5 | 4 | 401 | 1 | 11.94 | 7.50 | 5.00 | 5 | 1 |
| 1202 | 5 | 4 | 401 | 2 | 0.00 | 0.00 | 0.00 | 0 | 0 |
| 1203 | 5 | 4 | 401 | 3 | 3.34 | 5.00 | 2.80 | 4 | 1 |
| 1204 | 6 | 4 | 402 | 1 | 14.04 | 13.00 | 10.00 | 5 | 1 |
| 1205 | 6 | 4 | 402 | 2 | 13.21 | 13.50 | 9.50 | 5 | 1 |
| 1206 | 6 | 4 | 402 | 3 | 13.08 | 13.50 | 9.00 | 4 | 1 |
| 1207 | 7 | 4 | 403 | 1 | 9.23 | 10.00 | 6.00 | 5 | 1 |
| 1208 | 7 | 4 | 403 | 2 | 4.90 | 7.00 | 4.80 | 5 | 1 |
| 1209 | 7 | 4 | 403 | 3 | 7.42 | 10.00 | 5.50 | 5 | 1 |
| 1210 | 8 | 4 | 404 | 1 | 7.83 | 8.30 | 5.30 | 5 | 1 |
| 1211 | 8 | 4 | 404 | 2 | 10.89 | 9.00 | 5.00 | 5 | 1 |
| 1212 | 8 | 4 | 404 | 3 | 8.79 | 10.20 | 6.00 | 4 | 1 |
| 1213 | 9 | 4 | 405 | 1 | 11.40 | 11.50 | 7.50 | 5 | 1 |
| 1214 | 9 | 4 | 405 | 2 | 10.95 | 11.20 | 8.00 | 5 | 1 |
| 1215 | 9 | 4 | 405 | 3 | 8.40 | 11.00 | 7.80 | 5 | 1 |
| 1216 | 10 | 4 | 406 | 1 | 8.82 | 9.60 | 6.00 | 4 | 1 |
| 1217 | 10 | 4 | 406 | 2 | 4.90 | 7.60 | 6.00 | 5 | 1 |
| 1218 | 10 | 4 | 406 | 3 | 10.44 | 9.80 | 6.50 | 5 | 1 |
| 1219 | 11 | 4 | 407 | 1 | 6.75 | 8.50 | 4.00 | 4 | 1 |
| 1220 | 11 | 4 | 407 | 2 | 4.42 | 5.00 | 2.50 | 4 | 1 |
| 1221 | 11 | 4 | 407 | 3 | 0.00 | 0.00 | 0.00 | 0 | 0 |
| 1222 | 12 | 4 | 408 | 1 | 10.95 | 11.00 | 5.50 | 5 | 1 |
| 1223 | 12 | 4 | 408 | 2 | 7.51 | 10.50 | 6.00 | 5 | 1 |
| 1224 | 12 | 4 | 408 | 3 | 8.09 | 10.50 | 6.50 | 5 | 1 |
| 1225 | 13 | 4 | 409 | 1 | 0.00 | 0.00 | 0.00 | 0 | 0 |
| 1226 | 13 | 4 | 409 | 2 | 0.00 | 0.00 | 0.00 | 0 | 0 |
| 1227 | 13 | 4 | 409 | 3 | 5.92 | 7.50 | 4.50 | 4 | 1 |
| 1228 | 14 | 4 | 410 | 1 | 1.11 | 2.30 | 1.00 | 4 | 1 |
| 1229 | 14 | 4 | 410 | 2 | 7.96 | 8.50 | 5.50 | 5 | 1 |
| 1230 | 14 | 4 | 410 | 3 | 12.99 | 11.80 | 7.50 | 5 | 1 |
| 1231 | 15 | 4 | 411 | 1 | 14.64 | 12.50 | 9.00 | 5 | 1 |
| 1232 | 15 | 4 | 411 | 2 | 16.11 | 12.50 | 7.50 | 3 | 1 |
| 1233 | 15 | 4 | 411 | 3 | 9.93 | 9.50 | 5.00 | 5 | 1 |
| 1234 | 16 | 4 | 412 | 1 | 13.21 | 11.00 | 7.00 | 4 | 1 |
| 1235 | 16 | 4 | 412 | 2 | 7.99 | 8.00 | 6.50 | 5 | 1 |
| 1236 | 16 | 4 | 412 | 3 | 8.66 | 7.60 | 5.00 | 5 | 1 |
| 1237 | 17 | 4 | 413 | 1 | 0.00 | 0.00 | 0.00 | 0 | 0 |
| 1238 | 17 | 4 | 413 | 2 | 6.40 | 12.50 | 4.50 | 4 | 1 |
| 1239 | 17 | 4 | 413 | 3 | 11.17 | 11.00 | 5.00 | 5 | 1 |
| 1240 | 18 | 4 | 414 | 1 | 9.99 | 10.50 | 8.00 | 5 | 1 |
| 1241 | 18 | 4 | 414 | 2 | 10.54 | 11.50 | 8.00 | 5 | 1 |
| 1242 | 18 | 4 | 414 | 3 | 12.03 | 11.50 | 7.00 | 5 | 1 |
| 1243 | 19 | 4 | 415 | 1 | 10.73 | 12.00 | 8.00 | 5 | 1 |
| 1244 | 19 | 4 | 415 | 2 | 14.71 | 13.50 | 9.50 | 4 | 1 |
| 1245 | 19 | 4 | 415 | 3 | 8.94 | 11.50 | 7.00 | 5 | 1 |
| 1246 | 20 | 4 | 416 | 1 | 12.76 | 12.30 | 6.50 | 5 | 1 |
| 1247 | 20 | 4 | 416 | 2 | 8.91 | 11.50 | 6.50 | 4 | 1 |
| 1248 | 20 | 4 | 416 | 3 | 12.80 | 11.50 | 6.00 | 5 | 1 |
| 1249 | 21 | 4 | 417 | 1 | 8.59 | 8.50 | 6.50 | 5 | 1 |
| 1250 | 21 | 4 | 417 | 2 | 11.90 | 9.80 | 6.00 | 4 | 1 |
| 1251 | 21 | 4 | 417 | 3 | 7.45 | 7.00 | 4.00 | 5 | 1 |
| 1252 | 22 | 4 | 418 | 1 | 0.00 | 0.00 | 0.00 | 0 | 0 |
| 1253 | 22 | 4 | 418 | 2 | 10.85 | 10.50 | 7.20 | 5 | 1 |
| 1254 | 22 | 4 | 418 | 3 | 11.97 | 10.80 | 6.00 | 5 | 1 |
| 1255 | 23 | 4 | 419 | 1 | 0.00 | 0.00 | 0.00 | 0 | 0 |
| 1256 | 23 | 4 | 419 | 2 | 10.44 | 10.00 | 6.00 | 5 | 1 |
| 1257 | 23 | 4 | 419 | 3 | 10.54 | 9.50 | 6.00 | 5 | 1 |
| 1258 | 24 | 4 | 420 | 1 | 10.54 | 11.30 | 6.50 | 5 | 1 |
| 1259 | 24 | 4 | 420 | 2 | 8.34 | 10.50 | 5.50 | 4 | 1 |
| 1260 | 24 | 4 | 420 | 3 | 12.32 | 12.00 | 6.00 | 5 | 1 |
| 1261 | 25 | 4 | 421 | 1 | 11.65 | 9.00 | 6.50 | 4 | 1 |
| 1262 | 25 | 4 | 421 | 2 | 8.98 | 8.80 | 6.50 | 4 | 1 |
| 1263 | 25 | 4 | 421 | 3 | 10.57 | 9.00 | 3.00 | 4 | 1 |
| 1264 | 26 | 4 | 422 | 1 | 4.52 | 4.50 | 2.30 | 5 | 1 |
| 1265 | 26 | 4 | 422 | 2 | 5.17 | 9.00 | 3.00 | 3 | 1 |
| 1266 | 26 | 4 | 422 | 3 | 0.00 | 0.00 | 0.00 | 0 | 0 |
| 1267 | 27 | 4 | 423 | 1 | 5.98 | 9.20 | 7.30 | 4 | 1 |
| 1268 | 27 | 4 | 423 | 2 | 10.89 | 10.50 | 7.50 | 5 | 1 |
| 1269 | 27 | 4 | 423 | 3 | 7.99 | 10.00 | 6.00 | 5 | 1 |
| 1270 | 28 | 4 | 424 | 1 | 9.71 | 9.00 | 5.00 | 4 | 1 |
| 1271 | 28 | 4 | 424 | 2 | 1.27 | 2.00 | 0.40 | 4 | 1 |
| 1272 | 28 | 4 | 424 | 3 | 9.23 | 9.00 | 5.50 | 4 | 1 |
| 1273 | 29 | 4 | 425 | 1 | 12.57 | 10.50 | 5.50 | 3 | 1 |
| 1274 | 29 | 4 | 425 | 2 | 14.71 | 11.00 | 6.00 | 4 | 1 |
| 1275 | 29 | 4 | 425 | 3 | 5.00 | 5.50 | 1.80 | 5 | 1 |
| 1276 | 30 | 4 | 426 | 1 | 14.26 | 12.50 | 7.00 | 5 | 1 |
| 1277 | 30 | 4 | 426 | 2 | 8.15 | 9.50 | 1.50 | 4 | 1 |
| 1278 | 30 | 4 | 426 | 3 | 15.60 | 2.30 | 1.80 | 4 | 1 |
| 1279 | 31 | 4 | 427 | 1 | 12.29 | 10.20 | 7.50 | 5 | 1 |
| 1280 | 31 | 4 | 427 | 2 | 11.30 | 10.50 | 7.00 | 5 | 1 |
| 1281 | 31 | 4 | 427 | 3 | 10.31 | 10.00 | 7.50 | 5 | 1 |
| 1282 | 32 | 4 | 428 | 1 | 10.44 | 10.30 | 7.00 | 5 | 1 |
| 1283 | 32 | 4 | 428 | 2 | 8.91 | 10.50 | 7.00 | 5 | 1 |
| 1284 | 32 | 4 | 428 | 3 | 11.49 | 10.50 | 7.00 | 5 | 1 |
| 1285 | 33 | 4 | 429 | 1 | 13.43 | 10.80 | 6.00 | 4 | 1 |
| 1286 | 33 | 4 | 429 | 2 | 6.11 | 7.50 | 4.50 | 4 | 1 |
| 1287 | 33 | 4 | 429 | 3 | 5.28 | 7.50 | 5.00 | 4 | 1 |
| 1288 | 34 | 4 | 430 | 1 | 9.77 | 10.00 | 7.00 | 4 | 1 |
| 1289 | 34 | 4 | 430 | 2 | 10.25 | 9.50 | 7.00 | 4 | 1 |
| 1290 | 34 | 4 | 430 | 3 | 9.58 | 9.50 | 7.50 | 5 | 1 |
| 1291 | 35 | 4 | 431 | 1 | 9.61 | 11.00 | 7.50 | 4 | 1 |
| 1292 | 35 | 4 | 431 | 2 | 11.40 | 11.30 | 8.00 | 5 | 1 |
| 1293 | 35 | 4 | 431 | 3 | 8.21 | 9.50 | 6.00 | 5 | 1 |
| 1294 | 36 | 4 | 432 | 1 | 2.71 | 7.30 | 1.80 | 5 | 1 |
| 1295 | 36 | 4 | 432 | 2 | 11.30 | 10.50 | 7.00 | 5 | 1 |
| 1296 | 36 | 4 | 432 | 3 | 0.95 | 2.80 | 1.80 | 3 | 1 |
| 1297 | 37 | 4 | 433 | 1 | 1.75 | 3.60 | 1.50 | 3 | 1 |
| 1298 | 37 | 4 | 433 | 2 | 5.73 | 5.50 | 1.80 | 3 | 1 |
| 1299 | 37 | 4 | 433 | 3 | 4.33 | 7.50 | 3.60 | 5 | 1 |
| 1300 | 38 | 4 | 434 | 1 | 15.02 | 12.50 | 7.50 | 5 | 1 |
| 1301 | 38 | 4 | 434 | 2 | 15.66 | 13.00 | 8.00 | 5 | 1 |
| 1302 | 38 | 4 | 434 | 3 | 14.90 | 11.50 | 8.50 | 5 | 1 |
| 1303 | 39 | 4 | 435 | 1 | 13.69 | 9.30 | 5.00 | 5 | 1 |
| 1304 | 39 | 4 | 435 | 2 | 0.00 | 0.00 | 0.00 | 0 | 0 |
| 1305 | 39 | 4 | 435 | 3 | 7.19 | 7.50 | 3.50 | 4 | 1 |
| 1306 | 40 | 4 | 436 | 1 | 7.00 | 9.00 | 6.00 | 5 | 1 |
| 1307 | 40 | 4 | 436 | 2 | 12.45 | 10.30 | 6.50 | 5 | 1 |
| 1308 | 40 | 4 | 436 | 3 | 12.45 | 10.00 | 7.50 | 5 | 1 |
| 1309 | 41 | 4 | 437 | 1 | 12.57 | 10.50 | 7.00 | 5 | 1 |
| 1310 | 41 | 4 | 437 | 2 | 9.01 | 9.80 | 7.00 | 5 | 1 |
| 1311 | 41 | 4 | 437 | 3 | 15.41 | 10.50 | 7.00 | 5 | 1 |
| 1312 | 42 | 4 | 438 | 1 | 13.75 | 9.80 | 7.00 | 5 | 1 |
| 1313 | 42 | 4 | 438 | 2 | 5.28 | 5.20 | 3.20 | 5 | 1 |
| 1314 | 42 | 4 | 438 | 3 | 9.14 | 8.80 | 5.20 | 5 | 1 |
| 1315 | 43 | 4 | 439 | 1 | 11.43 | 10.00 | 7.00 | 5 | 1 |
| 1316 | 43 | 4 | 439 | 2 | 14.67 | 13.00 | 8.50 | 5 | 1 |
| 1317 | 43 | 4 | 439 | 3 | 16.07 | 12.50 | 8.50 | 5 | 1 |
| 1318 | 44 | 4 | 440 | 1 | 12.13 | 11.50 | 6.00 | 4 | 1 |
| 1319 | 44 | 4 | 440 | 2 | 12.67 | 12.50 | 8.00 | 4 | 1 |
| 1320 | 44 | 4 | 440 | 3 | 6.88 | 10.00 | 6.00 | 4 | 1 |
| 1321 | 45 | 4 | 441 | 1 | 2.61 | 3.60 | 1.50 | 5 | 1 |
| 1322 | 45 | 4 | 441 | 2 | 9.01 | 11.50 | 7.50 | 5 | 1 |
| 1323 | 45 | 4 | 441 | 3 | 0.00 | 0.00 | 0.00 | 0 | 0 |
| 1324 | 46 | 4 | 442 | 1 | 0.00 | 0.00 | 0.00 | 0 | 0 |
| 1325 | 46 | 4 | 442 | 2 | 12.76 | 11.00 | 7.50 | 5 | 1 |
| 1326 | 46 | 4 | 442 | 3 | 12.22 | 10.20 | 7.00 | 5 | 1 |
| 1327 | 47 | 4 | 443 | 1 | 10.70 | 10.00 | 6.50 | 5 | 1 |
| 1328 | 47 | 4 | 443 | 2 | 10.57 | 9.00 | 6.00 | 3 | 1 |
| 1329 | 47 | 4 | 443 | 3 | 11.43 | 9.50 | 7.00 | 5 | 1 |
| 1330 | 48 | 4 | 444 | 1 | 13.24 | 11.20 | 7.50 | 5 | 1 |
| 1331 | 48 | 4 | 444 | 2 | 5.16 | 7.20 | 4.80 | 5 | 1 |
| 1332 | 48 | 4 | 444 | 3 | 8.02 | 9.00 | 4.00 | 5 | 1 |
| 1333 | 49 | 4 | 445 | 1 | 14.55 | 12.80 | 8.50 | 4 | 1 |
| 1334 | 49 | 4 | 445 | 2 | 12.25 | 11.00 | 8.00 | 3 | 1 |
| 1335 | 49 | 4 | 445 | 3 | 13.59 | 11.50 | 8.00 | 4 | 1 |
| 1336 | 50 | 4 | 446 | 1 | 11.87 | 11.50 | 9.00 | 4 | 1 |
| 1337 | 50 | 4 | 446 | 2 | 7.99 | 9.80 | 6.80 | 5 | 1 |
| 1338 | 50 | 4 | 446 | 3 | 9.23 | 10.80 | 6.80 | 4 | 1 |
| 1339 | 51 | 4 | 447 | 1 | 12.16 | 9.80 | 6.00 | 5 | 1 |
| 1340 | 51 | 4 | 447 | 2 | 11.33 | 10.00 | 5.80 | 5 | 1 |
| 1341 | 51 | 4 | 447 | 3 | 10.09 | 9.70 | 6.00 | 5 | 1 |
| 1342 | 52 | 4 | 448 | 1 | 5.89 | 9.00 | 5.00 | 5 | 1 |
| 1343 | 52 | 4 | 448 | 2 | 0.00 | 0.00 | 0.00 | 0 | 0 |
| 1344 | 52 | 4 | 448 | 3 | 8.79 | 11.30 | 6.00 | 4 | 1 |
| 1345 | 53 | 4 | 449 | 1 | 6.21 | 8.70 | 5.00 | 5 | 1 |
| 1346 | 53 | 4 | 449 | 2 | 10.28 | 10.60 | 5.00 | 5 | 1 |
| 1347 | 53 | 4 | 449 | 3 | 3.53 | 7.50 | 4.00 | 4 | 1 |
| 1348 | 54 | 4 | 450 | 1 | 2.07 | 4.00 | 1.20 | 4 | 1 |
| 1349 | 54 | 4 | 450 | 2 | 9.29 | 9.50 | 6.00 | 5 | 1 |
| 1350 | 54 | 4 | 450 | 3 | 8.18 | 9.20 | 6.00 | 5 | 1 |
| 1351 | 55 | 4 | 451 | 1 | 13.81 | 9.00 | 5.00 | 3 | 1 |
| 1352 | 55 | 4 | 451 | 2 | 12.03 | 9.50 | 6.50 | 5 | 1 |
| 1353 | 55 | 4 | 451 | 3 | 14.67 | 10.00 | 5.00 | 4 | 1 |
| 1354 | 56 | 4 | 452 | 1 | 2.48 | 3.70 | 2.00 | 3 | 1 |
| 1355 | 56 | 4 | 452 | 2 | 6.65 | 8.70 | 3.50 | 5 | 1 |
| 1356 | 56 | 4 | 452 | 3 | 7.35 | 9.50 | 5.50 | 4 | 1 |
| 1357 | 57 | 4 | 453 | 1 | 8.79 | 10.00 | 7.00 | 5 | 1 |
| 1358 | 57 | 4 | 453 | 2 | 13.72 | 11.50 | 8.00 | 5 | 1 |
| 1359 | 57 | 4 | 453 | 3 | 13.85 | 11.00 | 6.00 | 5 | 1 |
| 1360 | 58 | 4 | 454 | 1 | 11.81 | 11.00 | 4.50 | 3 | 1 |
| 1361 | 58 | 4 | 454 | 2 | 14.48 | 11.20 | 7.50 | 4 | 1 |
| 1362 | 58 | 4 | 454 | 3 | 13.94 | 11.50 | 8.00 | 3 | 1 |
| 1363 | 59 | 4 | 455 | 1 | 10.73 | 11.00 | 7.50 | 5 | 1 |
| 1364 | 59 | 4 | 455 | 2 | 6.24 | 9.00 | 6.50 | 5 | 1 |
| 1365 | 59 | 4 | 455 | 3 | 7.99 | 9.50 | 6.00 | 5 | 1 |
| 1366 | 60 | 4 | 456 | 1 | 11.68 | 11.80 | 7.00 | 5 | 1 |
| 1367 | 60 | 4 | 456 | 2 | 13.78 | 12.00 | 7.00 | 5 | 1 |
| 1368 | 60 | 4 | 456 | 3 | 10.44 | 10.70 | 7.00 | 5 | 1 |
| 1369 | 61 | 4 | 457 | 1 | 8.50 | 9.20 | 5.50 | 5 | 1 |
| 1370 | 61 | 4 | 457 | 2 | 8.91 | 9.60 | 6.00 | 5 | 1 |
| 1371 | 61 | 4 | 457 | 3 | 4.90 | 7.50 | 5.50 | 4 | 1 |
| 1372 | 62 | 4 | 458 | 1 | 10.25 | 10.70 | 6.50 | 5 | 1 |
| 1373 | 62 | 4 | 458 | 2 | 8.53 | 8.70 | 6.00 | 4 | 1 |
| 1374 | 62 | 4 | 458 | 3 | 13.78 | 11.00 | 7.50 | 5 | 1 |
| 1375 | 63 | 4 | 459 | 1 | 7.03 | 9.50 | 7.00 | 3 | 1 |
| 1376 | 63 | 4 | 459 | 2 | 13.43 | 10.30 | 6.00 | 4 | 1 |
| 1377 | 63 | 4 | 459 | 3 | 7.35 | 9.50 | 7.00 | 4 | 1 |
| 1378 | 64 | 4 | 460 | 1 | 13.53 | 10.80 | 7.50 | 5 | 1 |
| 1379 | 64 | 4 | 460 | 2 | 11.81 | 9.80 | 7.00 | 5 | 1 |
| 1380 | 64 | 4 | 460 | 3 | 15.12 | 11.00 | 7.00 | 5 | 1 |
| 1381 | 65 | 4 | 461 | 1 | 5.38 | 9.00 | 7.00 | 5 | 1 |
| 1382 | 65 | 4 | 461 | 2 | 11.20 | 11.30 | 7.00 | 5 | 1 |
| 1383 | 65 | 4 | 461 | 3 | 0.00 | 0.00 | 0.00 | 0 | 0 |
| 1384 | 66 | 4 | 462 | 1 | 1.69 | 3.40 | 1.60 | 5 | 1 |
| 1385 | 66 | 4 | 462 | 2 | 1.02 | 2.00 | 1.50 | 5 | 1 |
| 1386 | 66 | 4 | 462 | 3 | 7.51 | 8.00 | 5.50 | 5 | 1 |
| 1387 | 67 | 4 | 463 | 1 | 6.75 | 8.50 | 6.00 | 5 | 1 |
| 1388 | 67 | 4 | 463 | 2 | 6.76 | 12.00 | 9.20 | 5 | 1 |
| 1389 | 67 | 4 | 463 | 3 | 9.36 | 9.50 | 6.00 | 5 | 1 |
| 1390 | 68 | 4 | 464 | 1 | 11.01 | 9.90 | 5.50 | 5 | 1 |
| 1391 | 68 | 4 | 464 | 2 | 7.11 | 10.20 | 3.30 | 5 | 1 |
| 1392 | 68 | 4 | 464 | 3 | 11.52 | 9.20 | 5.00 | 5 | 1 |
| 1393 | 69 | 4 | 465 | 1 | 5.47 | 7.80 | 3.80 | 5 | 1 |
| 1394 | 69 | 4 | 465 | 2 | 5.35 | 7.00 | 4.20 | 5 | 1 |
| 1395 | 69 | 4 | 465 | 3 | 7.19 | 8.20 | 5.00 | 4 | 1 |
| 1396 | 70 | 4 | 466 | 1 | 7.67 | 8.50 | 4.00 | 5 | 1 |
| 1397 | 70 | 4 | 466 | 2 | 12.57 | 9.70 | 3.60 | 4 | 1 |
| 1398 | 70 | 4 | 466 | 3 | 9.42 | 9.20 | 5.00 | 5 | 1 |
| 1399 | 71 | 4 | 467 | 1 | 11.01 | 10.80 | 5.40 | 5 | 1 |
| 1400 | 71 | 4 | 467 | 2 | 8.02 | 10.00 | 5.50 | 5 | 1 |
| 1401 | 71 | 4 | 467 | 3 | 0.00 | 0.00 | 0.00 | 0 | 0 |
| 1402 | 72 | 4 | 468 | 1 | 6.14 | 7.00 | 2.30 | 1 | 1 |
| 1403 | 72 | 4 | 468 | 2 | 5.25 | 6.70 | 3.20 | 4 | 1 |
| 1404 | 72 | 4 | 468 | 3 | 6.81 | 7.50 | 4.50 | 4 | 1 |
| 1405 | 73 | 4 | 469 | 1 | 6.08 | 8.50 | 5.00 | 4 | 1 |
| 1406 | 73 | 4 | 469 | 2 | 7.32 | 8.70 | 3.20 | 4 | 1 |
| 1407 | 73 | 4 | 469 | 3 | 0.00 | 0.00 | 0.00 | 0 | 0 |
| 1408 | 74 | 4 | 470 | 1 | 0.00 | 0.00 | 0.00 | 0 | 0 |
| 1409 | 74 | 4 | 470 | 2 | 1.66 | 2.50 | 1.50 | 4 | 1 |
| 1410 | 74 | 4 | 470 | 3 | 0.00 | 0.00 | 0.00 | 0 | 0 |
| 1411 | 75 | 4 | 471 | 1 | 12.86 | 9.50 | 6.00 | 5 | 1 |
| 1412 | 75 | 4 | 471 | 2 | 7.19 | 9.00 | 6.00 | 5 | 1 |
| 1413 | 75 | 4 | 471 | 3 | 12.21 | 11.00 | 7.00 | 5 | 1 |
| 1414 | 76 | 4 | 472 | 1 | 9.90 | 12.50 | 9.00 | 5 | 1 |
| 1415 | 76 | 4 | 472 | 2 | 11.20 | 12.80 | 8.00 | 5 | 1 |
| 1416 | 76 | 4 | 472 | 3 | 12.38 | 13.00 | 8.50 | 5 | 1 |
| 1417 | 77 | 4 | 473 | 1 | 9.55 | 7.80 | 5.50 | 5 | 1 |
| 1418 | 77 | 4 | 473 | 2 | 9.39 | 7.50 | 5.00 | 5 | 1 |
| 1419 | 77 | 4 | 473 | 3 | 12.32 | 10.00 | 7.50 | 5 | 1 |
| 1420 | 78 | 4 | 474 | 1 | 5.51 | 6.00 | 4.70 | 5 | 1 |
| 1421 | 78 | 4 | 474 | 2 | 3.88 | 5.80 | 4.00 | 5 | 1 |
| 1422 | 78 | 4 | 474 | 3 | 12.57 | 9.00 | 4.00 | 3 | 1 |
| 1423 | 79 | 4 | 475 | 1 | 8.02 | 9.00 | 5.00 | 5 | 1 |
| 1424 | 79 | 4 | 475 | 2 | 13.21 | 11.00 | 5.90 | 4 | 1 |
| 1425 | 79 | 4 | 475 | 3 | 8.82 | 11.00 | 6.00 | 5 | 1 |
| 1426 | 80 | 4 | 476 | 1 | 12.32 | 11.50 | 6.00 | 4 | 1 |
| 1427 | 80 | 4 | 476 | 2 | 7.67 | 10.50 | 6.00 | 5 | 1 |
| 1428 | 80 | 4 | 476 | 3 | 7.07 | 10.80 | 6.50 | 5 | 1 |
| 1429 | 81 | 4 | 477 | 1 | 11.17 | 11.00 | 8.50 | 5 | 1 |
| 1430 | 81 | 4 | 477 | 2 | 10.47 | 11.30 | 7.80 | 4 | 1 |
| 1431 | 81 | 4 | 477 | 3 | 9.26 | 10.20 | 7.00 | 5 | 1 |
| 1432 | 82 | 4 | 478 | 1 | 0.00 | 0.00 | 0.00 | 0 | 0 |
| 1433 | 82 | 4 | 478 | 2 | 6.94 | 7.00 | 3.50 | 4 | 1 |
| 1434 | 82 | 4 | 478 | 3 | 2.05 | 4.00 | 1.80 | 3 | 1 |
| 1435 | 83 | 4 | 479 | 1 | 10.70 | 9.50 | 6.00 | 5 | 1 |
| 1436 | 83 | 4 | 479 | 2 | 7.80 | 7.50 | 4.00 | 5 | 1 |
| 1437 | 83 | 4 | 479 | 3 | 9.93 | 8.50 | 4.00 | 5 | 1 |
| 1438 | 84 | 4 | 480 | 1 | 1.53 | 2.50 | 1.30 | 5 | 1 |
| 1439 | 84 | 4 | 480 | 2 | 6.49 | 7.00 | 5.30 | 5 | 1 |
| 1440 | 84 | 4 | 480 | 3 | 12.76 | 10.50 | 7.00 | 4 | 1 |
| 1441 | 85 | 4 | 481 | 1 | 5.03 | 8.90 | 6.00 | 5 | 1 |
| 1442 | 85 | 4 | 481 | 2 | 10.12 | 11.00 | 7.50 | 5 | 1 |
| 1443 | 85 | 4 | 481 | 3 | 8.66 | 9.50 | 5.00 | 5 | 1 |
| 1444 | 86 | 4 | 482 | 1 | 11.49 | 11.00 | 8.00 | 5 | 1 |
| 1445 | 86 | 4 | 482 | 2 | 8.21 | 10.50 | 8.00 | 5 | 1 |
| 1446 | 86 | 4 | 482 | 3 | 13.88 | 12.00 | 8.50 | 5 | 1 |
| 1447 | 87 | 4 | 483 | 1 | 10.57 | 10.00 | 7.00 | 5 | 1 |
| 1448 | 87 | 4 | 483 | 2 | 13.66 | 11.00 | 7.50 | 5 | 1 |
| 1449 | 87 | 4 | 483 | 3 | 1.75 | 2.40 | 1.30 | 5 | 1 |
| 1450 | 88 | 4 | 484 | 1 | 10.12 | 9.30 | 5.00 | 5 | 1 |
| 1451 | 88 | 4 | 484 | 2 | 0.00 | 0.00 | 0.00 | 0 | 0 |
| 1452 | 88 | 4 | 484 | 3 | 7.99 | 9.50 | 4.50 | 5 | 1 |
| 1453 | 89 | 4 | 485 | 1 | 8.59 | 10.30 | 7.00 | 5 | 1 |
| 1454 | 89 | 4 | 485 | 2 | 13.11 | 10.40 | 5.00 | 5 | 1 |
| 1455 | 89 | 4 | 485 | 3 | 13.46 | 12.30 | 6.00 | 5 | 1 |
| 1456 | 90 | 4 | 486 | 1 | 14.18 | 11.50 | 4.00 | 5 | 1 |
| 1457 | 90 | 4 | 486 | 2 | 6.72 | 8.80 | 3.50 | 4 | 1 |
| 1458 | 90 | 4 | 486 | 3 | 11.20 | 9.80 | 6.00 | 5 | 1 |
| 1459 | 91 | 4 | 487 | 1 | 12.45 | 9.50 | 7.00 | 5 | 1 |
| 1460 | 91 | 4 | 487 | 2 | 11.78 | 9.00 | 6.50 | 5 | 1 |
| 1461 | 91 | 4 | 487 | 3 | 10.98 | 8.50 | 6.00 | 5 | 1 |
| 1462 | 92 | 4 | 488 | 1 | 6.37 | 6.50 | 2.30 | 5 | 1 |
| 1463 | 92 | 4 | 488 | 2 | 0.00 | 0.00 | 0.00 | 0 | 0 |
| 1464 | 92 | 4 | 488 | 3 | 2.47 | 7.00 | 2.30 | 5 | 1 |
| 1465 | 93 | 4 | 489 | 1 | 15.76 | 12.50 | 8.00 | 5 | 1 |
| 1466 | 93 | 4 | 489 | 2 | 12.41 | 10.00 | 7.00 | 5 | 1 |
| 1467 | 93 | 4 | 489 | 3 | 12.19 | 9.80 | 7.00 | 5 | 1 |
| 1468 | 94 | 4 | 490 | 1 | 3.06 | 4.00 | 1.50 | 5 | 1 |
| 1469 | 94 | 4 | 490 | 2 | 12.13 | 13.00 | 8.00 | 5 | 1 |
| 1470 | 94 | 4 | 490 | 3 | 9.96 | 12.50 | 8.50 | 5 | 1 |
| 1471 | 95 | 4 | 491 | 1 | 12.22 | 13.80 | 8.50 | 5 | 1 |
| 1472 | 95 | 4 | 491 | 2 | 13.02 | 13.40 | 9.00 | 5 | 1 |
| 1473 | 95 | 4 | 491 | 3 | 16.65 | 13.30 | 8.50 | 5 | 1 |
| 1474 | 96 | 4 | 492 | 1 | 7.35 | 8.70 | 4.00 | 4 | 1 |
| 1475 | 96 | 4 | 492 | 2 | 7.23 | 8.50 | 4.50 | 5 | 1 |
| 1476 | 96 | 4 | 492 | 3 | 6.81 | 8.40 | 4.20 | 4 | 1 |
| 1477 | 97 | 4 | 493 | 1 | 8.91 | 10.80 | 5.50 | 5 | 1 |
| 1478 | 97 | 4 | 493 | 2 | 0.00 | 0.00 | 0.00 | 0 | 0 |
| 1479 | 97 | 4 | 493 | 3 | 10.60 | 11.30 | 6.50 | 5 | 1 |
| 1480 | 98 | 4 | 494 | 1 | 0.00 | 0.00 | 0.00 | 0 | 0 |
| 1481 | 98 | 4 | 494 | 2 | 6.53 | 7.30 | 4.00 | 4 | 1 |
| 1482 | 98 | 4 | 494 | 3 | 11.78 | 11.50 | 7.50 | 5 | 1 |
| 1483 | 99 | 4 | 495 | 1 | 10.38 | 11.20 | 6.00 | 5 | 1 |
| 1484 | 99 | 4 | 495 | 2 | 10.54 | 11.00 | 5.50 | 4 | 1 |
| 1485 | 99 | 4 | 495 | 3 | 9.45 | 11.00 | 5.00 | 5 | 1 |
| 1486 | 100 | 4 | 496 | 1 | 8.18 | 9.50 | 5.50 | 5 | 1 |
| 1487 | 100 | 4 | 496 | 2 | 5.35 | 8.30 | 5.20 | 4 | 1 |
| 1488 | 100 | 4 | 496 | 3 | 6.94 | 8.80 | 6.00 | 4 | 1 |
| 1489 | 101 | 4 | 497 | 1 | 12.86 | 11.50 | 6.50 | 5 | 1 |
| 1490 | 101 | 4 | 497 | 2 | 9.58 | 10.50 | 8.00 | 4 | 1 |
| 1491 | 101 | 4 | 497 | 3 | 9.42 | 10.00 | 5.00 | 5 | 1 |
| 1492 | 102 | 4 | 498 | 1 | 0.00 | 0.00 | 0.00 | 0 | 0 |
| 1493 | 102 | 4 | 498 | 2 | 7.67 | 10.30 | 5.00 | 4 | 1 |
| 1494 | 102 | 4 | 498 | 3 | 8.05 | 9.00 | 3.80 | 5 | 1 |
| 1495 | 103 | 4 | 499 | 1 | 9.68 | 9.50 | 6.80 | 5 | 1 |
| 1496 | 103 | 4 | 499 | 2 | 9.23 | 9.50 | 6.50 | 5 | 1 |
| 1497 | 103 | 4 | 499 | 3 | 11.40 | 9.70 | 7.00 | 5 | 1 |
| 1498 | 104 | 4 | 500 | 1 | 16.68 | 10.20 | 1.80 | 2 | 1 |
| 1499 | 104 | 4 | 500 | 2 | 10.98 | 11.30 | 7.00 | 4 | 1 |
| 1500 | 104 | 4 | 500 | 3 | 4.19 | 9.00 | 2.30 | 2 | 1 |
| 1501 | 105 | 4 | 501 | 1 | 10.25 | 9.50 | 7.00 | 5 | 1 |
| 1502 | 105 | 4 | 501 | 2 | 0.00 | 0.00 | 0.00 | 0 | 0 |
| 1503 | 105 | 4 | 501 | 3 | 8.94 | 8.00 | 4.50 | 5 | 1 |
| 1504 | 106 | 4 | 502 | 1 | 10.06 | 11.30 | 8.00 | 5 | 1 |
| 1505 | 106 | 4 | 502 | 2 | 10.06 | 11.00 | 7.50 | 5 | 1 |
| 1506 | 106 | 4 | 502 | 3 | 12.38 | 11.20 | 7.50 | 4 | 1 |
| 1507 | 107 | 4 | 503 | 1 | 6.18 | 7.50 | 5.90 | 5 | 1 |
| 1508 | 107 | 4 | 503 | 2 | 7.89 | 9.50 | 7.00 | 4 | 1 |
| 1509 | 107 | 4 | 503 | 3 | 11.05 | 9.80 | 7.00 | 5 | 1 |
| 1510 | 108 | 4 | 504 | 1 | 8.88 | 7.70 | 4.50 | 5 | 1 |
| 1511 | 108 | 4 | 504 | 2 | 8.37 | 8.50 | 6.00 | 5 | 1 |
| 1512 | 108 | 4 | 504 | 3 | 10.98 | 8.50 | 6.00 | 4 | 1 |
| 1513 | 109 | 4 | 505 | 1 | 10.41 | 9.50 | 6.00 | 4 | 1 |
| 1514 | 109 | 4 | 505 | 2 | 8.12 | 9.20 | 5.50 | 4 | 1 |
| 1515 | 109 | 4 | 505 | 3 | 9.58 | 8.50 | 2.50 | 4 | 1 |
| 1516 | 110 | 4 | 506 | 1 | 8.59 | 10.00 | 5.50 | 5 | 1 |
| 1517 | 110 | 4 | 506 | 2 | 9.87 | 10.50 | 6.00 | 4 | 1 |
| 1518 | 110 | 4 | 506 | 3 | 9.20 | 10.00 | 5.00 | 5 | 1 |
| 1519 | 111 | 4 | 507 | 1 | 7.70 | 9.50 | 5.50 | 5 | 1 |
| 1520 | 111 | 4 | 507 | 2 | 6.88 | 9.20 | 6.00 | 5 | 1 |
| 1521 | 111 | 4 | 507 | 3 | 8.56 | 9.70 | 6.00 | 5 | 1 |
| 1522 | 112 | 4 | 508 | 1 | 4.42 | 9.00 | 7.00 | 5 | 1 |
| 1523 | 112 | 4 | 508 | 2 | 14.67 | 11.50 | 7.00 | 5 | 1 |
| 1524 | 112 | 4 | 508 | 3 | 15.15 | 11.00 | 8.00 | 5 | 1 |
| 1525 | 113 | 4 | 509 | 1 | 10.85 | 10.50 | 6.00 | 5 | 1 |
| 1526 | 113 | 4 | 509 | 2 | 7.07 | 10.00 | 5.70 | 5 | 1 |
| 1527 | 113 | 4 | 509 | 3 | 0.00 | 0.00 | 0.00 | 0 | 0 |
| 1528 | 114 | 4 | 510 | 1 | 9.36 | 9.80 | 6.00 | 4 | 1 |
| 1529 | 114 | 4 | 510 | 2 | 8.09 | 10.00 | 6.00 | 4 | 1 |
| 1530 | 114 | 4 | 510 | 3 | 13.56 | 11.50 | 6.50 | 5 | 1 |
| 1531 | 115 | 4 | 511 | 1 | 12.32 | 9.80 | 6.00 | 4 | 1 |
| 1532 | 115 | 4 | 511 | 2 | 11.84 | 9.50 | 6.50 | 4 | 1 |
| 1533 | 115 | 4 | 511 | 3 | 7.77 | 9.00 | 7.00 | 4 | 1 |
| 1534 | 116 | 4 | 512 | 1 | 0.00 | 0.00 | 0.00 | 0 | 0 |
| 1535 | 116 | 4 | 512 | 2 | 11.14 | 9.00 | 7.00 | 5 | 1 |
| 1536 | 116 | 4 | 512 | 3 | 0.00 | 0.00 | 0.00 | 0 | 0 |
| 1537 | 117 | 4 | 513 | 1 | 2.96 | 4.30 | 3.00 | 4 | 1 |
| 1538 | 117 | 4 | 513 | 2 | 4.39 | 7.80 | 3.60 | 5 | 1 |
| 1539 | 117 | 4 | 513 | 3 | 8.66 | 8.50 | 2.50 | 4 | 1 |
| 1540 | 118 | 4 | 514 | 1 | 8.12 | 9.50 | 5.50 | 5 | 1 |
| 1541 | 118 | 4 | 514 | 2 | 7.08 | 11.00 | 3.00 | 5 | 1 |
| 1542 | 118 | 4 | 514 | 3 | 6.27 | 11.20 | 1.60 | 5 | 1 |
| 1543 | 119 | 4 | 515 | 1 | 13.85 | 12.50 | 9.50 | 5 | 1 |
| 1544 | 119 | 4 | 515 | 2 | 15.12 | 12.00 | 9.00 | 5 | 1 |
| 1545 | 119 | 4 | 515 | 3 | 15.60 | 12.30 | 9.50 | 5 | 1 |
| 1546 | 120 | 4 | 516 | 1 | 9.39 | 9.80 | 7.00 | 5 | 1 |
| 1547 | 120 | 4 | 516 | 2 | 0.00 | 0.00 | 0.00 | 0 | 0 |
| 1548 | 120 | 4 | 516 | 3 | 9.17 | 9.50 | 6.50 | 5 | 1 |
| 1549 | 121 | 4 | 517 | 1 | 7.19 | 9.00 | 6.50 | 4 | 1 |
| 1550 | 121 | 4 | 517 | 2 | 4.31 | 8.50 | 4.00 | 4 | 1 |
| 1551 | 121 | 4 | 517 | 3 | 9.33 | 8.00 | 4.50 | 5 | 1 |
| 1552 | 122 | 4 | 518 | 1 | 8.75 | 10.60 | 6.00 | 5 | 1 |
| 1553 | 122 | 4 | 518 | 2 | 7.80 | 10.00 | 6.00 | 5 | 1 |
| 1554 | 122 | 4 | 518 | 3 | 6.08 | 8.00 | 4.80 | 4 | 1 |
| 1555 | 123 | 4 | 519 | 1 | 9.07 | 8.50 | 7.00 | 5 | 1 |
| 1556 | 123 | 4 | 519 | 2 | 5.95 | 7.00 | 5.00 | 5 | 1 |
| 1557 | 123 | 4 | 519 | 3 | 2.83 | 4.50 | 2.00 | 4 | 1 |
| 1558 | 124 | 4 | 520 | 1 | 12.51 | 11.50 | 4.50 | 5 | 1 |
| 1559 | 124 | 4 | 520 | 2 | 0.00 | 0.00 | 0.00 | 0 | 0 |
| 1560 | 124 | 4 | 520 | 3 | 9.23 | 10.80 | 5.00 | 5 | 1 |
| 1561 | 125 | 4 | 521 | 1 | 7.99 | 9.00 | 6.00 | 5 | 1 |
| 1562 | 125 | 4 | 521 | 2 | 0.00 | 0.00 | 0.00 | 0 | 0 |
| 1563 | 125 | 4 | 521 | 3 | 1.91 | 1.80 | 1.50 | 5 | 1 |
| 1564 | 126 | 4 | 522 | 1 | 9.10 | 10.00 | 5.00 | 4 | 1 |
| 1565 | 126 | 4 | 522 | 2 | 15.25 | 13.50 | 5.00 | 5 | 1 |
| 1566 | 126 | 4 | 522 | 3 | 12.80 | 13.00 | 9.00 | 5 | 1 |
| 1567 | 127 | 4 | 523 | 1 | 12.99 | 9.80 | 5.50 | 5 | 1 |
| 1568 | 127 | 4 | 523 | 2 | 7.51 | 7.80 | 3.00 | 3 | 1 |
| 1569 | 127 | 4 | 523 | 3 | 0.00 | 0.00 | 0.00 | 0 | 0 |
| 1570 | 128 | 4 | 524 | 1 | 6.08 | 9.50 | 2.80 | 3 | 1 |
| 1571 | 128 | 4 | 524 | 2 | 12.41 | 8.70 | 3.00 | 3 | 1 |
| 1572 | 128 | 4 | 524 | 3 | 12.10 | 8.00 | 2.80 | 4 | 1 |
| 1573 | 129 | 4 | 525 | 1 | 6.72 | 6.70 | 4.50 | 4 | 1 |
| 1574 | 129 | 4 | 525 | 2 | 4.93 | 5.50 | 3.50 | 5 | 1 |
| 1575 | 129 | 4 | 525 | 3 | 5.79 | 6.50 | 2.80 | 4 | 1 |
| 1576 | 130 | 4 | 526 | 1 | 1.31 | 2.80 | 1.50 | 4 | 1 |
| 1577 | 130 | 4 | 526 | 2 | 10.98 | 11.00 | 6.00 | 5 | 1 |
| 1578 | 130 | 4 | 526 | 3 | 12.80 | 11.30 | 6.00 | 5 | 1 |
| 1579 | 131 | 4 | 527 | 1 | 2.80 | 3.60 | 1.30 | 5 | 1 |
| 1580 | 131 | 4 | 527 | 2 | 7.77 | 11.00 | 8.00 | 4 | 1 |
| 1581 | 131 | 4 | 527 | 3 | 1.78 | 3.00 | 1.50 | 5 | 1 |
| 1582 | 132 | 4 | 528 | 1 | 0.00 | 0.00 | 0.00 | 0 | 0 |
| 1583 | 132 | 4 | 528 | 2 | 0.00 | 0.00 | 0.00 | 0 | 0 |
| 1584 | 132 | 4 | 528 | 3 | 6.84 | 9.50 | 1.50 | 3 | 1 |
| 1585 | 1 | 5 | 529 | 1 | 13.53 | 9.50 | 7.00 | 5 | 1 |
| 1586 | 1 | 5 | 529 | 2 | 12.89 | 9.80 | 7.00 | 4 | 1 |
| 1587 | 1 | 5 | 529 | 3 | 9.80 | 9.30 | 7.50 | 4 | 1 |
| 1588 | 2 | 5 | 530 | 1 | 5.63 | 8.00 | 6.00 | 4 | 1 |
| 1589 | 2 | 5 | 530 | 2 | 0.00 | 0.00 | 0.00 | 0 | 0 |
| 1590 | 2 | 5 | 530 | 3 | 0.00 | 0.00 | 0.00 | 0 | 0 |
| 1591 | 3 | 5 | 531 | 1 | 10.19 | 9.00 | 8.00 | 4 | 1 |
| 1592 | 3 | 5 | 531 | 2 | 2.86 | 6.00 | 4.00 | 4 | 1 |
| 1593 | 3 | 5 | 531 | 3 | 7.64 | 8.00 | 7.00 | 5 | 1 |
| 1594 | 4 | 5 | 532 | 1 | 6.05 | 8.00 | 6.00 | 4 | 1 |
| 1595 | 4 | 5 | 532 | 2 | 9.01 | 9.00 | 8.00 | 5 | 1 |
| 1596 | 4 | 5 | 532 | 3 | 13.05 | 11.00 | 8.00 | 5 | 1 |
| 1597 | 5 | 5 | 533 | 1 | 8.28 | 8.00 | 7.00 | 5 | 1 |
| 1598 | 5 | 5 | 533 | 2 | 0.00 | 0.00 | 0.00 | 0 | 0 |
| 1599 | 5 | 5 | 533 | 3 | 3.18 | 6.00 | 5.00 | 4 | 1 |
| 1600 | 6 | 5 | 534 | 1 | 7.48 | 9.00 | 7.00 | 4 | 1 |
| 1601 | 6 | 5 | 534 | 2 | 9.23 | 9.00 | 7.00 | 4 | 1 |
| 1602 | 6 | 5 | 534 | 3 | 11.94 | 10.00 | 8.00 | 4 | 1 |
| 1603 | 7 | 5 | 535 | 1 | 2.04 | 4.00 | 1.80 | 3 | 1 |
| 1604 | 7 | 5 | 535 | 2 | 8.91 | 9.50 | 7.00 | 4 | 1 |
| 1605 | 7 | 5 | 535 | 3 | 11.84 | 9.50 | 7.50 | 5 | 1 |
| 1606 | 8 | 5 | 536 | 1 | 15.76 | 10.50 | 8.00 | 4 | 1 |
| 1607 | 8 | 5 | 536 | 2 | 10.35 | 10.00 | 8.30 | 5 | 1 |
| 1608 | 8 | 5 | 536 | 3 | 9.58 | 9.00 | 7.50 | 5 | 1 |
| 1609 | 9 | 5 | 537 | 1 | 0.00 | 0.00 | 0.00 | 0 | 0 |
| 1610 | 9 | 5 | 537 | 2 | 5.73 | 8.00 | 7.00 | 4 | 1 |
| 1611 | 9 | 5 | 537 | 3 | 0.00 | 0.00 | 0.00 | 0 | 0 |
| 1612 | 10 | 5 | 538 | 1 | 0.00 | 0.00 | 0.00 | 0 | 0 |
| 1613 | 10 | 5 | 538 | 2 | 10.47 | 9.00 | 7.50 | 5 | 1 |
| 1614 | 10 | 5 | 538 | 3 | 0.00 | 0.00 | 0.00 | 0 | 0 |
| 1615 | 11 | 5 | 539 | 1 | 8.98 | 10.50 | 7.00 | 5 | 1 |
| 1616 | 11 | 5 | 539 | 2 | 0.00 | 0.00 | 0.00 | 0 | 0 |
| 1617 | 11 | 5 | 539 | 3 | 9.17 | 11.00 | 7.50 | 5 | 1 |
| 1618 | 12 | 5 | 540 | 1 | 11.68 | 10.00 | 7.00 | 4 | 1 |
| 1619 | 12 | 5 | 540 | 2 | 10.35 | 9.00 | 7.00 | 4 | 1 |
| 1620 | 12 | 5 | 540 | 3 | 2.71 | 6.00 | 5.00 | 4 | 1 |
| 1621 | 13 | 5 | 541 | 1 | 4.93 | 9.00 | 5.00 | 5 | 1 |
| 1622 | 13 | 5 | 541 | 2 | 10.50 | 8.00 | 6.00 | 5 | 1 |
| 1623 | 13 | 5 | 541 | 3 | 12.57 | 9.00 | 6.50 | 4 | 1 |
| 1624 | 14 | 5 | 542 | 1 | 8.21 | 9.20 | 7.50 | 5 | 1 |
| 1625 | 14 | 5 | 542 | 2 | 11.20 | 10.00 | 7.80 | 4 | 1 |
| 1626 | 14 | 5 | 542 | 3 | 13.34 | 9.80 | 8.00 | 5 | 1 |
| 1627 | 15 | 5 | 543 | 1 | 9.17 | 9.00 | 1.80 | 4 | 1 |
| 1628 | 15 | 5 | 543 | 2 | 13.88 | 9.80 | 6.00 | 4 | 1 |
| 1629 | 15 | 5 | 543 | 3 | 6.97 | 8.40 | 6.00 | 5 | 1 |
| 1630 | 16 | 5 | 544 | 1 | 11.78 | 10.00 | 8.00 | 4 | 1 |
| 1631 | 16 | 5 | 544 | 2 | 10.19 | 11.00 | 10.00 | 5 | 1 |
| 1632 | 16 | 5 | 544 | 3 | 5.73 | 6.00 | 4.00 | 4 | 1 |
| 1633 | 17 | 5 | 545 | 1 | 0.00 | 0.00 | 0.00 | 0 | 0 |
| 1634 | 17 | 5 | 545 | 2 | 4.65 | 5.90 | 3.30 | 5 | 1 |
| 1635 | 17 | 5 | 545 | 3 | 3.25 | 4.80 | 2.30 | 5 | 1 |
| 1636 | 18 | 5 | 546 | 1 | 13.21 | 9.50 | 7.00 | 5 | 1 |
| 1637 | 18 | 5 | 546 | 2 | 11.71 | 9.00 | 7.00 | 4 | 1 |
| 1638 | 18 | 5 | 546 | 3 | 13.81 | 9.30 | 7.50 | 3 | 1 |
| 1639 | 19 | 5 | 547 | 1 | 9.93 | 10.00 | 8.50 | 5 | 1 |
| 1640 | 19 | 5 | 547 | 2 | 0.00 | 0.00 | 0.00 | 0 | 0 |
| 1641 | 19 | 5 | 547 | 3 | 3.18 | 6.00 | 3.00 | 4 | 1 |
| 1642 | 20 | 5 | 548 | 1 | 0.00 | 0.00 | 0.00 | 0 | 0 |
| 1643 | 20 | 5 | 548 | 2 | 9.58 | 9.00 | 7.00 | 5 | 1 |
| 1644 | 20 | 5 | 548 | 3 | 12.64 | 9.50 | 7.50 | 5 | 1 |
| 1645 | 21 | 5 | 549 | 1 | 5.86 | 8.00 | 5.00 | 4 | 1 |
| 1646 | 21 | 5 | 549 | 2 | 13.11 | 10.50 | 8.00 | 5 | 1 |
| 1647 | 21 | 5 | 549 | 3 | 1.43 | 2.40 | 1.80 | 5 | 1 |
| 1648 | 22 | 5 | 550 | 1 | 6.37 | 8.50 | 6.00 | 5 | 1 |
| 1649 | 22 | 5 | 550 | 2 | 7.16 | 8.00 | 6.00 | 4 | 1 |
| 1650 | 22 | 5 | 550 | 3 | 0.00 | 0.00 | 0.00 | 0 | 0 |
| 1651 | 23 | 5 | 551 | 1 | 0.00 | 0.00 | 0.00 | 0 | 0 |
| 1652 | 23 | 5 | 551 | 2 | 7.83 | 9.00 | 7.00 | 5 | 1 |
| 1653 | 23 | 5 | 551 | 3 | 6.94 | 9.30 | 7.30 | 5 | 1 |
| 1654 | 24 | 5 | 552 | 1 | 11.62 | 9.50 | 7.50 | 4 | 1 |
| 1655 | 24 | 5 | 552 | 2 | 12.10 | 10.00 | 8.00 | 5 | 1 |
| 1656 | 24 | 5 | 552 | 3 | 10.82 | 8.00 | 7.00 | 5 | 1 |
| 1657 | 25 | 5 | 553 | 1 | 10.76 | 11.00 | 8.00 | 4 | 1 |
| 1658 | 25 | 5 | 553 | 2 | 8.18 | 9.50 | 7.50 | 4 | 1 |
| 1659 | 25 | 5 | 553 | 3 | 7.77 | 9.00 | 7.00 | 4 | 1 |
| 1660 | 26 | 5 | 554 | 1 | 5.09 | 6.00 | 5.00 | 4 | 1 |
| 1661 | 26 | 5 | 554 | 2 | 2.23 | 4.00 | 3.00 | 4 | 1 |
| 1662 | 26 | 5 | 554 | 3 | 11.78 | 10.00 | 8.00 | 5 | 1 |
| 1663 | 27 | 5 | 555 | 1 | 6.75 | 7.30 | 5.50 | 5 | 1 |
| 1664 | 27 | 5 | 555 | 2 | 8.40 | 8.00 | 6.50 | 5 | 1 |
| 1665 | 27 | 5 | 555 | 3 | 9.77 | 9.00 | 7.00 | 5 | 1 |
| 1666 | 28 | 5 | 556 | 1 | 0.00 | 0.00 | 0.00 | 0 | 0 |
| 1667 | 28 | 5 | 556 | 2 | 3.21 | 7.00 | 1.30 | 4 | 1 |
| 1668 | 28 | 5 | 556 | 3 | 9.10 | 8.00 | 5.00 | 5 | 1 |
| 1669 | 29 | 5 | 557 | 1 | 8.53 | 8.00 | 7.00 | 4 | 1 |
| 1670 | 29 | 5 | 557 | 2 | 10.50 | 9.50 | 3.00 | 4 | 1 |
| 1671 | 29 | 5 | 557 | 3 | 7.38 | 9.00 | 7.00 | 5 | 1 |
| 1672 | 30 | 5 | 558 | 1 | 12.03 | 11.00 | 6.00 | 5 | 1 |
| 1673 | 30 | 5 | 558 | 2 | 4.81 | 7.50 | 5.00 | 5 | 1 |
| 1674 | 30 | 5 | 558 | 3 | 5.54 | 8.50 | 4.00 | 4 | 1 |
| 1675 | 31 | 5 | 559 | 1 | 0.00 | 0.00 | 0.00 | 0 | 0 |
| 1676 | 31 | 5 | 559 | 2 | 10.66 | 9.50 | 5.00 | 5 | 1 |
| 1677 | 31 | 5 | 559 | 3 | 14.77 | 9.50 | 7.00 | 5 | 1 |
| 1678 | 32 | 5 | 560 | 1 | 0.00 | 0.00 | 0.00 | 0 | 0 |
| 1679 | 32 | 5 | 560 | 2 | 11.01 | 9.30 | 7.50 | 5 | 1 |
| 1680 | 32 | 5 | 560 | 3 | 0.00 | 0.00 | 0.00 | 0 | 0 |
| 1681 | 33 | 5 | 561 | 1 | 16.23 | 10.50 | 3.00 | 4 | 1 |
| 1682 | 33 | 5 | 561 | 2 | 9.33 | 9.00 | 7.00 | 5 | 1 |
| 1683 | 33 | 5 | 561 | 3 | 12.03 | 10.00 | 7.50 | 5 | 1 |
| 1684 | 34 | 5 | 562 | 1 | 9.49 | 9.50 | 6.00 | 5 | 1 |
| 1685 | 34 | 5 | 562 | 2 | 7.38 | 9.50 | 7.20 | 5 | 1 |
| 1686 | 34 | 5 | 562 | 3 | 6.65 | 8.80 | 7.00 | 5 | 1 |
| 1687 | 35 | 5 | 563 | 1 | 11.94 | 12.50 | 8.00 | 5 | 1 |
| 1688 | 35 | 5 | 563 | 2 | 7.07 | 12.00 | 8.50 | 4 | 1 |
| 1689 | 35 | 5 | 563 | 3 | 9.58 | 11.50 | 8.00 | 5 | 1 |
| 1690 | 36 | 5 | 564 | 1 | 10.19 | 10.00 | 8.00 | 4 | 1 |
| 1691 | 36 | 5 | 564 | 2 | 8.91 | 7.00 | 1.50 | 4 | 1 |
| 1692 | 36 | 5 | 564 | 3 | 9.07 | 10.00 | 8.00 | 4 | 1 |
| 1693 | 37 | 5 | 565 | 1 | 9.01 | 9.50 | 7.50 | 4 | 1 |
| 1694 | 37 | 5 | 565 | 2 | 10.44 | 9.20 | 7.00 | 5 | 1 |
| 1695 | 37 | 5 | 565 | 3 | 12.89 | 9.00 | 7.50 | 5 | 1 |
| 1696 | 38 | 5 | 566 | 1 | 6.08 | 7.50 | 1.80 | 3 | 1 |
| 1697 | 38 | 5 | 566 | 2 | 9.45 | 8.00 | 5.00 | 4 | 1 |
| 1698 | 38 | 5 | 566 | 3 | 7.70 | 7.50 | 5.00 | 5 | 1 |
| 1699 | 39 | 5 | 567 | 1 | 7.51 | 8.80 | 6.50 | 5 | 1 |
| 1700 | 39 | 5 | 567 | 2 | 6.46 | 8.30 | 5.50 | 5 | 1 |
| 1701 | 39 | 5 | 567 | 3 | 7.80 | 8.50 | 6.00 | 5 | 1 |
| 1702 | 40 | 5 | 568 | 1 | 4.84 | 7.00 | 5.00 | 4 | 1 |
| 1703 | 40 | 5 | 568 | 2 | 5.86 | 8.00 | 5.50 | 4 | 1 |
| 1704 | 40 | 5 | 568 | 3 | 0.00 | 0.00 | 0.00 | 0 | 0 |
| 1705 | 41 | 5 | 569 | 1 | 12.25 | 9.50 | 7.00 | 5 | 1 |
| 1706 | 41 | 5 | 569 | 2 | 12.16 | 9.50 | 7.50 | 5 | 1 |
| 1707 | 41 | 5 | 569 | 3 | 14.61 | 9.50 | 7.50 | 5 | 1 |
| 1708 | 42 | 5 | 570 | 1 | 6.11 | 6.00 | 5.00 | 4 | 1 |
| 1709 | 42 | 5 | 570 | 2 | 5.73 | 7.00 | 6.00 | 4 | 1 |
| 1710 | 42 | 5 | 570 | 3 | 3.98 | 8.00 | 6.00 | 4 | 1 |
| 1711 | 43 | 5 | 571 | 1 | 8.75 | 10.00 | 6.50 | 4 | 1 |
| 1712 | 43 | 5 | 571 | 2 | 6.21 | 8.50 | 6.00 | 4 | 1 |
| 1713 | 43 | 5 | 571 | 3 | 8.34 | 9.00 | 6.00 | 5 | 1 |
| 1714 | 44 | 5 | 572 | 1 | 14.16 | 11.50 | 8.00 | 4 | 1 |
| 1715 | 44 | 5 | 572 | 2 | 11.97 | 11.80 | 7.50 | 4 | 1 |
| 1716 | 44 | 5 | 572 | 3 | 10.82 | 11.00 | 7.50 | 3 | 1 |
| 1717 | 45 | 5 | 573 | 1 | 13.05 | 10.00 | 7.00 | 4 | 1 |
| 1718 | 45 | 5 | 573 | 2 | 7.64 | 9.00 | 7.00 | 4 | 1 |
| 1719 | 45 | 5 | 573 | 3 | 14.64 | 10.00 | 8.00 | 5 | 1 |
| 1720 | 46 | 5 | 574 | 1 | 11.30 | 10.00 | 8.00 | 4 | 1 |
| 1721 | 46 | 5 | 574 | 2 | 7.64 | 9.00 | 7.00 | 5 | 1 |
| 1722 | 46 | 5 | 574 | 3 | 11.14 | 8.00 | 6.00 | 4 | 1 |
| 1723 | 47 | 5 | 575 | 1 | 16.07 | 10.00 | 8.00 | 5 | 1 |
| 1724 | 47 | 5 | 575 | 2 | 11.97 | 9.00 | 7.00 | 5 | 1 |
| 1725 | 47 | 5 | 575 | 3 | 9.36 | 8.00 | 4.00 | 3 | 1 |
| 1726 | 48 | 5 | 576 | 1 | 9.71 | 9.00 | 7.00 | 5 | 1 |
| 1727 | 48 | 5 | 576 | 2 | 12.10 | 10.00 | 8.00 | 4 | 1 |
| 1728 | 48 | 5 | 576 | 3 | 7.80 | 7.00 | 6.00 | 4 | 1 |
| 1729 | 49 | 5 | 577 | 1 | 9.61 | 9.00 | 5.50 | 4 | 1 |
| 1730 | 49 | 5 | 577 | 2 | 0.00 | 0.00 | 0.00 | 0 | 0 |
| 1731 | 49 | 5 | 577 | 3 | 5.67 | 7.30 | 4.00 | 5 | 1 |
| 1732 | 50 | 5 | 578 | 1 | 3.50 | 5.30 | 1.30 | 5 | 1 |
| 1733 | 50 | 5 | 578 | 2 | 5.25 | 6.50 | 1.30 | 5 | 1 |
| 1734 | 50 | 5 | 578 | 3 | 9.42 | 7.80 | 4.50 | 5 | 1 |
| 1735 | 51 | 5 | 579 | 1 | 1.43 | 2.80 | 1.30 | 5 | 1 |
| 1736 | 51 | 5 | 579 | 2 | 8.15 | 9.30 | 7.80 | 4 | 1 |
| 1737 | 51 | 5 | 579 | 3 | 6.62 | 9.00 | 6.00 | 3 | 1 |
| 1738 | 52 | 5 | 580 | 1 | 10.38 | 10.00 | 7.50 | 5 | 1 |
| 1739 | 52 | 5 | 580 | 2 | 9.80 | 9.80 | 7.80 | 5 | 1 |
| 1740 | 52 | 5 | 580 | 3 | 5.98 | 8.00 | 7.00 | 4 | 1 |
| 1741 | 53 | 5 | 581 | 1 | 0.95 | 2.30 | 1.30 | 4 | 1 |
| 1742 | 53 | 5 | 581 | 2 | 7.96 | 9.00 | 6.00 | 4 | 1 |
| 1743 | 53 | 5 | 581 | 3 | 9.39 | 8.00 | 2.00 | 4 | 1 |
| 1744 | 54 | 5 | 582 | 1 | 10.82 | 10.00 | 8.00 | 5 | 1 |
| 1745 | 54 | 5 | 582 | 2 | 5.89 | 8.00 | 7.00 | 4 | 1 |
| 1746 | 54 | 5 | 582 | 3 | 11.46 | 9.00 | 6.00 | 4 | 1 |
| 1747 | 55 | 5 | 583 | 1 | 2.51 | 4.00 | 1.80 | 3 | 1 |
| 1748 | 55 | 5 | 583 | 2 | 6.59 | 11.00 | 7.00 | 5 | 1 |
| 1749 | 55 | 5 | 583 | 3 | 0.00 | 0.00 | 0.00 | 0 | 0 |
| 1750 | 56 | 5 | 584 | 1 | 12.61 | 9.50 | 7.50 | 4 | 1 |
| 1751 | 56 | 5 | 584 | 2 | 0.00 | 0.00 | 0.00 | 0 | 0 |
| 1752 | 56 | 5 | 584 | 3 | 9.74 | 9.00 | 7.00 | 4 | 1 |
| 1753 | 57 | 5 | 585 | 1 | 0.00 | 0.00 | 0.00 | 0 | 0 |
| 1754 | 57 | 5 | 585 | 2 | 12.19 | 10.00 | 6.00 | 5 | 1 |
| 1755 | 57 | 5 | 585 | 3 | 8.85 | 9.50 | 5.50 | 4 | 1 |
| 1756 | 58 | 5 | 586 | 1 | 12.25 | 8.90 | 7.00 | 4 | 1 |
| 1757 | 58 | 5 | 586 | 2 | 8.37 | 8.00 | 5.50 | 5 | 1 |
| 1758 | 58 | 5 | 586 | 3 | 9.01 | 7.50 | 5.00 | 4 | 1 |
| 1759 | 59 | 5 | 587 | 1 | 0.00 | 0.00 | 0.00 | 0 | 0 |
| 1760 | 59 | 5 | 587 | 2 | 2.29 | 4.00 | 1.30 | 4 | 1 |
| 1761 | 59 | 5 | 587 | 3 | 12.13 | 10.50 | 8.00 | 5 | 1 |
| 1762 | 60 | 5 | 588 | 1 | 11.94 | 10.00 | 8.00 | 4 | 1 |
| 1763 | 60 | 5 | 588 | 2 | 5.33 | 9.00 | 2.00 | 4 | 1 |
| 1764 | 60 | 5 | 588 | 3 | 9.87 | 8.00 | 6.00 | 4 | 1 |
| 1765 | 61 | 5 | 589 | 1 | 8.66 | 8.00 | 5.00 | 3 | 1 |
| 1766 | 61 | 5 | 589 | 2 | 13.11 | 9.30 | 8.50 | 5 | 1 |
| 1767 | 61 | 5 | 589 | 3 | 8.31 | 9.00 | 7.00 | 4 | 1 |
| 1768 | 62 | 5 | 590 | 1 | 4.04 | 7.50 | 6.50 | 4 | 1 |
| 1769 | 62 | 5 | 590 | 2 | 5.89 | 7.00 | 5.00 | 4 | 1 |
| 1770 | 62 | 5 | 590 | 3 | 5.57 | 6.00 | 5.00 | 4 | 1 |
| 1771 | 63 | 5 | 591 | 1 | 9.71 | 9.30 | 7.00 | 5 | 1 |
| 1772 | 63 | 5 | 591 | 2 | 6.81 | 8.60 | 6.50 | 4 | 1 |
| 1773 | 63 | 5 | 591 | 3 | 8.31 | 8.70 | 7.00 | 5 | 1 |
| 1774 | 64 | 5 | 592 | 1 | 9.49 | 9.00 | 6.00 | 4 | 1 |
| 1775 | 64 | 5 | 592 | 2 | 11.62 | 10.00 | 8.00 | 4 | 1 |
| 1776 | 64 | 5 | 592 | 3 | 6.21 | 9.00 | 8.00 | 4 | 1 |
| 1777 | 65 | 5 | 593 | 1 | 0.00 | 0.00 | 0.00 | 0 | 0 |
| 1778 | 65 | 5 | 593 | 2 | 1.27 | 2.10 | 1.30 | 4 | 1 |
| 1779 | 65 | 5 | 593 | 3 | 9.39 | 10.00 | 8.00 | 4 | 1 |
| 1780 | 66 | 5 | 594 | 1 | 5.57 | 8.00 | 5.80 | 5 | 1 |
| 1781 | 66 | 5 | 594 | 2 | 3.02 | 4.00 | 1.80 | 4 | 1 |
| 1782 | 66 | 5 | 594 | 3 | 6.62 | 9.00 | 7.00 | 4 | 1 |
| 1783 | 67 | 5 | 595 | 1 | 4.52 | 6.00 | 3.50 | 5 | 1 |
| 1784 | 67 | 5 | 595 | 2 | 0.00 | 0.00 | 0.00 | 0 | 0 |
| 1785 | 67 | 5 | 595 | 3 | 0.00 | 0.00 | 0.00 | 0 | 0 |
| 1786 | 68 | 5 | 596 | 1 | 7.42 | 9.30 | 7.50 | 5 | 1 |
| 1787 | 68 | 5 | 596 | 2 | 8.66 | 8.80 | 6.50 | 4 | 1 |
| 1788 | 68 | 5 | 596 | 3 | 4.30 | 7.50 | 6.00 | 5 | 1 |
| 1789 | 69 | 5 | 597 | 1 | 8.28 | 9.00 | 6.50 | 4 | 1 |
| 1790 | 69 | 5 | 597 | 2 | 8.59 | 9.00 | 6.50 | 3 | 1 |
| 1791 | 69 | 5 | 597 | 3 | 8.79 | 8.00 | 5.00 | 4 | 1 |
| 1792 | 70 | 5 | 598 | 1 | 8.09 | 8.50 | 4.00 | 4 | 1 |
| 1793 | 70 | 5 | 598 | 2 | 6.62 | 8.50 | 6.00 | 5 | 1 |
| 1794 | 70 | 5 | 598 | 3 | 5.51 | 9.00 | 6.50 | 5 | 1 |
| 1795 | 71 | 5 | 599 | 1 | 9.36 | 10.00 | 7.00 | 4 | 1 |
| 1796 | 71 | 5 | 599 | 2 | 11.62 | 11.00 | 8.00 | 5 | 1 |
| 1797 | 71 | 5 | 599 | 3 | 9.58 | 11.30 | 8.00 | 5 | 1 |
| 1798 | 72 | 5 | 600 | 1 | 9.71 | 9.50 | 7.50 | 5 | 1 |
| 1799 | 72 | 5 | 600 | 2 | 0.00 | 0.00 | 0.00 | 0 | 0 |
| 1800 | 72 | 5 | 600 | 3 | 10.19 | 9.00 | 8.00 | 5 | 1 |
| 1801 | 73 | 5 | 601 | 1 | 12.25 | 11.50 | 3.00 | 5 | 1 |
| 1802 | 73 | 5 | 601 | 2 | 0.00 | 0.00 | 0.00 | 0 | 0 |
| 1803 | 73 | 5 | 601 | 3 | 11.14 | 12.00 | 9.00 | 5 | 1 |
| 1804 | 74 | 5 | 602 | 1 | 6.21 | 9.00 | 7.00 | 5 | 1 |
| 1805 | 74 | 5 | 602 | 2 | 4.52 | 7.50 | 4.50 | 3 | 1 |
| 1806 | 74 | 5 | 602 | 3 | 4.93 | 7.80 | 5.00 | 5 | 1 |
| 1807 | 75 | 5 | 603 | 1 | 5.41 | 7.00 | 5.00 | 4 | 1 |
| 1808 | 75 | 5 | 603 | 2 | 8.24 | 8.00 | 7.00 | 4 | 1 |
| 1809 | 75 | 5 | 603 | 3 | 0.00 | 0.00 | 0.00 | 0 | 0 |
| 1810 | 76 | 5 | 604 | 1 | 7.80 | 8.50 | 5.50 | 5 | 1 |
| 1811 | 76 | 5 | 604 | 2 | 6.88 | 8.00 | 5.00 | 4 | 1 |
| 1812 | 76 | 5 | 604 | 3 | 6.65 | 8.30 | 6.00 | 3 | 1 |
| 1813 | 77 | 5 | 605 | 1 | 16.17 | 10.00 | 7.00 | 5 | 1 |
| 1814 | 77 | 5 | 605 | 2 | 11.62 | 10.00 | 7.00 | 5 | 1 |
| 1815 | 77 | 5 | 605 | 3 | 6.94 | 9.20 | 5.50 | 4 | 1 |
| 1816 | 78 | 5 | 606 | 1 | 9.80 | 11.20 | 7.50 | 5 | 1 |
| 1817 | 78 | 5 | 606 | 2 | 8.37 | 10.00 | 7.00 | 5 | 1 |
| 1818 | 78 | 5 | 606 | 3 | 15.85 | 11.50 | 8.00 | 5 | 1 |
| 1819 | 79 | 5 | 607 | 1 | 7.23 | 8.00 | 7.00 | 5 | 1 |
| 1820 | 79 | 5 | 607 | 2 | 9.23 | 9.00 | 8.00 | 4 | 1 |
| 1821 | 79 | 5 | 607 | 3 | 0.00 | 0.00 | 0.00 | 0 | 0 |
| 1822 | 80 | 5 | 608 | 1 | 11.05 | 9.50 | 6.00 | 5 | 1 |
| 1823 | 80 | 5 | 608 | 2 | 9.74 | 9.30 | 7.30 | 4 | 1 |
| 1824 | 80 | 5 | 608 | 3 | 10.38 | 9.00 | 6.50 | 5 | 1 |
| 1825 | 81 | 5 | 609 | 1 | 7.70 | 9.00 | 7.00 | 4 | 1 |
| 1826 | 81 | 5 | 609 | 2 | 5.83 | 8.00 | 7.00 | 4 | 1 |
| 1827 | 81 | 5 | 609 | 3 | 6.08 | 8.00 | 6.00 | 4 | 1 |
| 1828 | 82 | 5 | 610 | 1 | 8.12 | 8.00 | 6.00 | 4 | 1 |
| 1829 | 82 | 5 | 610 | 2 | 10.35 | 9.00 | 7.00 | 4 | 1 |
| 1830 | 82 | 5 | 610 | 3 | 1.59 | 3.00 | 2.00 | 4 | 1 |
| 1831 | 83 | 5 | 611 | 1 | 0.00 | 0.00 | 0.00 | 0 | 0 |
| 1832 | 83 | 5 | 611 | 2 | 8.69 | 9.00 | 7.50 | 5 | 1 |
| 1833 | 83 | 5 | 611 | 3 | 10.57 | 9.30 | 7.80 | 3 | 1 |
| 1834 | 84 | 5 | 612 | 1 | 11.78 | 9.00 | 8.00 | 5 | 1 |
| 1835 | 84 | 5 | 612 | 2 | 10.03 | 10.00 | 8.00 | 4 | 1 |
| 1836 | 84 | 5 | 612 | 3 | 4.70 | 8.00 | 2.00 | 4 | 1 |
| 1837 | 85 | 5 | 613 | 1 | 7.51 | 9.50 | 6.00 | 5 | 1 |
| 1838 | 85 | 5 | 613 | 2 | 0.00 | 0.00 | 0.00 | 0 | 0 |
| 1839 | 85 | 5 | 613 | 3 | 9.68 | 10.00 | 5.50 | 5 | 1 |
| 1840 | 86 | 5 | 614 | 1 | 1.97 | 2.50 | 2.00 | 5 | 1 |
| 1841 | 86 | 5 | 614 | 2 | 5.73 | 7.00 | 5.50 | 5 | 1 |
| 1842 | 86 | 5 | 614 | 3 | 0.00 | 0.00 | 0.00 | 0 | 0 |
| 1843 | 87 | 5 | 615 | 1 | 9.36 | 9.00 | 7.00 | 5 | 1 |
| 1844 | 87 | 5 | 615 | 2 | 2.80 | 5.00 | 4.00 | 5 | 1 |
| 1845 | 87 | 5 | 615 | 3 | 5.35 | 8.00 | 6.00 | 4 | 1 |
| 1846 | 88 | 5 | 616 | 1 | 7.45 | 8.00 | 4.00 | 4 | 1 |
| 1847 | 88 | 5 | 616 | 2 | 0.00 | 0.00 | 0.00 | 0 | 0 |
| 1848 | 88 | 5 | 616 | 3 | 4.71 | 7.00 | 5.00 | 4 | 1 |
| 1849 | 89 | 5 | 617 | 1 | 6.03 | 9.80 | 2.00 | 4 | 1 |
| 1850 | 89 | 5 | 617 | 2 | 10.70 | 10.00 | 7.50 | 5 | 1 |
| 1851 | 89 | 5 | 617 | 3 | 8.94 | 9.50 | 7.50 | 5 | 1 |
| 1852 | 90 | 5 | 618 | 1 | 0.00 | 0.00 | 0.00 | 0 | 0 |
| 1853 | 90 | 5 | 618 | 2 | 3.02 | 4.00 | 3.00 | 4 | 1 |
| 1854 | 90 | 5 | 618 | 3 | 0.00 | 0.00 | 0.00 | 0 | 0 |
| 1855 | 91 | 5 | 619 | 1 | 9.39 | 9.00 | 6.00 | 5 | 1 |
| 1856 | 91 | 5 | 619 | 2 | 4.65 | 7.00 | 5.50 | 3 | 1 |
| 1857 | 91 | 5 | 619 | 3 | 8.24 | 9.50 | 6.00 | 4 | 1 |
| 1858 | 92 | 5 | 620 | 1 | 8.21 | 8.00 | 5.00 | 4 | 1 |
| 1859 | 92 | 5 | 620 | 2 | 0.00 | 0.00 | 0.00 | 0 | 0 |
| 1860 | 92 | 5 | 620 | 3 | 12.80 | 9.00 | 6.00 | 5 | 1 |
| 1861 | 93 | 5 | 621 | 1 | 7.32 | 6.00 | 5.00 | 4 | 1 |
| 1862 | 93 | 5 | 621 | 2 | 6.84 | 7.00 | 6.00 | 4 | 1 |
| 1863 | 93 | 5 | 621 | 3 | 8.59 | 7.00 | 6.00 | 4 | 1 |
| 1864 | 94 | 5 | 622 | 1 | 10.19 | 9.50 | 7.50 | 4 | 1 |
| 1865 | 94 | 5 | 622 | 2 | 11.30 | 9.00 | 6.00 | 5 | 1 |
| 1866 | 94 | 5 | 622 | 3 | 3.98 | 7.00 | 1.30 | 4 | 1 |
| 1867 | 95 | 5 | 623 | 1 | 10.54 | 8.50 | 5.00 | 5 | 1 |
| 1868 | 95 | 5 | 623 | 2 | 4.30 | 5.50 | 3.50 | 3 | 1 |
| 1869 | 95 | 5 | 623 | 3 | 11.62 | 10.00 | 8.00 | 5 | 1 |
| 1870 | 96 | 5 | 624 | 1 | 11.43 | 10.80 | 7.50 | 5 | 1 |
| 1871 | 96 | 5 | 624 | 2 | 9.80 | 10.50 | 8.00 | 3 | 1 |
| 1872 | 96 | 5 | 624 | 3 | 9.61 | 10.00 | 7.00 | 4 | 1 |
| 1873 | 97 | 5 | 625 | 1 | 8.59 | 9.00 | 4.00 | 5 | 1 |
| 1874 | 97 | 5 | 625 | 2 | 6.94 | 8.50 | 4.50 | 5 | 1 |
| 1875 | 97 | 5 | 625 | 3 | 6.84 | 8.00 | 4.50 | 4 | 1 |
| 1876 | 98 | 5 | 626 | 1 | 3.02 | 6.00 | 5.00 | 4 | 1 |
| 1877 | 98 | 5 | 626 | 2 | 6.40 | 8.00 | 7.00 | 4 | 1 |
| 1878 | 98 | 5 | 626 | 3 | 9.71 | 10.00 | 8.00 | 5 | 1 |
| 1879 | 99 | 5 | 627 | 1 | 12.41 | 4.50 | 4.00 | 4 | 1 |
| 1880 | 99 | 5 | 627 | 2 | 0.00 | 0.00 | 0.00 | 0 | 0 |
| 1881 | 99 | 5 | 627 | 3 | 0.00 | 0.00 | 0.00 | 0 | 0 |
| 1882 | 100 | 5 | 628 | 1 | 11.94 | 11.00 | 9.00 | 4 | 1 |
| 1883 | 100 | 5 | 628 | 2 | 10.35 | 10.00 | 8.00 | 4 | 1 |
| 1884 | 100 | 5 | 628 | 3 | 13.21 | 12.00 | 9.00 | 5 | 1 |
| 1885 | 101 | 5 | 629 | 1 | 0.00 | 0.00 | 0.00 | 0 | 0 |
| 1886 | 101 | 5 | 629 | 2 | 6.84 | 8.50 | 4.00 | 5 | 1 |
| 1887 | 101 | 5 | 629 | 3 | 0.00 | 0.00 | 0.00 | 0 | 0 |
| 1888 | 102 | 5 | 630 | 1 | 10.47 | 8.50 | 5.00 | 4 | 1 |
| 1889 | 102 | 5 | 630 | 2 | 8.40 | 8.30 | 5.50 | 4 | 1 |
| 1890 | 102 | 5 | 630 | 3 | 6.02 | 7.30 | 5.00 | 4 | 1 |
| 1891 | 103 | 5 | 631 | 1 | 7.00 | 8.50 | 2.00 | 4 | 1 |
| 1892 | 103 | 5 | 631 | 2 | 9.07 | 9.00 | 7.00 | 4 | 1 |
| 1893 | 103 | 5 | 631 | 3 | 4.46 | 7.00 | 4.00 | 4 | 1 |
| 1894 | 104 | 5 | 632 | 1 | 7.16 | 8.00 | 7.00 | 4 | 1 |
| 1895 | 104 | 5 | 632 | 2 | 11.05 | 9.00 | 6.00 | 5 | 1 |
| 1896 | 104 | 5 | 632 | 3 | 10.66 | 8.00 | 5.00 | 5 | 1 |
| 1897 | 105 | 5 | 633 | 1 | 0.00 | 0.00 | 0.00 | 0 | 0 |
| 1898 | 105 | 5 | 633 | 2 | 10.35 | 8.00 | 6.00 | 5 | 1 |
| 1899 | 105 | 5 | 633 | 3 | 11.30 | 10.00 | 7.00 | 4 | 1 |
| 1900 | 106 | 5 | 634 | 1 | 13.15 | 9.00 | 7.00 | 4 | 1 |
| 1901 | 106 | 5 | 634 | 2 | 13.88 | 9.30 | 7.50 | 5 | 1 |
| 1902 | 106 | 5 | 634 | 3 | 11.65 | 9.00 | 7.00 | 5 | 1 |
| 1903 | 107 | 5 | 635 | 1 | 5.41 | 8.00 | 6.00 | 5 | 1 |
| 1904 | 107 | 5 | 635 | 2 | 8.91 | 10.00 | 8.00 | 5 | 1 |
| 1905 | 107 | 5 | 635 | 3 | 1.27 | 2.60 | 1.60 | 5 | 1 |
| 1906 | 108 | 5 | 636 | 1 | 6.97 | 9.50 | 7.50 | 4 | 1 |
| 1907 | 108 | 5 | 636 | 2 | 9.96 | 9.20 | 7.00 | 3 | 1 |
| 1908 | 108 | 5 | 636 | 3 | 4.62 | 6.00 | 4.00 | 5 | 1 |
| 1909 | 109 | 5 | 637 | 1 | 10.35 | 8.50 | 6.50 | 4 | 1 |
| 1910 | 109 | 5 | 637 | 2 | 1.91 | 3.00 | 2.00 | 4 | 1 |
| 1911 | 109 | 5 | 637 | 3 | 1.75 | 2.50 | 1.50 | 4 | 1 |
| 1912 | 110 | 5 | 638 | 1 | 8.24 | 9.00 | 6.50 | 2 | 1 |
| 1913 | 110 | 5 | 638 | 2 | 1.64 | 3.40 | 2.00 | 5 | 1 |
| 1914 | 110 | 5 | 638 | 3 | 15.95 | 9.30 | 6.00 | 5 | 1 |
| 1915 | 111 | 5 | 639 | 1 | 5.86 | 8.50 | 6.00 | 4 | 1 |
| 1916 | 111 | 5 | 639 | 2 | 7.51 | 10.00 | 7.00 | 5 | 1 |
| 1917 | 111 | 5 | 639 | 3 | 9.68 | 10.50 | 7.50 | 5 | 1 |
| 1918 | 112 | 5 | 640 | 1 | 0.00 | 0.00 | 0.00 | 0 | 0 |
| 1919 | 112 | 5 | 640 | 2 | 4.77 | 6.00 | 2.00 | 4 | 1 |
| 1920 | 112 | 5 | 640 | 3 | 7.64 | 10.00 | 8.00 | 4 | 1 |
| 1921 | 113 | 5 | 641 | 1 | 0.00 | 0.00 | 0.00 | 0 | 0 |
| 1922 | 113 | 5 | 641 | 2 | 13.75 | 10.00 | 8.00 | 5 | 1 |
| 1923 | 113 | 5 | 641 | 3 | 5.03 | 7.50 | 6.50 | 5 | 1 |
| 1924 | 114 | 5 | 642 | 1 | 9.23 | 10.00 | 9.00 | 5 | 1 |
| 1925 | 114 | 5 | 642 | 2 | 10.25 | 10.00 | 8.00 | 5 | 1 |
| 1926 | 114 | 5 | 642 | 3 | 9.64 | 9.00 | 8.00 | 5 | 1 |
| 1927 | 115 | 5 | 643 | 1 | 0.00 | 0.00 | 0.00 | 0 | 0 |
| 1928 | 115 | 5 | 643 | 2 | 9.29 | 9.00 | 7.00 | 5 | 1 |
| 1929 | 115 | 5 | 643 | 3 | 7.58 | 9.00 | 5.50 | 5 | 1 |
| 1930 | 116 | 5 | 644 | 1 | 14.58 | 11.50 | 7.50 | 5 | 1 |
| 1931 | 116 | 5 | 644 | 2 | 5.00 | 8.00 | 4.00 | 4 | 1 |
| 1932 | 116 | 5 | 644 | 3 | 8.31 | 9.50 | 6.00 | 3 | 1 |
| 1933 | 117 | 5 | 645 | 1 | 13.69 | 10.30 | 7.00 | 5 | 1 |
| 1934 | 117 | 5 | 645 | 2 | 10.28 | 9.00 | 7.00 | 5 | 1 |
| 1935 | 117 | 5 | 645 | 3 | 9.23 | 8.00 | 6.00 | 5 | 1 |
| 1936 | 118 | 5 | 646 | 1 | 4.52 | 6.00 | 2.00 | 3 | 1 |
| 1937 | 118 | 5 | 646 | 2 | 8.21 | 10.00 | 6.50 | 5 | 1 |
| 1938 | 118 | 5 | 646 | 3 | 8.21 | 10.30 | 6.00 | 5 | 1 |
| 1939 | 119 | 5 | 647 | 1 | 10.12 | 8.70 | 6.70 | 5 | 1 |
| 1940 | 119 | 5 | 647 | 2 | 9.10 | 8.80 | 7.00 | 5 | 1 |
| 1941 | 119 | 5 | 647 | 3 | 11.90 | 9.30 | 7.30 | 5 | 1 |
| 1942 | 120 | 5 | 648 | 1 | 0.00 | 0.00 | 0.00 | 0 | 0 |
| 1943 | 120 | 5 | 648 | 2 | 9.87 | 10.00 | 6.00 | 5 | 1 |
| 1944 | 120 | 5 | 648 | 3 | 11.20 | 10.00 | 6.00 | 5 | 1 |
| 1945 | 121 | 5 | 649 | 1 | 2.04 | 3.80 | 1.80 | 4 | 1 |
| 1946 | 121 | 5 | 649 | 2 | 4.39 | 5.50 | 4.00 | 5 | 1 |
| 1947 | 121 | 5 | 649 | 3 | 7.83 | 8.00 | 5.00 | 5 | 1 |
| 1948 | 122 | 5 | 650 | 1 | 12.73 | 10.00 | 8.00 | 4 | 1 |
| 1949 | 122 | 5 | 650 | 2 | 7.64 | 8.00 | 6.50 | 4 | 1 |
| 1950 | 122 | 5 | 650 | 3 | 11.14 | 9.00 | 8.00 | 4 | 1 |
| 1951 | 123 | 5 | 651 | 1 | 7.48 | 6.00 | 5.00 | 5 | 1 |
| 1952 | 123 | 5 | 651 | 2 | 8.91 | 7.00 | 6.00 | 5 | 1 |
| 1953 | 123 | 5 | 651 | 3 | 9.23 | 8.00 | 6.00 | 5 | 1 |
| 1954 | 124 | 5 | 652 | 1 | 12.25 | 9.00 | 7.00 | 5 | 1 |
| 1955 | 124 | 5 | 652 | 2 | 9.07 | 8.00 | 7.00 | 4 | 1 |
| 1956 | 124 | 5 | 652 | 3 | 8.91 | 8.00 | 7.00 | 4 | 1 |
| 1957 | 125 | 5 | 653 | 1 | 6.27 | 6.50 | 4.50 | 5 | 1 |
| 1958 | 125 | 5 | 653 | 2 | 0.00 | 0.00 | 0.00 | 0 | 0 |
| 1959 | 125 | 5 | 653 | 3 | 0.00 | 0.00 | 0.00 | 0 | 0 |
| 1960 | 126 | 5 | 654 | 1 | 10.57 | 10.00 | 6.00 | 3 | 1 |
| 1961 | 126 | 5 | 654 | 2 | 11.94 | 10.00 | 5.50 | 5 | 1 |
| 1962 | 126 | 5 | 654 | 3 | 0.00 | 0.00 | 0.00 | 0 | 0 |
| 1963 | 127 | 5 | 655 | 1 | 0.00 | 0.00 | 0.00 | 0 | 0 |
| 1964 | 127 | 5 | 655 | 2 | 0.00 | 0.00 | 0.00 | 0 | 0 |
| 1965 | 127 | 5 | 655 | 3 | 0.00 | 0.00 | 0.00 | 0 | 0 |
| 1966 | 128 | 5 | 656 | 1 | 9.87 | 9.00 | 7.00 | 5 | 1 |
| 1967 | 128 | 5 | 656 | 2 | 15.28 | 10.00 | 7.00 | 4 | 1 |
| 1968 | 128 | 5 | 656 | 3 | 0.00 | 0.00 | 0.00 | 0 | 0 |
| 1969 | 129 | 5 | 657 | 1 | 10.66 | 9.00 | 8.00 | 4 | 1 |
| 1970 | 129 | 5 | 657 | 2 | 10.98 | 8.50 | 6.50 | 4 | 1 |
| 1971 | 129 | 5 | 657 | 3 | 14.64 | 9.00 | 8.00 | 4 | 1 |
| 1972 | 130 | 5 | 658 | 1 | 13.34 | 10.30 | 6.00 | 4 | 1 |
| 1973 | 130 | 5 | 658 | 2 | 6.11 | 8.50 | 4.50 | 4 | 1 |
| 1974 | 130 | 5 | 658 | 3 | 10.47 | 9.00 | 2.20 | 5 | 1 |
| 1975 | 131 | 5 | 659 | 1 | 12.25 | 9.00 | 7.00 | 5 | 1 |
| 1976 | 131 | 5 | 659 | 2 | 11.81 | 9.50 | 7.00 | 5 | 1 |
| 1977 | 131 | 5 | 659 | 3 | 9.29 | 8.50 | 6.50 | 5 | 1 |
| 1978 | 132 | 5 | 660 | 1 | 3.02 | 5.00 | 4.00 | 4 | 1 |
| 1979 | 132 | 5 | 660 | 2 | 3.18 | 5.00 | 4.00 | 4 | 1 |
| 1980 | 132 | 5 | 660 | 3 | 0.95 | 2.50 | 1.50 | 4 | 1 |
